# Synthesis of extended fluorinated tripeptides based on the tetrahydropyridazine scaffold

**DOI:** 10.3762/bjoc.20.262

**Published:** 2024-12-04

**Authors:** Thierry Milcent, Pascal Retailleau, Benoit Crousse, Sandrine Ongeri

**Affiliations:** 1 UMR 8076, BioCIS, CNRS, Université Paris-Saclay, avenue des sciences, 91400 Orsay, France; 2 Université Paris-Saclay, CNRS, Institut de Chimie des Substances Naturelles, 91198 Gif-sur-Yvette, France

**Keywords:** fluoroalkyl groups, heterocycles, hydrazino acids, peptides, tetrahydropyridazines

## Abstract

The synthesis of tripeptides incorporating new fluorinated heterocyclic hydrazino acids, based on the tetrahydropyridazine scaffold is described. Starting from simple fluorinated hydrazones, these non-proteinogenic cyclic β-amino acids were easily prepared by a zinc-catalyzed aza-Barbier reaction followed by an intramolecular Michael addition. Preliminary conformational studies on tripeptides including this scaffold in the central position show an extended conformation in solution (NMR) and in the solid state (X-ray).

## Introduction

**Figure 1:**
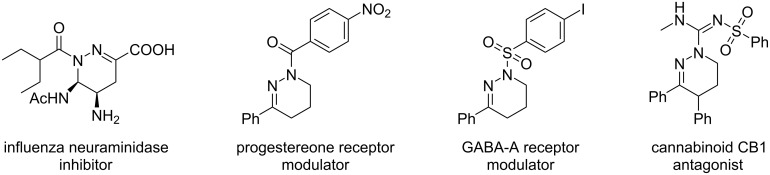
Examples of bioactive tetrahydropyridazine derivatives.

The synthesis of molecules capable of mimicking the various secondary structures and key functions of proteins is a major challenge in medicinal chemistry, especially in the fields of protein–protein interactions [1–2]. Accordingly, the incorporation of heterocyclic amino acids into peptides stabilizes their secondary structure and their metabolic stability, which is useful for numerous applications [3–5]. Indeed, the cyclic structure considerably reduces the number of possible rotational conformers, allowing a rational control of the 3D conformational space. Among these cyclic structures, the tetrahydropyridazines, six-atom nitrogenous heterocycles, are found in various bioactive molecules such as influenza virus neuraminidase inhibitors, GABA type A receptor modulators, and regulators of progesterone receptor or cannabinoid CB1 receptor antagonists (Figure 1) [6–9].

**Figure 2:**
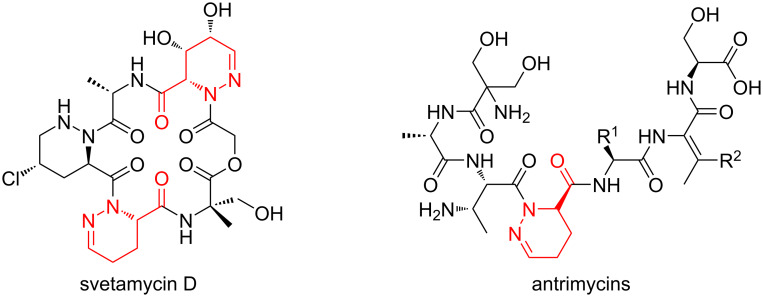
Linear and cyclic peptides incorporating the dehydropiperazic acid moiety.

This tetrahydropyridazine scaffold is also found in numerous natural linear or cyclic peptides such as svetamycins or antrimycins as dehydropiperazic acid (Figure 2) [10].

**Figure 3:**
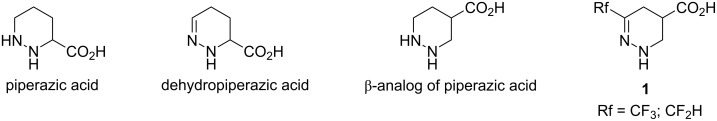
Piperazic acid and analogues and target trifluoro/difluoromethylated tetrahydropyridazine acids.

Whereas many publications have been devoted to the synthesis and structure of piperazic acid derivatives (dehydro, chloro, hydroxy, …) [11–12], nothing is known about its β-analog (Figure 3), although β-amino acids have been shown to strongly modulate the structural, metabolic, and biological characteristics of peptides [13].

Finally, it is well known that fluorine is a very useful tool in medicinal chemistry as the incorporation of fluorinated groups (CF_3_ or CF_2_H) in organic molecules can modulate their physicochemical (p*K*_a_, lipophilicity), structural (additional hydrophobic and hydrogen-bond interactions), and biological properties (metabolic stability, membrane permeability) [14–15]. Alongside the very well-known CF_3_ group, the CF_2_H group has become an essential structural motif in medicinal chemistry due to its hydrogen-bond donor capacity, its lipophilic character, and as a bioisostere for alcohol, thiol, or amine groups [16–19]. Thus, the contribution of fluorinated compounds to pharmaceuticals has been crucial for more than half a century [20].

In this context and in our ongoing effort to synthesize fluorinated non-proteinogenic linear or cyclic β-amino acids [21–22], it appeared attractive to build fluorinated β-analogs of dehydropiperazic acid (Figure 3). This novel fluorinated amino acid **1** will combine the electronic and structural properties of the fluorinated groups (CF_3_ or CF_2_H) and the geometric constraints due to its partially unsaturated cycle, which could help in the design of peptidomimetics.

**Scheme 1:**
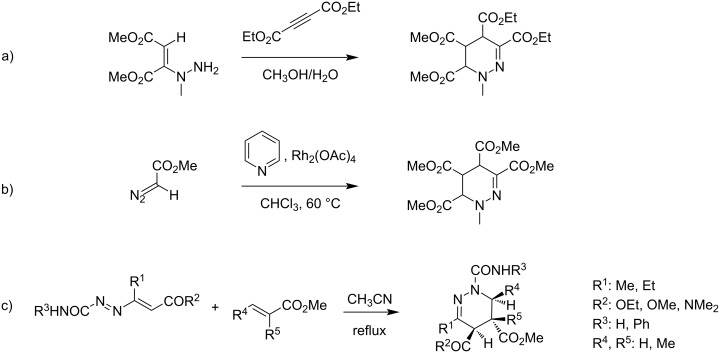
Reported syntheses of tetrahydropyridazine ester derivatives.

To our knowledge, only a few publications report the synthesis of tetrahydropyridazines with a carboxylic acid or ester substituent. Firstly, the group of Haupt reported the synthesis of ethyl esters of tetrahydromethylpyridazine in 20% yield in a mixture of methanol and water by the reaction of methylhydrazine with acetylene dicarboxylic esters through the formation of enhydrazine (Scheme 1a), [23]. Later, Tomilov et al. described the formation of tetrahydropyridazine 3,4,5,6-tetracarboxylic esters in 42% yield upon the decomposition in chloroform at 60 °C of methyl diazoacetate in the presence of pyridine and catalyzed by rhodium(II) acetate (Scheme 1b) [24–25]. More recently, an unusual [4 + 2]-cycloaddition reaction between electron-poor 1,2-diaza-1,3-dienes and electron-poor alkenes in refluxing acetonitrile was reported leading to various substituted tetrahydropyridazines in 17–78% yields (Scheme 1c) [26–27].

**Figure 4:**
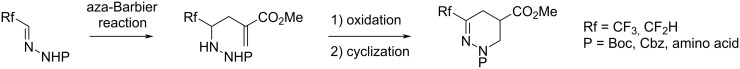
Synthetic strategy to obtain fluorinated tetrahydropyridazines from difluoro- or trifluoromethylated hydrazones.

Nevertheless, these methods are neither relevant for the synthesis of **1** nor appropriate for peptide synthesis. Consequently, developing a simple and efficient methodology is still challenging. Our new strategy to synthesize these compounds is based on an aza-Barbier reaction on difluoro- or trifluoromethylated hydrazones. The thus obtained compounds will then be oxidized and cyclized in order to lead to the expected fluorinated tetrahydropyridazines (Figure 4).

## Results and Discussion

**Scheme 2:**
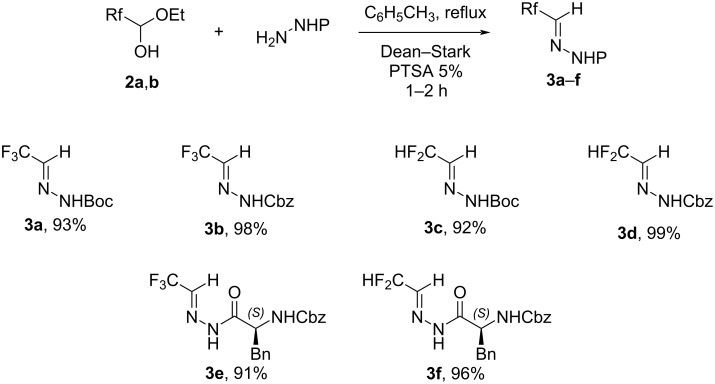
Synthesis of fluorinated hydrazones **3a**–**f**.

First, the difluoro- and trifluoromethylated hydrazones **3a**–**f** were synthesized by condensing the corresponding hydrazide with the fluorinated aldehyde hemiacetal **2a** or **2b** (Scheme 2). Benzyl and *tert*-butyl carbazate (NH_2_-NHCbz/NH_2_-NHBoc) were chosen as starting materials in order to obtain final building blocks suitable for peptide synthesis. While the synthesis of compound **3a** was already reported [28], compounds **3b**–**f** are not described in the literature. All the fluorinated hydrazones were obtained in good yields and used directly in the next step without further purification (Scheme 2). In the case of Boc-protected hydrazones, the reaction must be carefully followed and reacted less than 2 hours in order to avoid the cleavage of the Boc group. The hydrazones **3e** and **3f** substituted by *N*-Cbz-ʟ-phenylalanine could easily be synthesized under the same reaction conditions starting from the corresponding hydrazide amino acid. Compounds **3e** and **3f** were obtained as a mixture of conformers (1:1 ratio) which is often observed with *N*-acylhydrazones [29–31]. Indeed, in theory, *N*-acylhydrazones can exhibit four isomers, two due to the *E* and *Z* isomers of the imine group (–N=CH–) and two due to the *syn/anti*-conformers of the amide bond (–NH-CO–). Experimentally, the *E* isomer is often more stable and so, predominant. The strong correlation between the NH and CH of the imine observed in 2D ^1^H-^1^H NOE experiments for the two conformers of hydrazones **3e** and **3f** (see Supporting Information File 1) is in accordance with the *E* stereoisomers. Furthermore, another correlation is observed for one conformer involving the NH of the imine on one side and the α-proton and the CH_2_ of the Cbz of the phenylalanine on the other. This correlation is present for one conformer (*anti*) and not for the other (*syn*), and this observation is similar for hydrazones **3e** and **3f** (see Supporting Information File 1). Based on these experimental data we can hypothesize the geometry of the two conformers of hydrazones **3e** and **3f** is *E,syn* and *E,anti*.

**Scheme 3:**
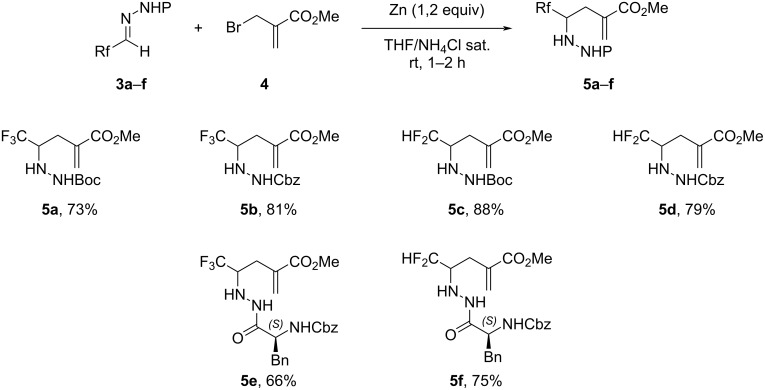
Allylation of fluorinated hydrazones **3a**–**f** to obtain **5a**–**f**.

Then, hydrazones **3** were reacted with zinc and methyl 2-(bromomethyl)acrylate (**4**). This aza-Barbier reaction was performed in a biphasic medium (THF/aqueous solution of saturated NH_4_Cl) to avoid the formation of the α-methylene-γ-lactam obtained by intramolecular cyclization of the zinc amide on the ester function, as previously reported [32–35]. The corresponding adducts **5a**–**f** were isolated with good yields from 66 to 88%. In the case of hydrazides **5e** and **5f**, the mixture of diastereomers (1:1 ratio) could not be separated at this stage. Although no stereoselectivity is observed, it can be noticed that the presence of an amino acid is compatible with the conditions of the reaction and did not interfere or significantly decrease the yield of the reaction (Scheme 3).

**Scheme 4:**
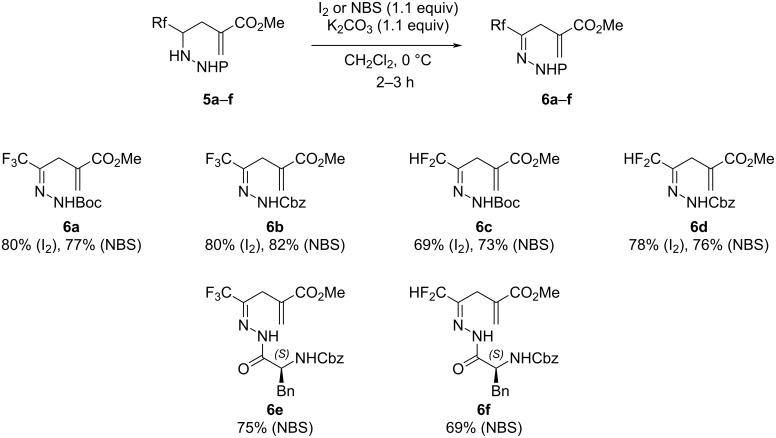
Oxidation of hydrazines **5a**–**f** to obtain hydrazones **6a**–**f**.

Then, the *N*-carboxylate hydrazides **5a**–**d** were firstly oxidized with iodine in the presence of potassium carbonate to lead to the corresponding hydrazones **6a**–**d** in good yields (69–80%). Surprisingly, these conditions were unsuitable for compounds **5e** and **5f** and led to the formation of numerous byproducts. Fortunately, the replacement of iodine with *N*-bromosuccinimide (NBS), previously reported for the oxidation of hydrazine [36], allowed the expected compounds **6e** and **6f** to be obtained in good yields. This methodology was applied to the previous hydrazides **5a**–**d** giving the corresponding compounds **6a**–**d** in similar yields. As expected, no isomerization occurred during the oxidation, leading exclusively to the imine and not the enamine. As for the hydrazones **3e** and **3f**, we assumed that the hydrazones **6e** and **6f** were obtained as a mixture of conformers, *E,syn* and *E,anti* (Scheme 4). Surprisingly, the ratio of each conformer differs for hydrazones **6e** (ratio 77:23) and **6f** (ratio 52:48).

**Scheme 5:**
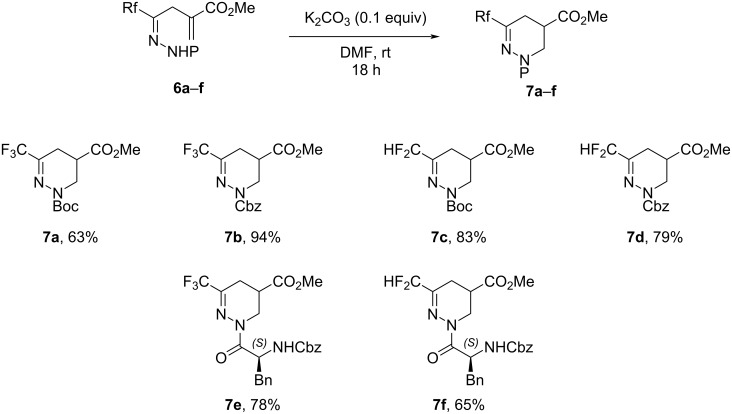
Intramolecular cyclization of compounds **6a**–**f** to obtain tetrahydropyridazines **7a**–**f**.

The last cyclization step was based on an intramolecular Michael addition carried out in DMF and catalyzed by 10% of potassium carbonate. As previously observed [37], under these conditions, the 5-*endo-trig* cyclization was selective and the lactam resulting from the 5-*exo-trig* cyclization was not observed. Furthermore, in the case of compounds **7e** and **7f**, no competition with the NH of the phenylalanine was observed. The corresponding tetrahydropyridazines **7a**–**f** were obtained in moderate to good yields (Scheme 5).

Concerning compounds **7e** and **7f**, each diastereomer of the 1:1 ratio mixture could be isolated after purification by flash silica chromatography.

**Scheme 6:**
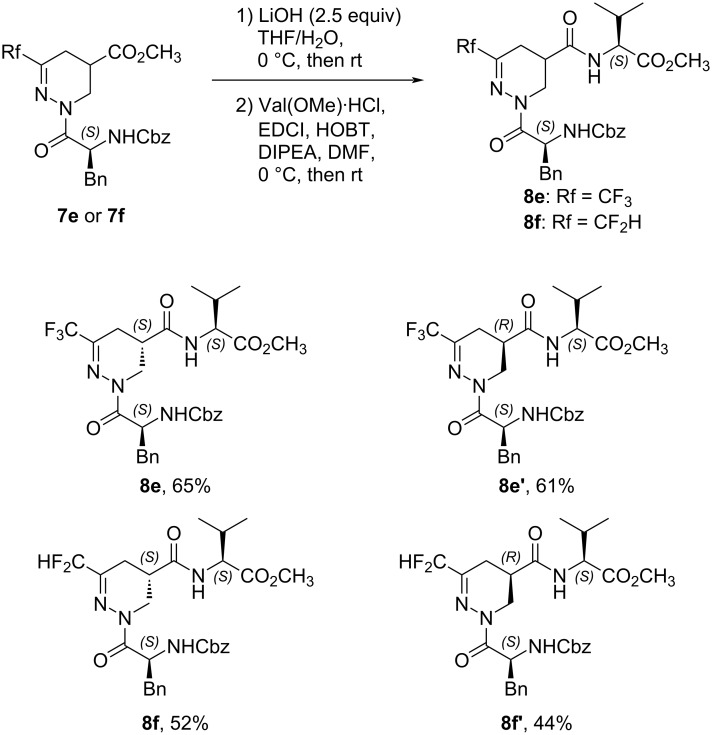
Preparation of tripeptides **8e**, **8e’**, **8f**, and **8f’**. Yields refer to the yield over 2 steps.

With dipeptides **7e** and **7f** stereoisomerically pure, we next focused our attention on the preparation of novel peptidic structures to perform some conformational analyses. Starting from the methyl ester **7**, each diastereomer was engaged in a classical sequence of saponification in the presence of LiOH, followed by a coupling reaction with ʟ-valine methyl ester hydrochloride, to give the corresponding four enantiomerically pure tripeptides **8** with satisfactory yields over two steps (Scheme 6).

**Figure 5:**
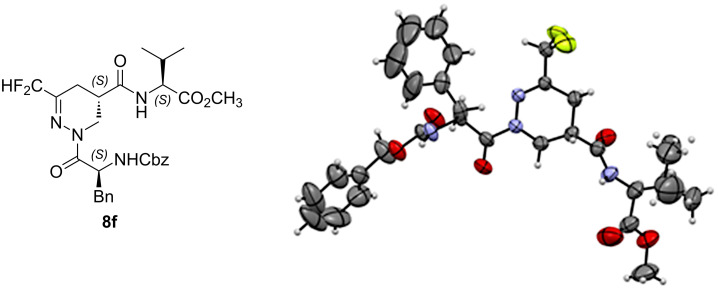
X-ray diffraction of compound **8f**.

The absolute configuration (*S,S,S*) of the isomer **8f** was unambiguously assigned by X-ray crystallographic analysis (Figure 5).

Consequently, it was possible to assess the stereochemistry of the other diastereoisomer **8f’** and their precursors **7f** and **7f’**. On the other hand, considering the similarities of the ^1^H NMR spectra of the CF_2_H and CF_3_ analogs, by comparison, we could hypothesize the absolute configuration of compounds **7e** and **7e’** and consequently of tripeptides **8e** and **8e’** (see Supporting Information File 1).

Next, some preliminary conformational studies were performed. Firstly, the X-ray crystallographic analysis of compound **8f** did not show any hydrogen-bond pattern and the global structure of the tripeptide is extended. Secondly, 2D ^1^H-^1^H NOE experiments of compound **8f** confirmed this result. Indeed, no correlation was observed between the protons of the side chain of the phenylalanine and those of the valine, suggesting an extended structure in accordance with the X-ray structure. Furthermore, the 2D ^19^F,^1^H NOE experiments of compound **8f** did not show any specific correlation between fluorine atoms and the protons of the amino acids (see Supporting Information File 1). Finally, the low chemical shifts of the amide and carbamate protons (6.4 ppm for NH of valine and 5.5 ppm for the NH of phenylalanine) indicate that they are not involved in hydrogen bonds. Furthermore, the 2D ^1^H-^1^H NOE NMR spectra and the chemical shifts of the NH protons of compounds **8f’**, **8e**, and **8e’** did not show any significant differences compared to **8f**. Overall, the ability of the new fluorinated β-analogs of dehydropiperazic acid to act as β or γ-turn is excluded. Interestingly, this novel scaffold rather promotes extended structures.

## Conclusion

To conclude, we have developed a new methodology to synthesize β-analogs of dehydropiperazic acid incorporating fluorinated groups. In order to improve the efficiency of this strategy, the control of the stereoselectivity of the intramolecular aza-Michael addition could be envisaged with various chiral catalysts in further studies. These heterocyclic hydrazino acids, when incorporated into the peptidic structure, appear to confer an extended conformation. These interesting feature results will be further confirmed by the insertion of these cyclic β-dehydropiperazic acids in longer peptide sequences.

## Supporting Information

File 1 Experimental procedures, product characterization, X-ray analysis and copies of NMR spectra.

## Data Availability

Additional research data generated and analyzed during this study is not shared.
