# Supplementary material for: Synthesis of extended fluorinated tripeptides based on the tetrahydropyridazine scaffold
Source: Beilstein J Org Chem. 2024 Dec 4;20:3174–81. doi: 10.3762/bjoc.20.262 (PMC11635284; doi:10.3762/bjoc.20.262)

BEILSTEIN JOURNAL OF ORGANIC CHEMISTRY

Supporting Information

for

Synthesis of extended fluorinated tripeptides based on the tetrahydropyridazine scaffold

Thierry Milcent, Pascal Retailleau, Benoit Crousse and Sandrine Ongeri

Beilstein J. Org. Chem. **2024,** *20,* 3174–3181. doi:10.3762/bjoc.20.262

Experimental procedures, product characterization, X-ray analysis and copies of NMR spectra

License and Terms: This is a supporting information file under the terms of the Creative Commons Attribution License (https://creativecommons.org/licenses/by/4.0). Please note that the reuse, redistribution and reproduction in particular requires that the author(s) and source are credited and that individual graphics may be subject to special legal provisions.
The license is subject to the *Beilstein Journal of Organic Chemistry* terms and conditions: (https://www.beilstein-journals.org/bjoc/terms)

Experimental section

1) General experimental methods

All experiments dealing with air- and moisture-sensitive compounds were conducted under an atmosphere of dry argon. The solvents, dry solvents and reagents were purchased from commercial sources and used without further purification. TLC was performed on silica gel, 60F-250 (0.26 mm thickness) plates. The plates were visualized with UV light (254 nm) or with a 3.5% solution of phosphomolybdic acid in ethanol or with a solution of $KMnO_4$ in water. Flash chromatography (FC) was performed on Merck 60 silica gel (230–400 mesh). Melting points were determined on a Büchi melting point apparatus. NMR spectra were measured on an Ultrafield Avance 300 (^{1}H, 300 MHz; ^{13}C, 75 MHz) and Bruker AMX 200 (^{1}H, 200 MHz; ^{19}F, 188 MHz) spectrometer. Unless otherwise stated, NMR data were obtained under ambient temperature conditions and $CDCl_3$, CD_3OD, or DMSO-d_6 were used as solvent. Chemical shifts δ are in ppm, and the following abbreviations are used: singlet (s), doublet (d), doublet doublet (dd), triplet (t), q (quartet), quintuplet (quint), sextuplet (sx), septuplet (sp), multiplet (m) and broad singlet (brs). High-resolution mass spectra were recorded on a MicrotofQ Bruker Daltonics.

2) General procedure:

General procedure [A]: synthesis of fluorinated hydrazones

The carbazate (1 equiv), and *p*-toluenesulfonic acid (0.05 equiv) are added to a solution of the fluorinated acetaldehyde ethyl hemiacetal (1.1 equiv) in toluene (0.1 M) in a round-bottomed flask. The reaction mixture is refluxed with a Dean–Stark apparatus to remove the water and the reaction is followed by ^{19}F NMR until disappearance of the starting material. Then, the solvent is removed under reduced pressure. The crude product is used directly in the next steps without further purification.

General procedure [B]: allylation reaction

The methyl or ethyl 2-(bromomethyl)acrylate (1.2 equiv) is added to a solution of hydrazone **3** (1 equiv) in tetrahydrofuran (5 mL/2 mmol). Then, an aqueous solution of saturated NH_4Cl (5 mL/2 mmol), and lastly zinc (1.2 equiv) are added to the reaction mixture. The mixture is stirred at room temperature until completion (^{19}F NMR or TLC), then diluted with water (5 mL) and extracted with diethyl ether (3 × 5 mL). The organic layer is washed with an aqueous solution of saturated NH_4Cl (5 mL), dried with $MgSO_4$, filtered and concentrated under vacuum. The residue is purified by flash chromatography (eluent: cyclohexane/ethyl acetate, ratio: 9:1–7:3).

General procedure [C]: oxidation of hydrazides

To a solution of the allylated compound **5** (1 equiv) in CH_2Cl_2 (5 mL) at 0 °C, I_2 or NBS (1.1 equiv) is added and lastly K_2CO_3 (1.1 equiv). The mixture is stirred at room temperature until completion. Then, the reaction mixture is diluted with 5 mL of CH_2Cl_2, washed with 5 mL of an aqueous saturated solution of $Na_2S_2O_3$ (only for the reaction with I_2), an aqueous saturated solution of NH_4Cl (2 × 5 mL), dried over Na_2SO_4, then filtered and concentrated under vacuum. If necessary, the residue is purified by flash chromatography (eluent: cyclohexane/ethyl acetate, ratio : 9:1–7:3).

General procedure [D]: cyclization procedure

Hydrazone **6** (1 equiv) is added to anhydrous DMF (6 mL/1 mmol) followed by K_2CO_3 (0.1 equiv). The reaction mixture is stirred overnight at room temperature. Once finished, it is diluted with water (5 mL) and extracted with diethyl ether (3 × 15 mL) for **7a**–**d** and ethyl acetate (3 × 15 mL) for **7e**,**f**. The organic layer is washed with an aqueous solution of saturated NH_4Cl (10 mL), dried with Na_2SO_4, filtered and concentrated under vacuum.

General procedure [E]: saponification and amino acid coupling procedure

To a solution of the ester **7** (1 equiv) in THF (1 mL/0.2 mmol of ester), is added an aqueous solution of $LiOH\cdot H_2O$ (2.5 equiv in 1 mL/0.2 mmol of ester) at 0 °C. The reaction mixture is stirred at 0 °C until completion (0.5–2 h, followed by TLC), then diluted with AcOEt (5 mL) and carefully acidified at 0 °C by an aqueous solution of HCl 0.1 N at 0 °C. The two layers are separated and the aqueous layer is extracted again with AcOEt (3 × 5mL). The organic phases are combined, washed with an aqueous solution of saturated NaCl (5 mL), dried over $MgSO_4$, filtered, and then evaporated to dryness. The corresponding acid is directly used without further purification in the next step. To a solution of the acid (1 equiv), EDC HCl (1.2 equiv), HOBt (1.2 equiv) and ʟ-Val-OMe·HCl (1.2 equiv) in dry DMF (1 mL/0.2 mmol), was added DIPEA (2.2 equiv) at 0 °C. The reaction mixture is stirred at room temperature overnight, then diluted with AcOEt (10 mL), washed

with water (2 × 3 mL), 10% $NaHCO_3$ aqueous solution (3 mL), 10% citric acid aqueous solution (3 mL) and saturated aqueous solution of NH_4Cl (3 mL). The organic phase is dried over anhydrous $MgSO_4$, filtered and concentrated under vacuum. Purification of the residue by silica gel column chromatography using cyclohexane/ethyl acetate ratio 7:3–5:5) as the eluent furnishes the tripeptide products.

3) Compounds characterization:

a) Hydrazones 3a–f:

tert-Butyl (E)-2-(2,2,2-trifluoroethylidene)hydrazine-1-carboxylate (3a)

The product **3a** is obtained following the general procedure (A) as a white powder (2.17 g, 93%).

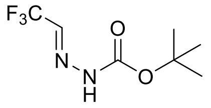

Chemical Formula: $C_7H_{11}F_3N_2O_2$
Exact Mass: 212,0773

^{1}H NMR (200 MHz, $CDCl_3$): δ = 9.05 (brs, 1H, NH), 7.64 (m, 1H, CH), 1.48 (s, 9H, $(CH_3)_3$)

^{19}F NMR (188 MHz, $CDCl_3$): δ = - 67.8 (d, $^3J_{H,F}$ = 3.0 Hz, 3F)

^{13}C NMR (75MHz, $CDCl_3$): δ= 152.9 (CO), 131.7 (q, $^2J_{C,F}$ = 39 Hz, CH), 120.4 (q, $^1J_{C,F}$ = 271 Hz, CF_3), 82.8 (Cq), 28.1(CH_3)

HRMS (ESI+TOF) m/z $C_7H_{11}F_3N_2O_2$ $[M+Na]^+$ calc. 235,0665, found 235.0672

Melting point: 117°C

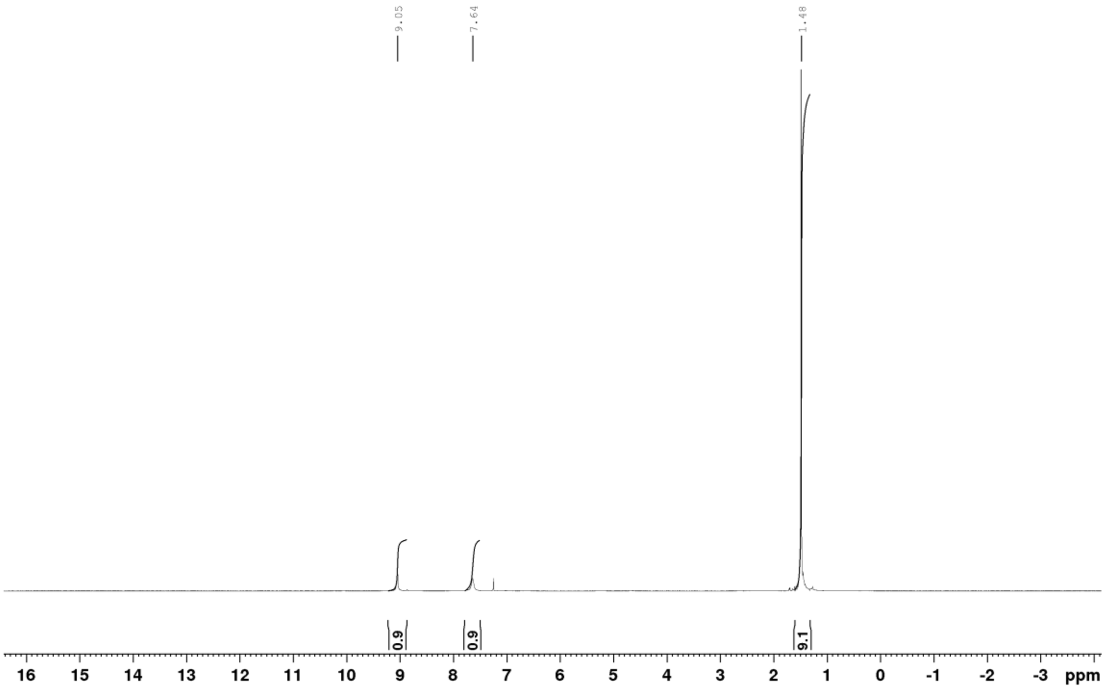

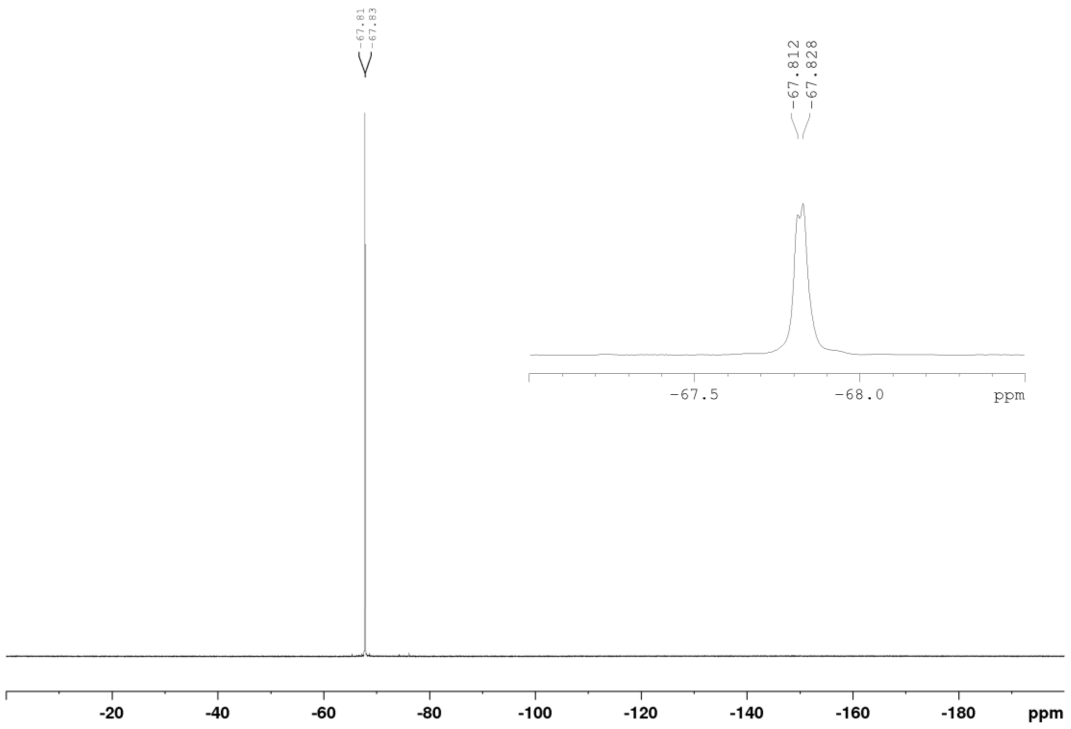
-67.81
-67.83
-67.812
-67.828
-67.5
-68.0
ppm
-20
-40
-60
-80
-100
-120
-140
-160
-180
ppm

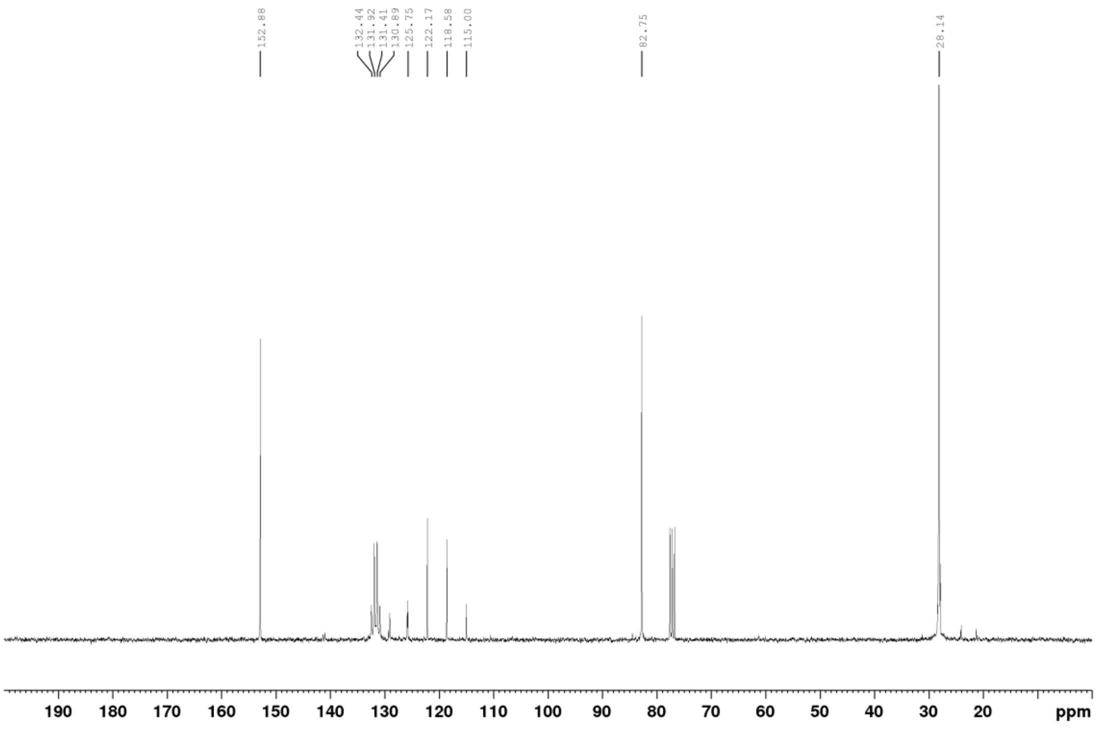
152.88
132.44
131.92
131.41
130.89
125.75
122.17
118.58
115.00
82.75
28.14
190
180
170
160
150
140
130
120
110
100
90
80
70
60
50
40
30
20
ppm

Benzyl (E)-2-(2,2,2-trifluoroethylidene)hydrazine-1-carboxylate (3b)

The product **3b** is obtained following the general procedure (A) as a white lightly yellow powder (2.91 g, 98%).

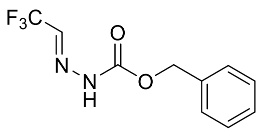

Chemical Formula: $C_{10}H_9F_3N_2O_2$
Exact Mass: 246,0616

^{1}H NMR (200 MHz, $CDCl_3$): δ = 8.99 (brs, 1H, NH), 7.59 (m, 1H, CH), 7.35 (m, 5H, Haro), 5.22 (s, 2H, CH_2).

^{19}F NMR (188 MHz, $CDCl_3$): δ = - 67.8 (brs, 3F).

^{13}C NMR (75MHz, $CDCl_3$): δ= 153.0 (CO), 135.0 (Caro), 132.7 (q, $^{2}J_{C,F}$ = 41 Hz, CH), 128.8, 128.7, 128.4 (CHaro), 119.0 (q, $^{1}J_{C,F}$ = 270 Hz, CF_3), 68.2 (CH_2).

HRMS (ESI+TOF) m/z $C_{10}H_9F_3N_2O_2$ $[M+Na]^+$ calc. 269,0508, found 269,0533

Melting point: 125°C

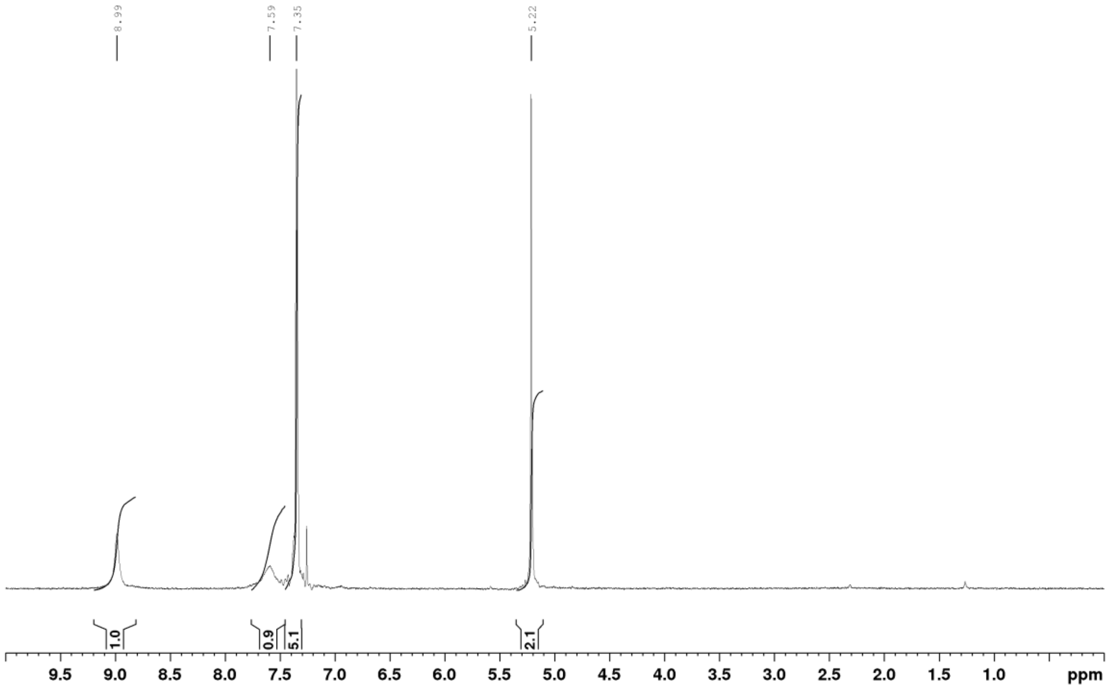

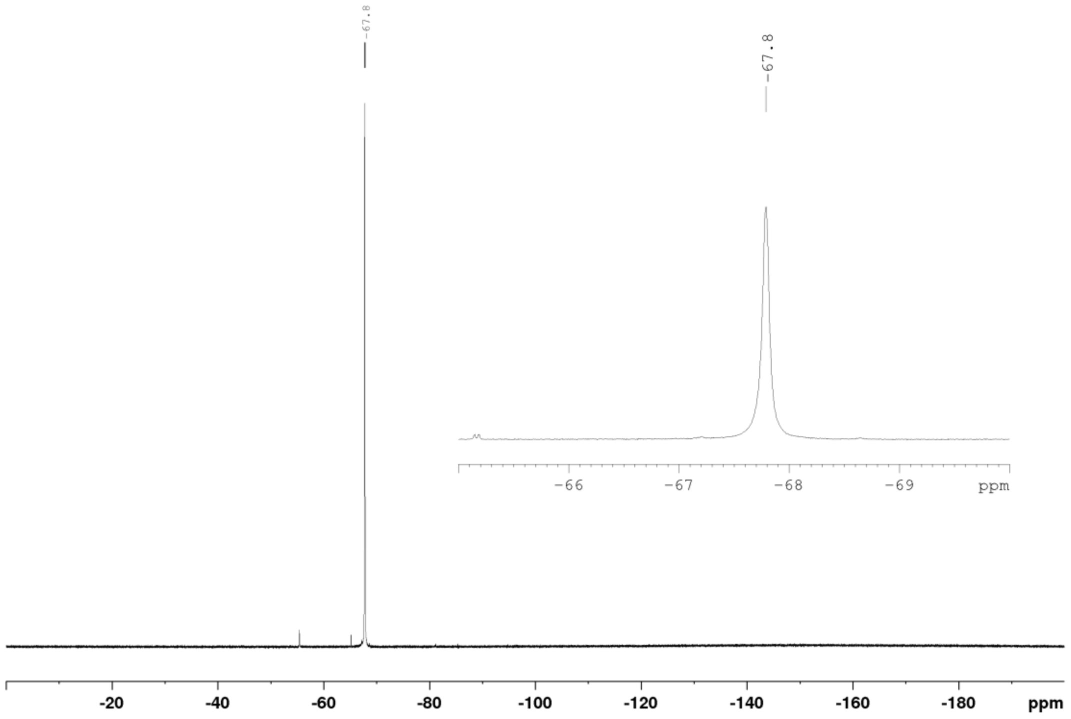
-67.8
-66
-67
-68
-69
ppm
-20
-40
-60
-80
-100
-120
-140
-160
-180
ppm

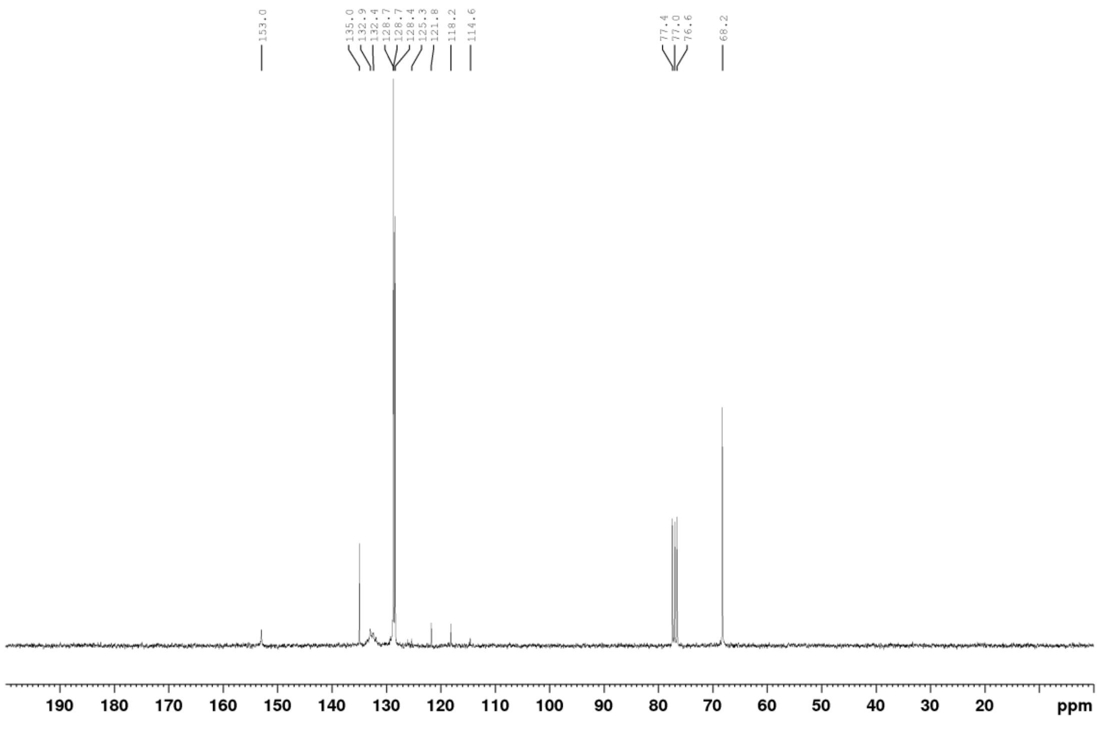
153.0
135.0
132.9
132.4
128.7
128.7
128.4
125.3
121.8
118.2
114.6
77.4
77.0
76.6
68.2
190
180
170
160
150
140
130
120
110
100
90
80
70
60
50
40
30
20
ppm

***tert*-Butyl (E)-2-(2,2-difluoroethylidene)hydrazine-1-carboxylate (3c)**

The product **3c** is obtained following the general procedure (A) as a white powder (1.67g, 92%).

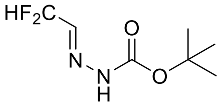

Chemical Formula: $C_7H_{12}F_2N_2O_2$
Exact Mass: 194,0867

^{1}H NMR (200 MHz, $CDCl_3$): δ = 8.43 (s, 1H, NH), 7.35-7.32 (m, 1H, CH), 6.14 (dt, 1H, $^2J_{H,F}$ = 54 Hz, $^3J_{H,H}$ = 6 Hz, CF_2H), 1.50 (s, 9H, $(CH_3)_3$).

^{19}F NMR (188 MHz, $CDCl_3$): δ = - 116.1 (dd, 2F, $^2J_{H,F}$ = 54 Hz, $^3J_{H,F}$ = 2.5 Hz)

^{13}C NMR (75 MHz, $CDCl_3$): δ = 153.6 (CO), 136.3 (t, $^2J_{C,F}$ = 34 Hz, CH), 113.3 (t, $^1J_{C,F}$ = 233 Hz, CF_2H), 82.7 (Cq), 28.1 (CH_3).

HRMS (ESI+TOF) m/z $C_7H_{12}N_2O_2F_2$ $[M+Na]^+$ calc. 217.0667, found 217.0765.

Melting point: 138°C

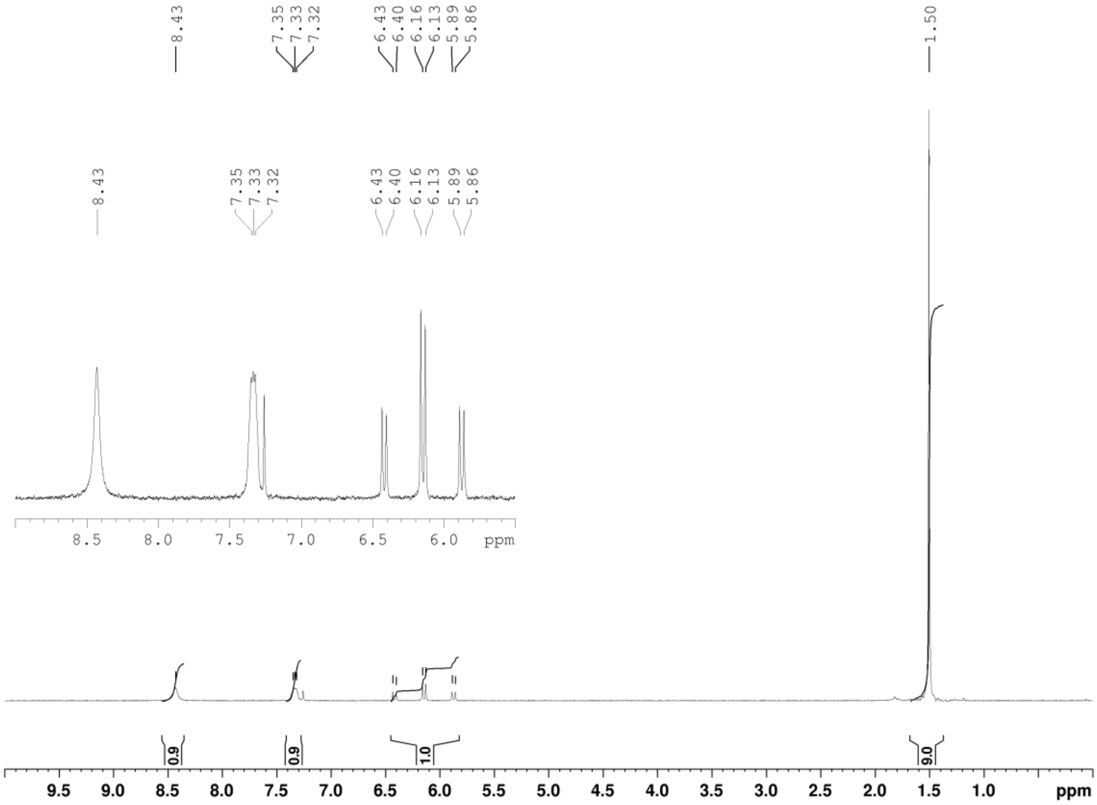

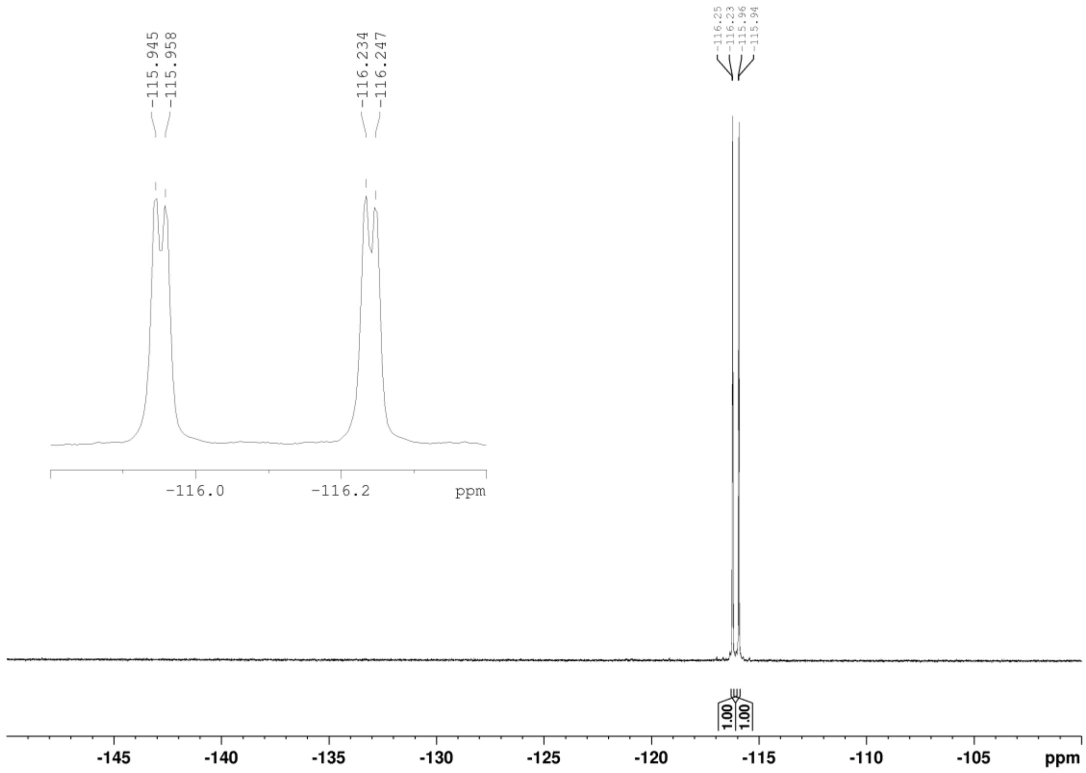
-116.25
-116.23
-115.96
-115.94
-115.945
-115.958
-116.234
-116.247
-116.0
-116.2
ppm
1.00
1.00
-145
-140
-135
-130
-125
-120
-115
-110
-105
ppm

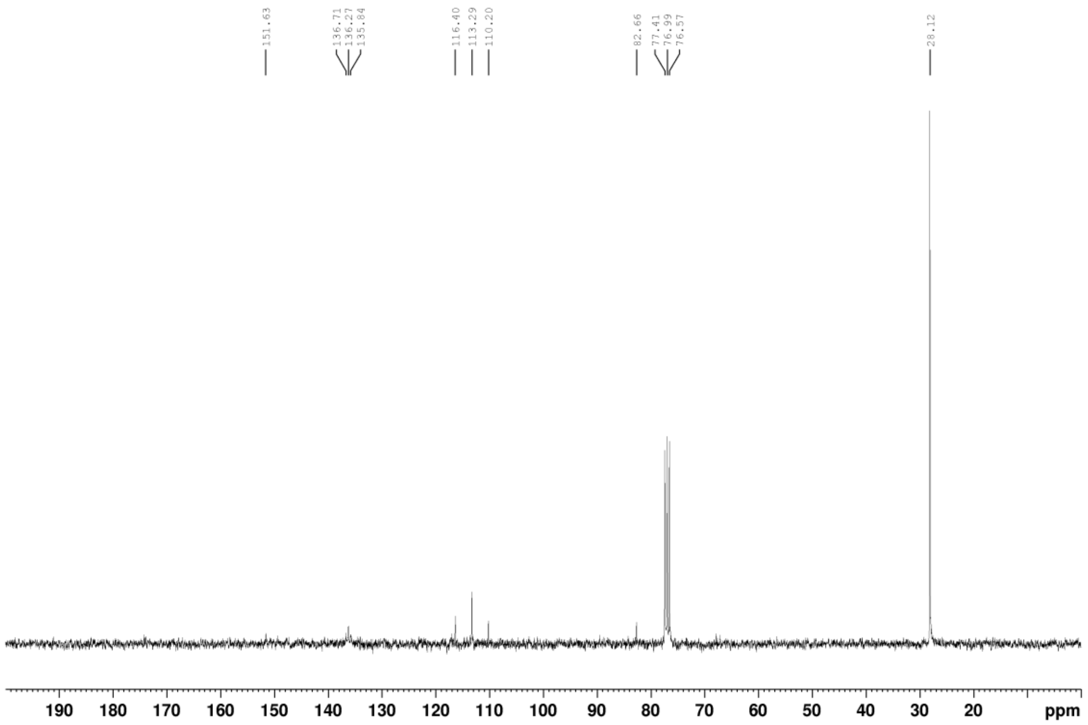
151.63
136.71
136.27
135.84
116.40
113.29
110.20
82.66
77.41
76.99
76.57
28.12
190
180
170
160
150
140
130
120
110
100
90
80
70
60
50
40
30
20
ppm

Benzyl (E)-2-(2,2-difluoroethylidene)hydrazine-1-carboxylate (3d)

The product **3d** is obtained following the general procedure (**A**) as a white powder (2.68 g, 99%).

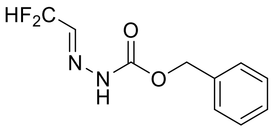

Chemical Formula: $C_{10}H_{10}F_2N_2O_2$
Exact Mass: 228,0710

^{1}H NMR (200 MHz, $CDCl_3$): δ = 8.84 (s, 1H, NH), 7.37 (m, 5H, Haro), 7.33-7.25 (m, 1H, CH), 6.15 (dt, 1H, $^{2}J_{H,F}$ = 54 Hz, $^{3}J_{H,H}$ = 6 Hz, CF_2H), 5.24 (s, 2H, CH_2).

^{19}F NMR (188 MHz, $CDCl_3$): δ = - 116.3 (dd, $^{2}J_{H,F}$ = 54 Hz, $^{3}J_{H,F}$ = 1.9 Hz, 2F)

^{13}C NMR (75 MHz, $CDCl_3$): δ = 153.2 (CO), 137.7 (t, $^{2}J_{C,F}$ = 33 Hz, CH), 135.1 (Caro), 128.7, 128.4, 127.2 (CHaro), 112.9 (t, $^{1}J_{C,F}$ = 234 Hz, CF_2H), 68.1 (CH_2).

HRMS (ESI+TOF) m/z $C_{10}H_{10}F_2N_2O_2$ $[M+Na]^+$ calc. 251.0501, found 251.0578.

Melting point: 152°C

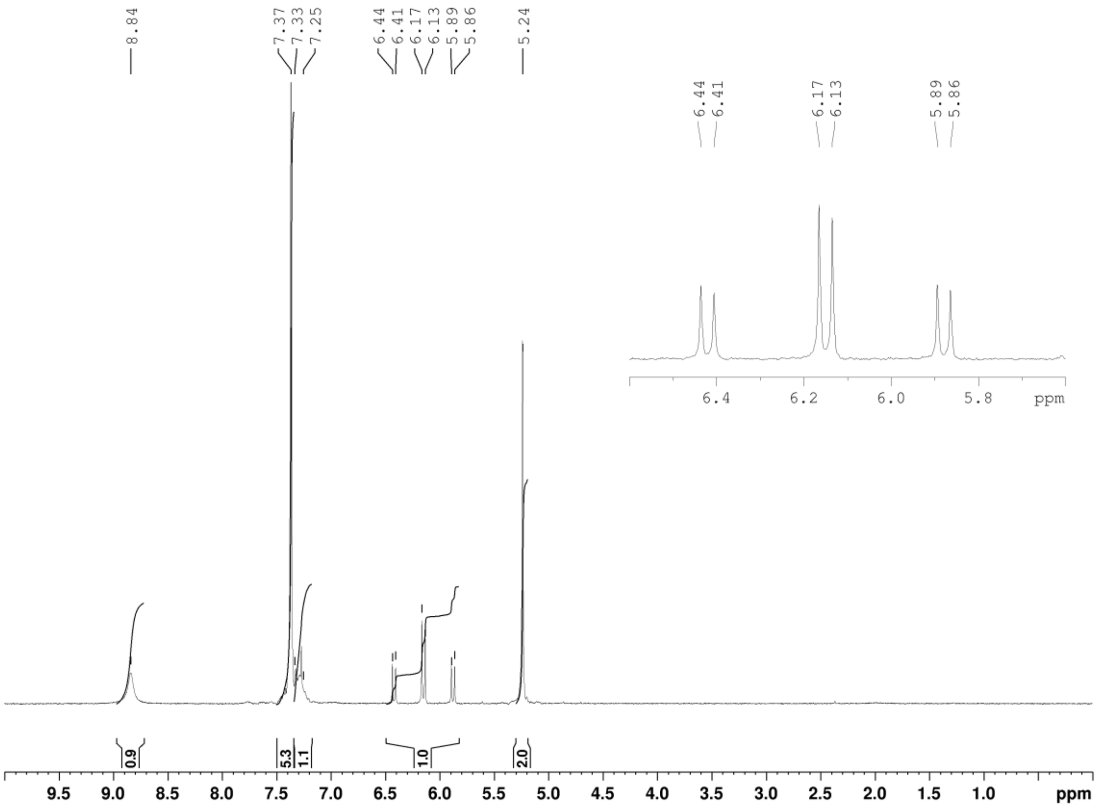

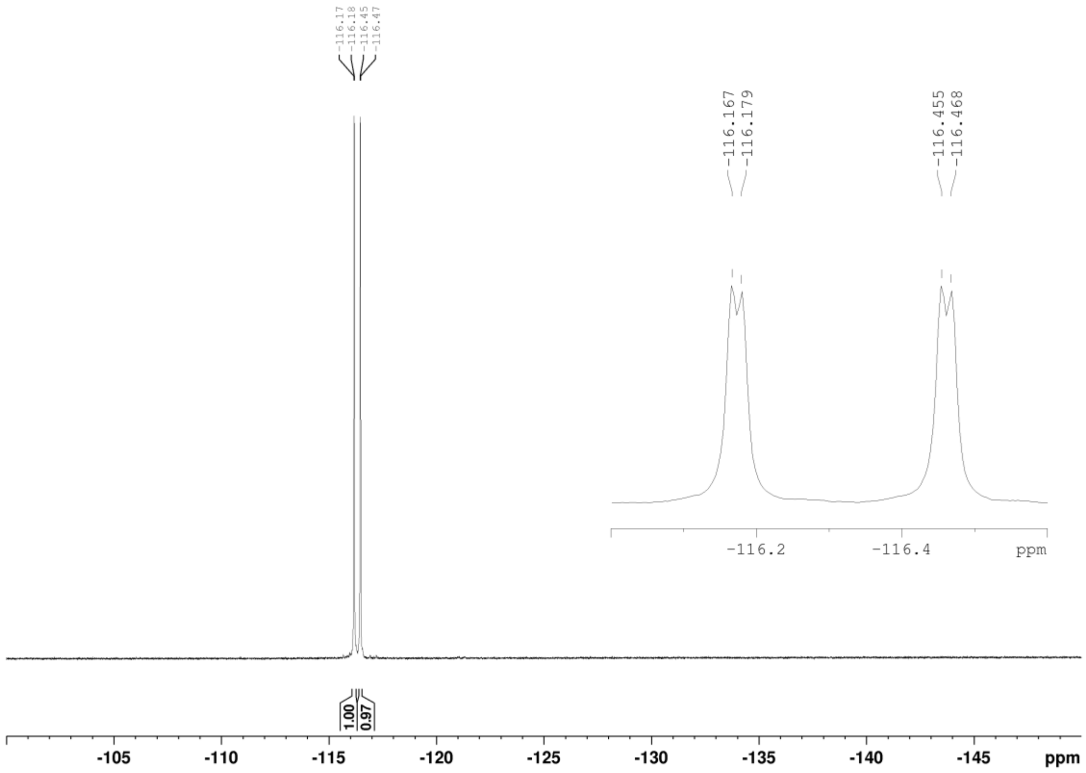
-116.17
-116.18
-116.45
-116.47
-116.167
-116.179
-116.455
-116.468
-116.2
-116.4
ppm
1.00
0.97
-105
-110
-115
-120
-125
-130
-135
-140
-145
ppm

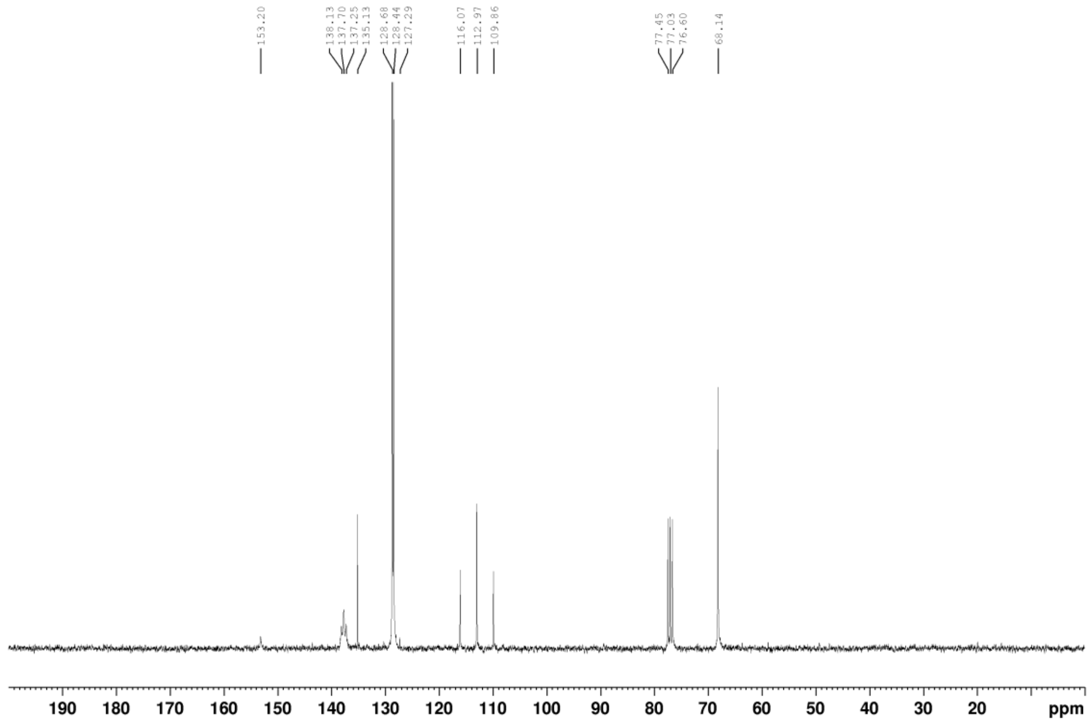
153.20
138.13
137.70
137.25
135.13
128.68
128.44
127.29
116.07
112.97
109.86
77.45
77.03
76.60
68.14
190
180
170
160
150
140
130
120
110
100
90
80
70
60
50
40
30
20
ppm

Benzyl (*S*)-(1-hydrazineyl-1-oxo-3-phenylpropan-2-yl)carbamate

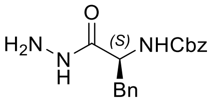

Chemical Formula: $C_{17}H_{19}N_3O_3$
Exact Mass: 313,1426

To a solution of ʟ-Z-PheOH (2 g, 1 equiv), $BocNHNH_2$ (0.87 g, 1 equiv), EDC·HCl (1.39 g, 1.1 equiv), HOBt (1.11 g, 1.1 equiv) in dry CH_2Cl_2 (15 mL), is added diisopropylethylamine (1.70 g, 2 equiv) at 0 °C. The reaction mixture is stirred overnight at room temperature, then diluted with 15 mL of CH_2Cl_2 and washed with $NaHCO_3$ 10% aqueous solution, citric acid 10% aqueous solution and NaCl saturated aqueous solution. The organic layer is dried over $MgSO_4$, filtered and evaporated to dryness. Then, the residue (2.90 g, 1 equiv) is diluted in the minimum of CH_2Cl_2 and a solution of HCl 4 N in dioxane (11 mL, 5.5 equiv) is added at 0 °C and stirred at room temperature until disappearance of the starting material (followed by TLC). After evaporation of the solvent, the residue is suspended in CH_2Cl_2 (20 mL) and basified with an aqueous saturated solution of Na_2CO_3. After separation of the organic layer, the aqueous layer is extracted again with CH_2Cl_2 (2 × 15 mL). The three organic layers are combined and dried over $MgSO_4$, filtered and evaporated to dryness. The Z-ʟ-phenylalanine hydrazide is obtained as a white powder (2.35 g, 85% overall yield).

^{1}H NMR (300 MHz, DMSO-d$_6$): δ = 9.20 (s, 1H, NH), 7.48 (d, $^{3}J_{H,H}$ = 8.7 Hz, 1H, NHCbz), 7.33-7.24 (m, 10H, Haro), 4.94 (s, 2H, CH_2Cbz), 4.19 (ddd, $^{3}J_{H,H}$ = 8.7 Hz, $^{3}J_{H,H}$ = 4.6 Hz, $^{3}J_{H,H}$ = 10.2 Hz, 1H, CH), 2.93 (dd, $^{3}J_{H,H}$ = 4.6 Hz, $^{2}J_{H,H}$ = 13.7 Hz, 1H, CH_2Ph), 2.77 (dd, $^{3}J_{H,H}$ = 10.3 Hz, $^{2}J_{H,H}$ = 13.7 Hz, 1H, CH_2Ph).

^{13}C NMR (75 MHz, DMSO-d$_6$): δ = 170.6 (CO), 155.7 (COBn), 138.0, 137.0 (Caro), 129.1, 128.2, 128.0, 127.6, 127.4, 126.2 (CHaro), 65.2 (CH_2Cbz), 54.9 (CH), 37.7 (CH_2Ph).

HRMS (ESI+TOF) m/z $C_{17}H_{19}N_3O_3$ $[M+H]^+$ calc. 314.1499, found 314.1503.

Melting point: 155°C

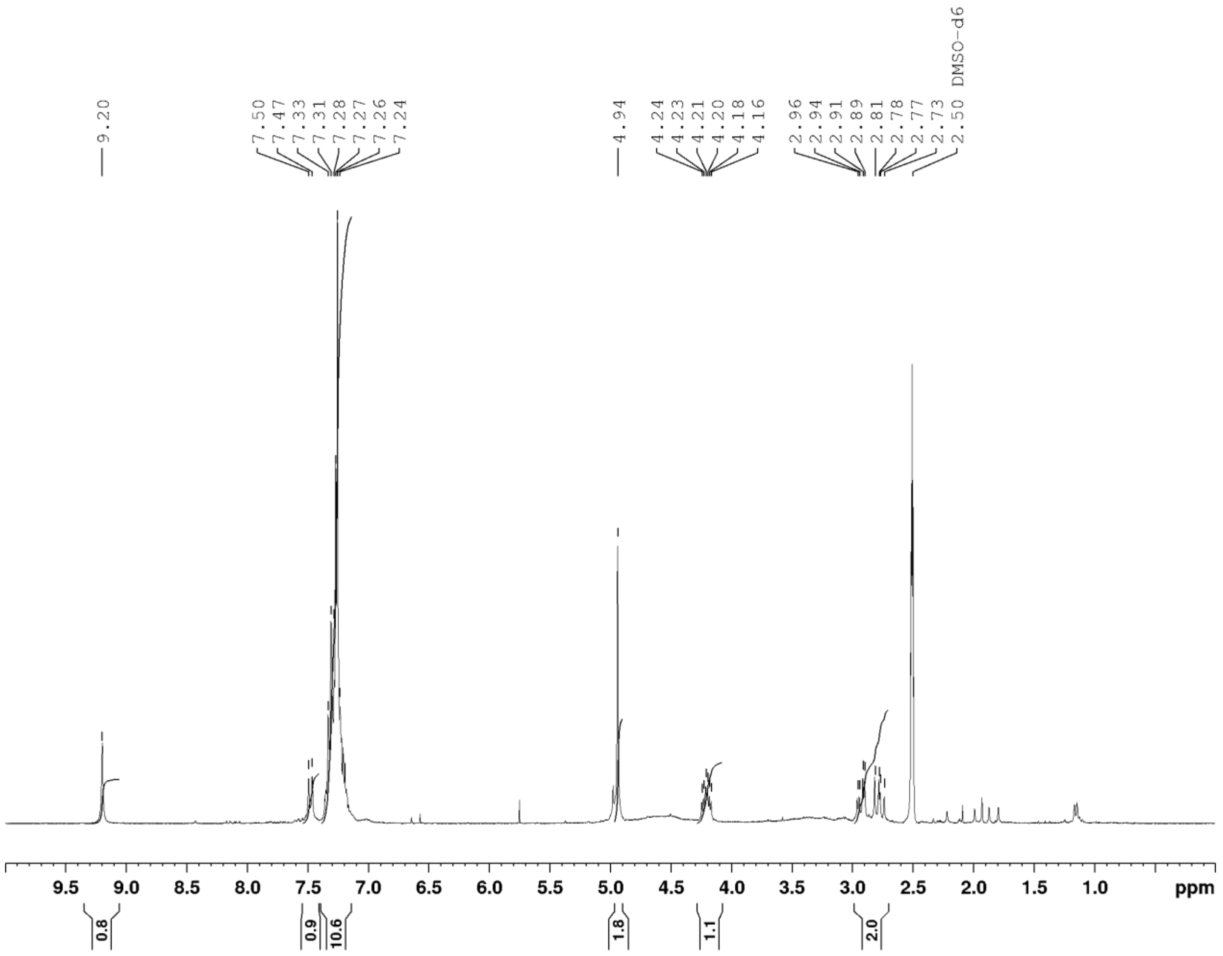

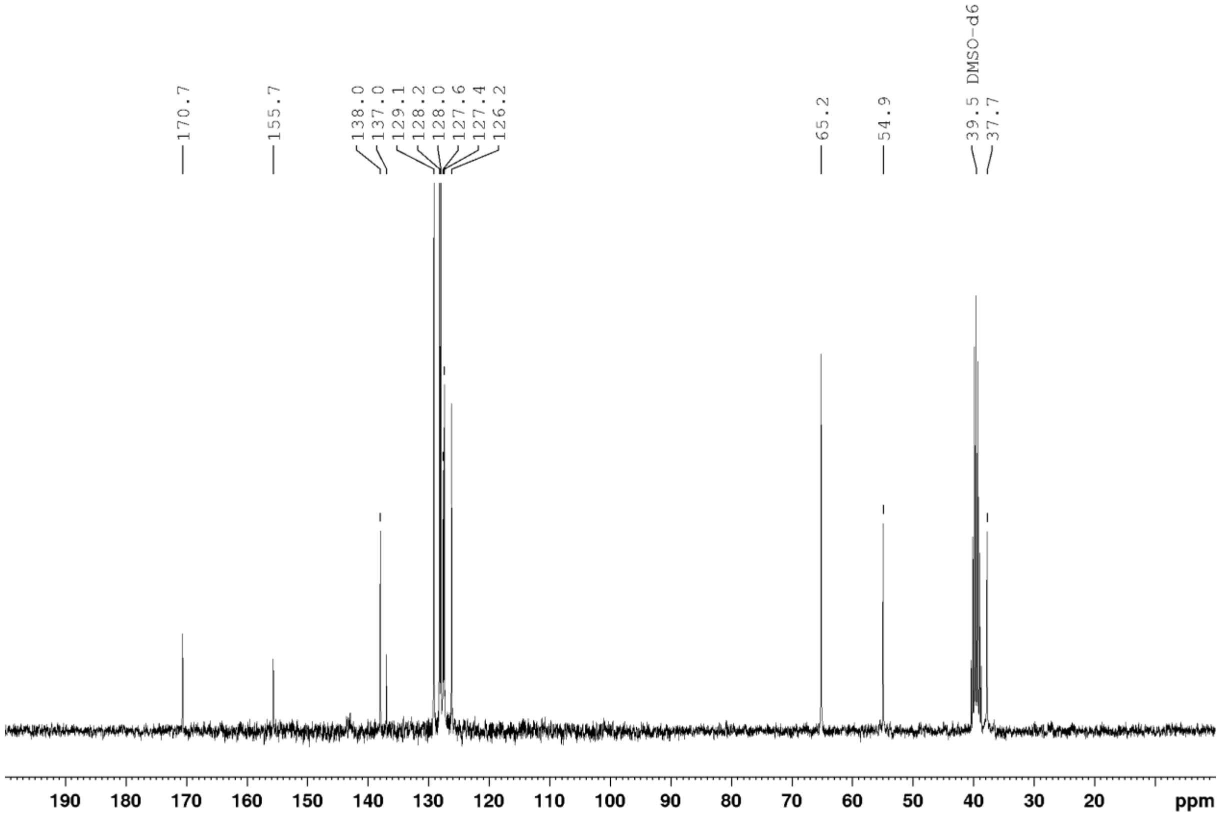
170.7
155.7
138.0
137.0
129.1
128.2
128.0
127.6
127.4
126.2
65.2
54.9
39.5 DMSO-d6
37.7
190 180 170 160 150 140 130 120 110 100 90 80 70 60 50 40 30 20 ppm

Benzyl (S,E)-(1-oxo-3-phenyl-1-(2-(2,2,2-trifluoroethylidene)hydrazineyl)propan-2-yl)carbamate (3e)

The product **3e** is obtained following the general procedure (A) as a mixture of two conformers (ratio: 51/49), (0.745 g, 91%).

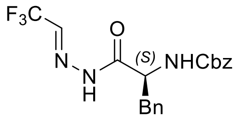

Chemical Formula: $C_{19}H_{18}F_3N_3O_3$
Exact Mass: 393,1300

Conformer 1:

<u>^{1}H NMR</u> (300 MHz, DMSO-d$_6$): δ = 12.19 (s, 1H, NH), 7.97 (d, $^3J_{H,H}$=3.45 , 1H, NHCbz), 7.87 (d, $^3J_{H,H}$ =7.89 Hz, 1H, CH), 7.39-7.28 (m, 10H, Haro), 5.02 (s, 2H, CH_2Cbz), 4.35 (m, 1H, CH), 3.10-2.75 (m, 2H, CH_2Ph).

^{19}F NMR (188 MHz, DMSO-d$_6$): δ = - 66.2 (d, $^3J_{H,F}$ = 2.55 Hz, 3F).

^{13}C NMR (75 MHz, DMSO-d$_6$): δ = 169.5 (CO), 156.0 (COCbz), 137.8, 136.8 (Caro), 134.1 (q, $^2J_{C,F}$ = 38 Hz, CH-CF_3), 129.2, 128.9, 128.3, 128.1, 127.6, 126.5, 125.5 (CHaro), 120.3 (q, $^1J_{C,F}$ = 266 Hz, CF_3), 65.4 (CH_2Cbz), 55.6 (CH), 36.7, (CH_2Ph).

Conformer 2:

^{1}H NMR (300 MHz, DMSO-d$_6$): δ = 11.99 (s, 1H, NH), 7.79 (d, $^3J_{H,H}$ =8.19 Hz, 1H, NHCbz), 7.59 (d, $^3J_{H,H}$ = 3.57 Hz, 1H, CH), 7.39-7.28 (m, 10H, Haro), 5.02 (s, 2H, CH_2Cbz), 4.96 (m, 1H, CH), 3.10-2.75 (m, 2H, CH_2Ph).

^{19}F NMR (188 MHz, DMSO-d$_6$): δ = -66.3 (d, 3F, $^3J_{H,F}$ = 2.60 Hz, 3F).

^{13}C NMR (75 MHz, DMSO-d$_6$): δ = 169.5 (CO), 156.0 (COCbz), 137.4, 136.8 (Caro), 129.9 (q, $^2J_{C,F}$ = 38 Hz, CH-CF_3), 129.2, 128.9, 128.3, 128.1, 127.6, 126.5, 125.5 (CHaro), 120.1 (q, $^1J_{C,F}$ = 266 Hz, CF_3), 65.3 (CH_2Cbz), 53.5 (CH), 36.2 (CH_2Ph).

HRMS (ESI+TOF) m/z $C_{19}H_{18}F_3N_3O_3$ $[M+Na]^+$ calc. 416.1192, found 416.1187.

Melting point: 176°C

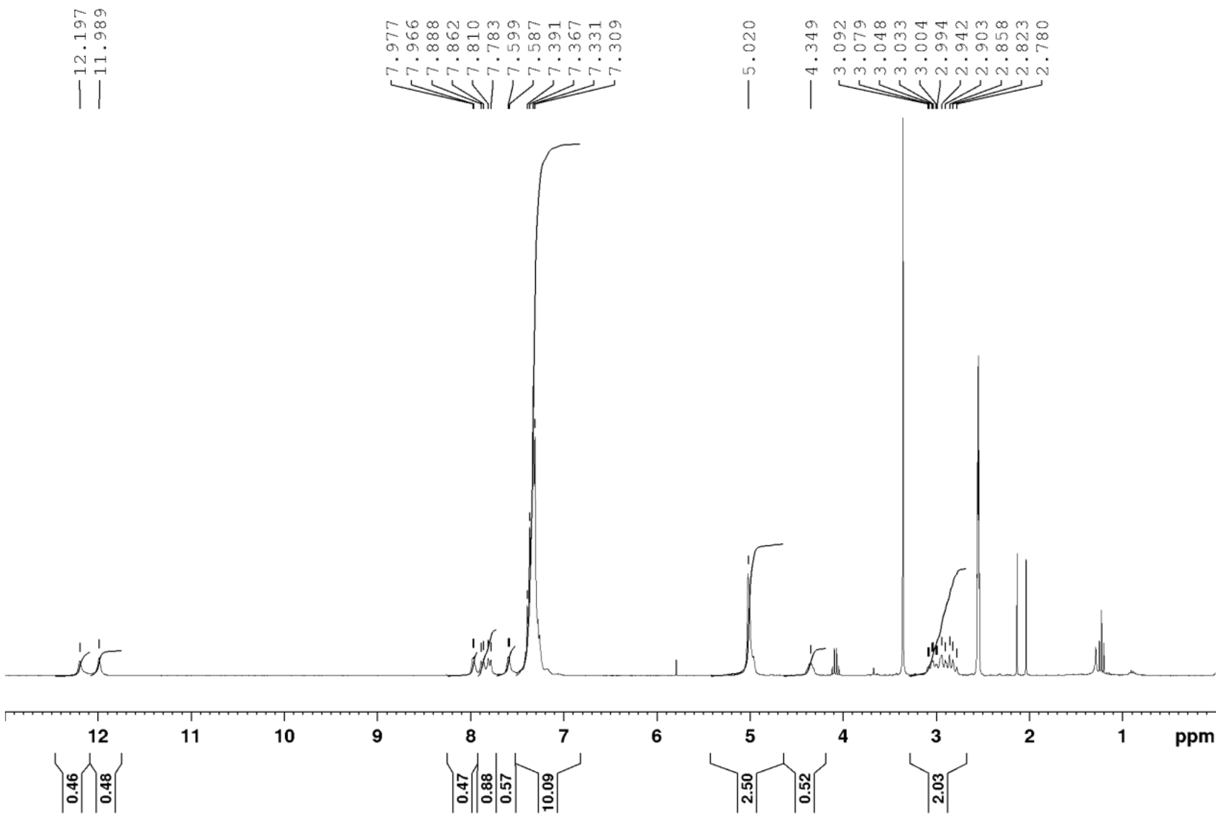

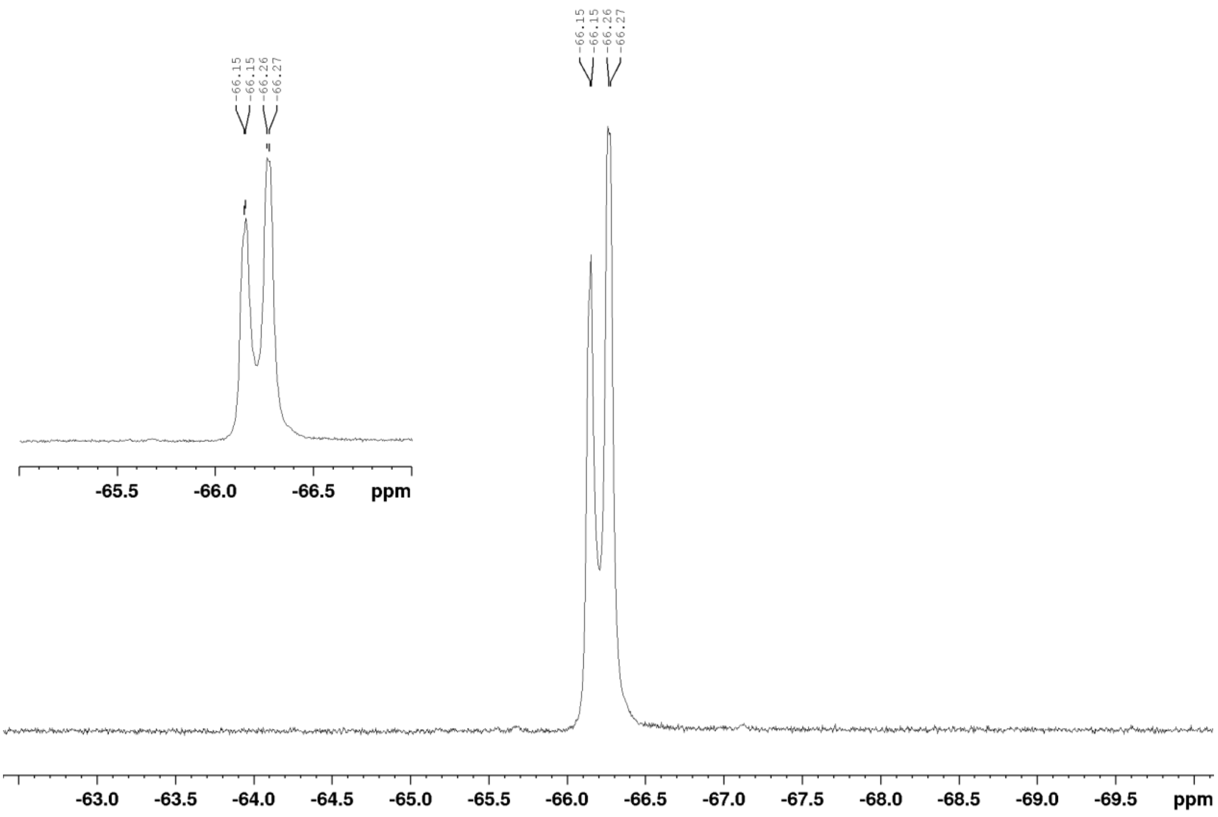
-66.15
-66.15
-66.26
-66.27
-65.5
-66.0
-66.5
ppm
-63.0
-63.5
-64.0
-64.5
-65.0
-65.5
-66.0
-66.5
-67.0
-67.5
-68.0
-68.5
-69.0
-69.5
ppm

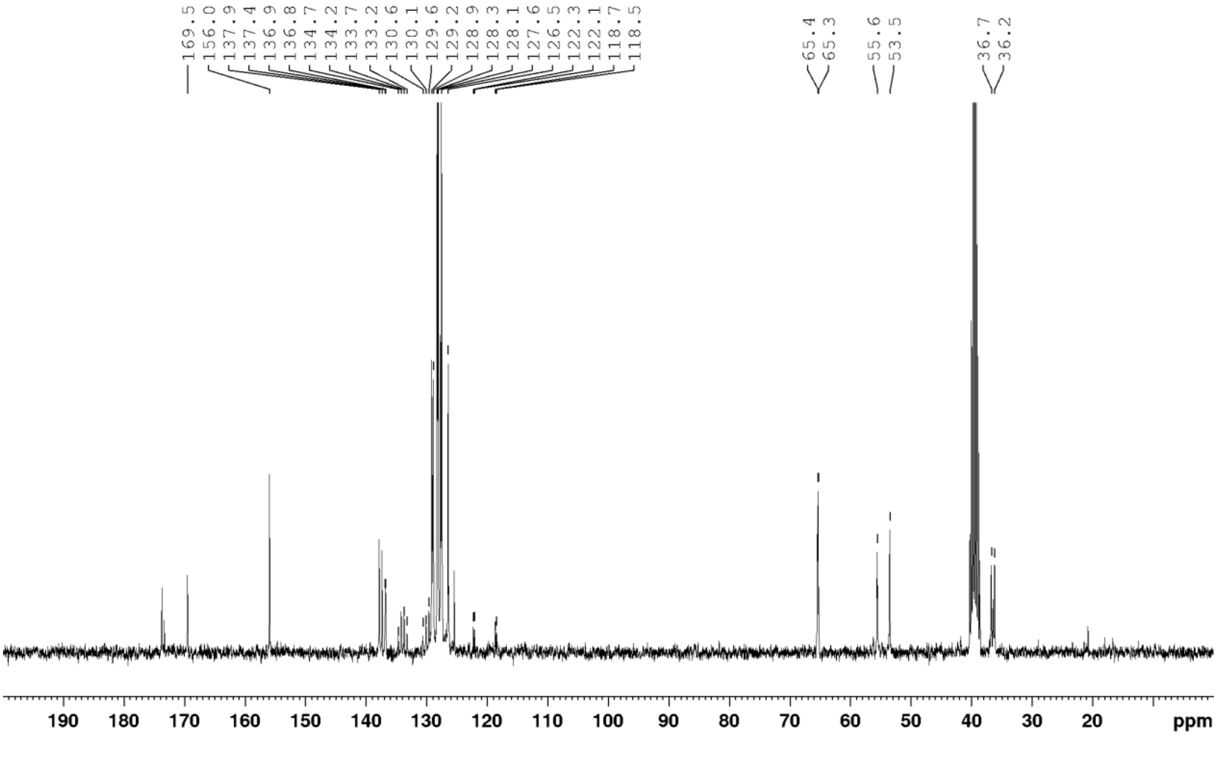
169.5
156.0
137.9
137.4
136.9
136.8
134.7
134.2
133.7
133.2
130.6
130.1
129.6
129.2
128.9
128.3
128.1
127.6
126.5
122.3
122.1
118.7
118.5
65.4
65.3
55.6
53.5
36.7
36.2
190
180
170
160
150
140
130
120
110
100
90
80
70
60
50
40
30
20
ppm

2D 1H-1H NOESY experiments of 3e

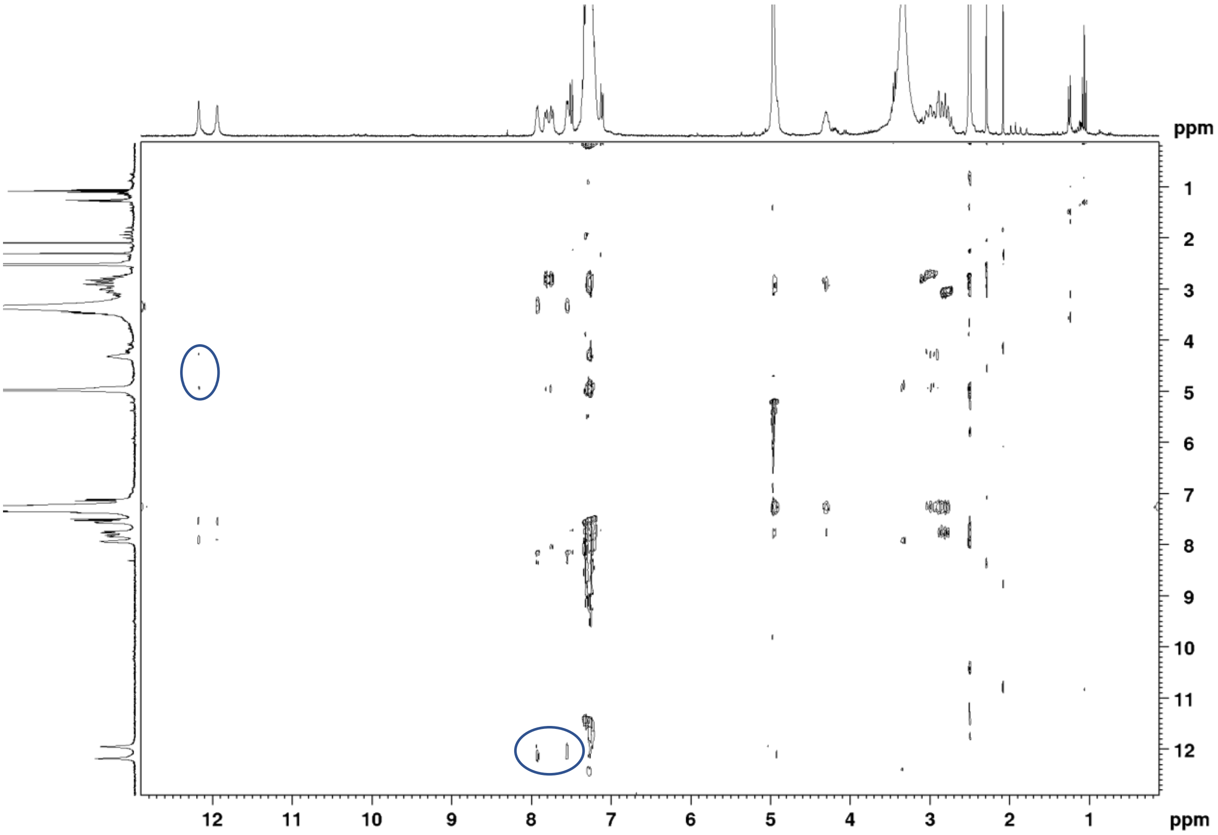

Benzyl (S,E)-(1-oxo-3-phenyl-1-(2-(2,2-difluoroethylidene)hydrazineyl)propan-2-yl)carbamate (3f)

The product **3f** is obtained following the general procedure (A) as a mixture of two conformers (ratio: 51/49) (1.15 g, 96%).

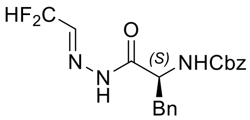

Conformer 1:

^{1}H NMR (200 MHz, DMSO-d$_6$): δ = 11.91 (s, 1H, NH), 7.80 (d, $^3J_{H,H}$ =7.9 Hz, 1H, NHCbz), 7.72 (d, $^3J_{H,H}$ = 4.7 Hz, 1H, CH), 7.38-7.26 (m, 10H, Haro), 6.44 (td, $^2J_{H,F}$ = 81 Hz, $^3J_{H,H}$ = 4.9 Hz, 1H, CF_2H), 4.99 (s, 2H, CH_2Cbz), 4.29 (m, 1H, CH), 3.05-2.74 (m, 2H, CH_2Ph).

^{19}F NMR (188 MHz, DMSO-d$_6$): δ = -116.59 (dd, $^2J_{H,F}$ = 54 Hz, $^3J_{H,F}$ = 2.7 Hz, 2F)

^{13}C NMR (75 MHz, DMSO-d$_6$): δ = 169.0 (CO), 155.9 (COCbz), 140.1 (t, $^2J_{C,F}$ = 31 Hz, CH-CF_2H), 137.8, 136.9 (Caro), 129.1, 128.2, 128.1, 127.7, 127.5, 126.4 (CHaro), 113.3 (t, $^1J_{C,F}$ = 233 Hz, CF_2H), 65.4 (CH_2Cbz), 55.5 (CH), 36.9, 36.3 (CH_2Ph).

Conformer 2:

^{1}H NMR (200 MHz, DMSO-d$_6$): δ = 11.67 (s, 1H, NH), 7.68 (d, $^3J_{H,H}$ = 9.1 Hz, 1H, NHCbz), 7.42 (d, $^3J_{H,H}$ = 4.4 Hz, 1H, CH), 7.38-7.26 (m, 10H, Haro), 6.51 (td, $^2J_{H,F}$ = 54 Hz, $^3J_{H,H}$ = 5 Hz, 1H, CF_2H), 4.99 (s, 2H, CH_2Cbz), 4.93 (m, 1 H, CH), 3.05-2.74 (m, 2H, CH_2Ph)

^{19}F NMR (188 MHz, DMSO-d$_6$): δ = -116.61 (dd, $^2J_{H,F}$ = 81 Hz, $^3J_{H,F}$ = 3.6 Hz, 2F)

^{13}C NMR (75 MHz, DMSO-d$_6$): δ = 169.0 (CO), 155.9 (COCbz), 137.4, 136.8 (Caro), 136.3 (t, $^2J_{C,F}$ = 31 Hz, C*H*CF_2H), 129.1, 128.2, 128.1, 127.7, 127.5, 126.4 (CHaro), 112.9 (t, $^1J_{C,F}$ = 233 Hz, CF_2H), 65.3 (CH_2Cbz), 53.3 (CH), 36.3 (CH_2Ph).

HRMS (ESI+TOF) m/z $C_{19}H_{19}F_2N_3O_3$ $[M+Na]^+$ calc. 398.1286, found 398.1281.

Melting point: 193°C

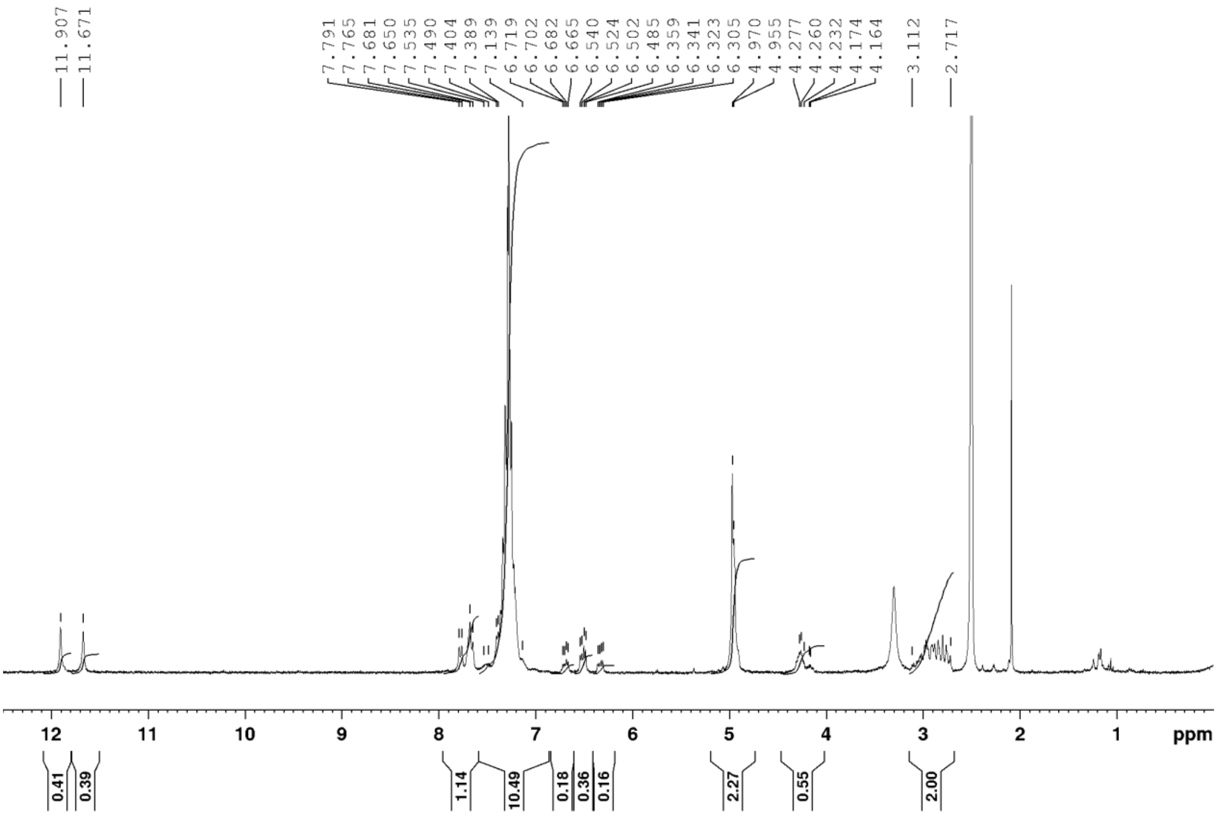

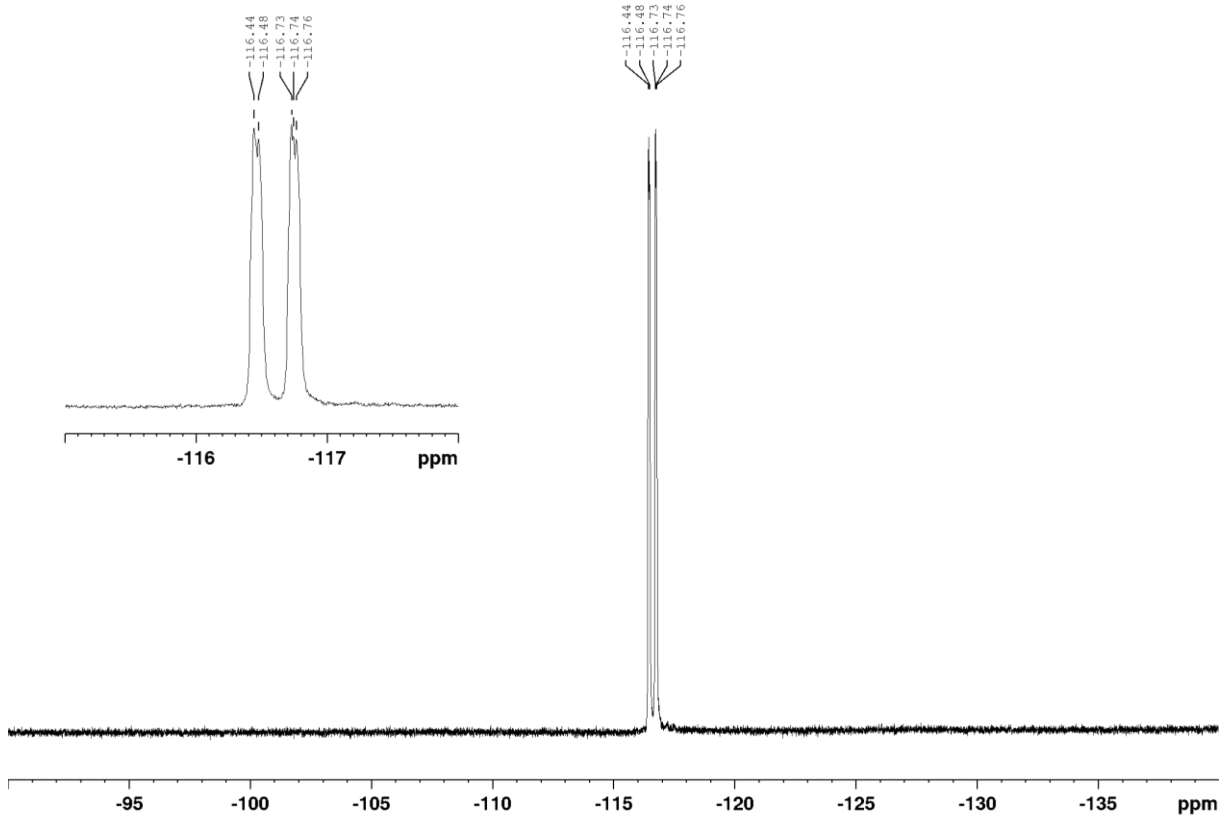
-116.44
-116.48
-116.73
-116.74
-116.76
-116
-117
ppm
-95
-100
-105
-110
-115
-120
-125
-130
-135
ppm

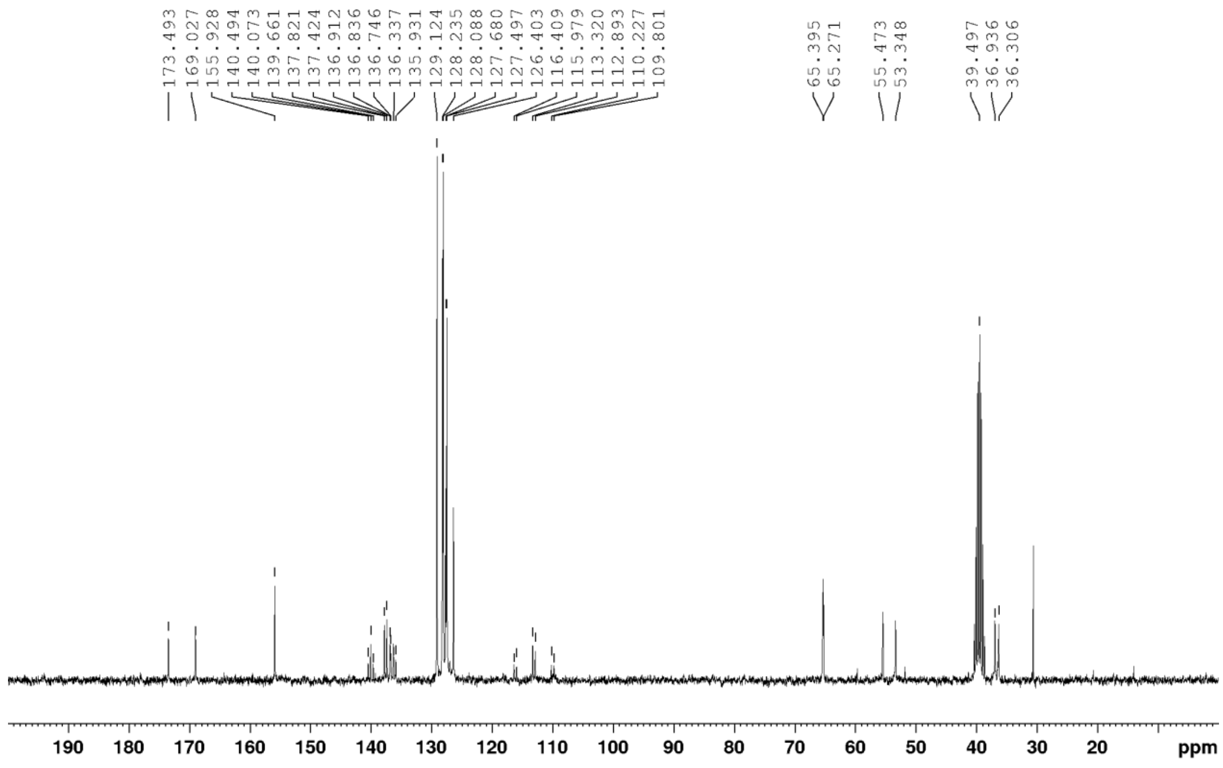
173.493
169.027
155.928
140.494
140.073
139.661
137.821
137.424
136.912
136.836
136.746
136.337
135.931
129.124
128.235
128.088
127.680
127.497
126.403
116.409
115.979
113.320
112.893
110.227
109.801
65.395
65.271
55.473
53.348
39.497
36.936
36.306
190
180
170
160
150
140
130
120
110
100
90
80
70
60
50
40
30
20
ppm

2D ^{1}H-^{1}H NOESY experiment of 3f

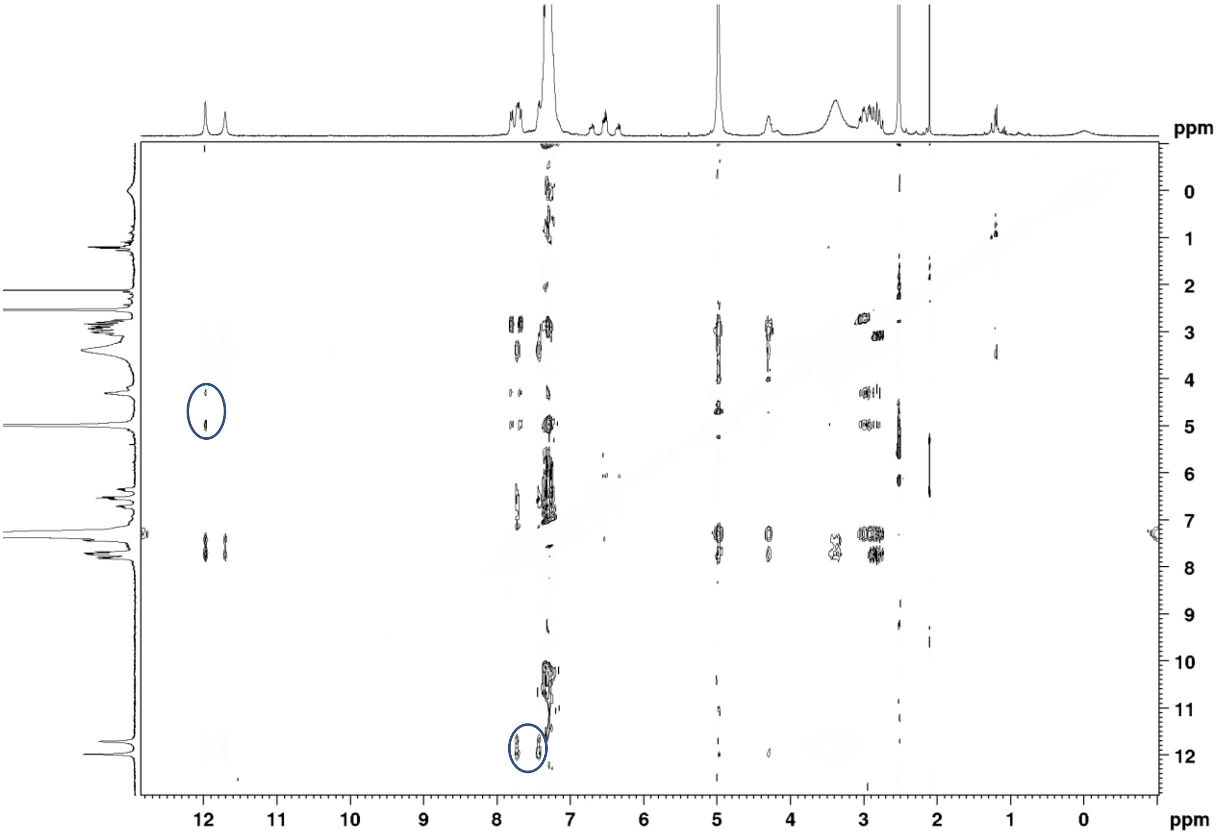

b) Allylated compounds 5a–f:

***tert*-Butyl 2-(1,1,1-trifluoro-4-(methoxycarbonyl)pent-4-en-2-yl)hydrazine-1-carboxylate (5a)**

The product **5a** is obtained following the general procedure (**B**) as a yellow oil (0.531g, 73%).

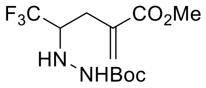

Chemical Formula: $C_{12}H_{19}F_3N_2O_4$
Exact Mass: 312,1297

^{1}H NMR (200 MHz, $CDCl_3$): δ = 6.37 (s, 1H, =CH_2), 6.21 (brs, 1H, NHBoc), 5.81 (s, 1H, =CH_2), 4.22 (brs, 1H, NH), 3.79 (s, 3H, OCH_3), 3.55-3.79 (m, 1H, CH), 2.76 (dd, $^3J_{H,H}$ = 3.5 Hz, $^1J_{H,H}$ = 14.6 Hz, 1H, CH_2), 2.46 (dd, $^3J_{H,H}$ = 9,0 Hz, $^1J_{H,H}$ = 14.6 Hz, 1H, CH_2), 1,42 (s, 9H, $(CH_3)_3$).

^{19}F NMR (188 MHz, $CDCl_3$): δ = - 75.4 (brs, 3F)

^{13}C NMR (75 MHz, $CDCl_3$): δ = 166.7 (CO), 156.0 (COCbz), 135.1 (=Cq), 128.9 (=CH_2), 125.8 (q, $^1J_{H,H}$ = 282 Hz, CF_3), 80.6 (Cq), 60.3 (q, $^2J_{H,H}$ = 27 Hz, CH-CF_3), 51.8 (OCH_3), 29.8 (CH_2), 28.0 (CH_3).

HRMS (ESI+TOF) m/z $C_{12}H_{19}F_3N_2O_4$ $[M+Na]^+$ calc. 335.1189, found. 335.1156

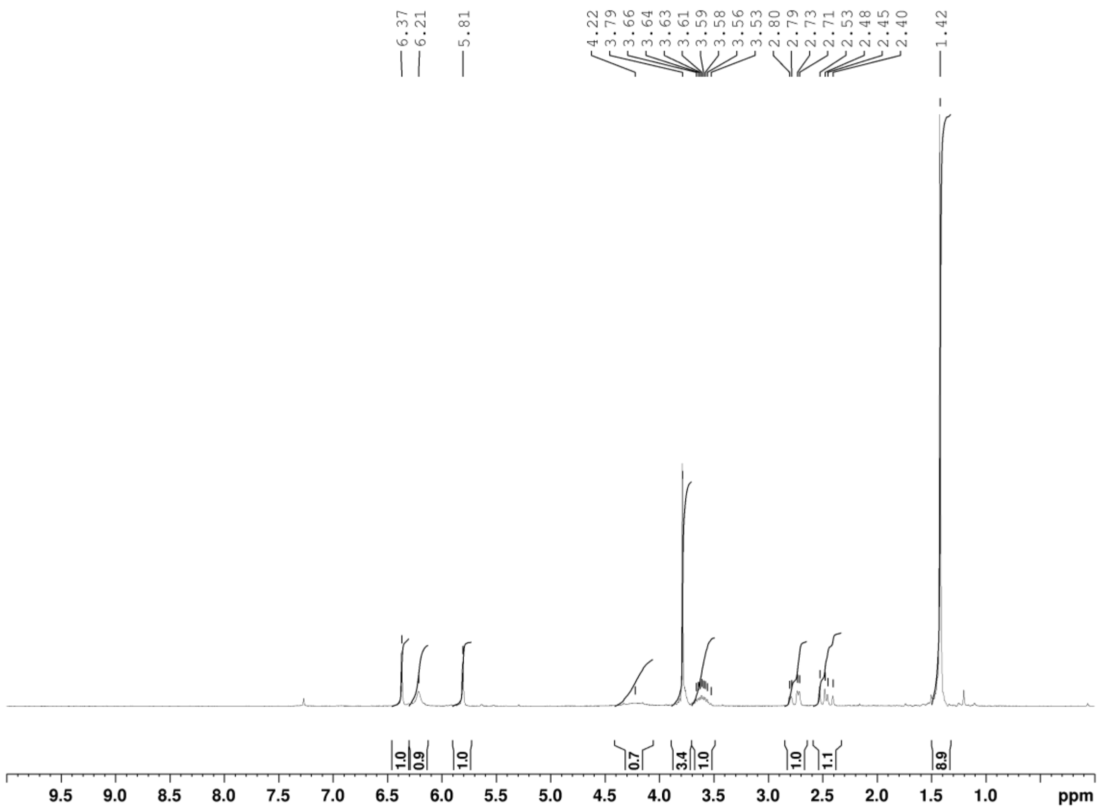

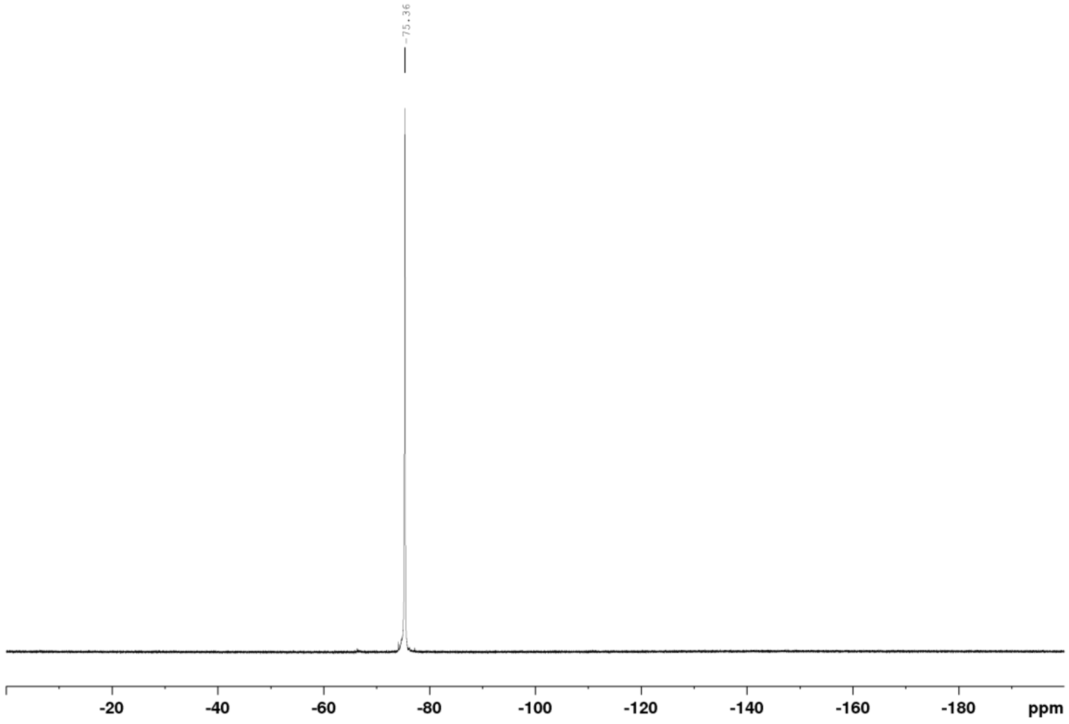

-75.36
-20
-40
-60
-80
-100
-120
-140
-160
-180
ppm

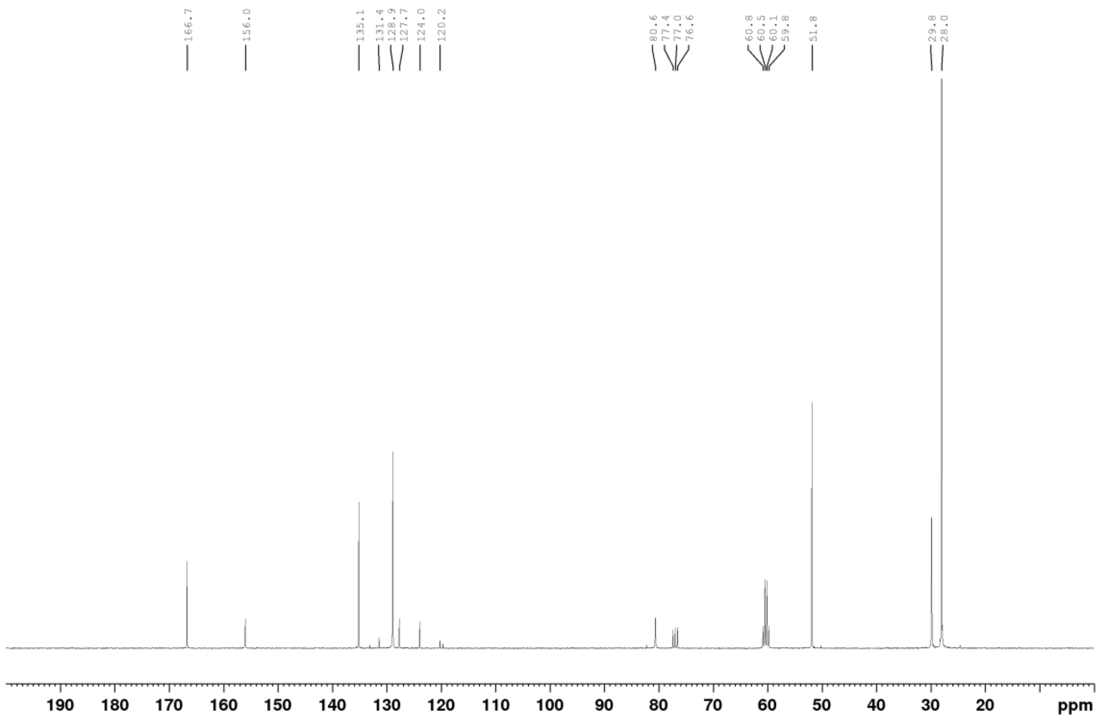

166.7
156.0
135.1
131.4
128.9
127.7
124.0
120.2
80.6
77.4
77.0
76.6
60.8
60.5
60.1
59.8
51.8
29.8
28.0
190
180
170
160
150
140
130
120
110
100
90
80
70
60
50
40
30
20
ppm

Benzyl 2-(1,1,1-trifluoro-4-(methoxycarbonyl)pent-4-en-2-yl)hydrazine-1-carboxylate (5b)

The product **5b** is obtained following the general procedure (**B**) as a yellow oil (0.633 g, 81%).

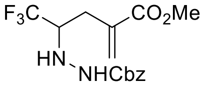

Chemical Formula: $C_{15}H_{17}F_3N_2O_4$
Exact Mass: 346,1140

^{1}H NMR (200 MHz, $CDCl_3$): δ = 7.33 (m, 5H, Haro), 6.59 (brs, 1H, NH), 6.38 (s, 1H, =CH_2), 5.80 (s, 1H, =CH_2), 5.11 (s, 2H, CH_2Cbz), 3.97 (m, 1H, NH), 3.79 (s, 3H, OCH_3), 3.66 (m, 1H, $CHCF_3$), 2.77 (dd, $^3J_{H,H}$ = 3.0 Hz, $^3J_{H,H}$ = 14.4 Hz, 1H, CH_2), 2.49 (dd, $^3J_{H,H}$ = 4.2 Hz, $^3J_{H,H}$ = 14.5 Hz, 1H, CH_2).

^{19}F NMR (188 MHz, $CDCl_3$): δ = - 75.49 (d, $^3J_{H,H}$ = 6.6 Hz, 3F)

^{13}C NMR (75 MHz, $CDCl_3$): δ = 167.0 (CO), 156.8 (COCbz), 135.9 (=Cq), 135.2 (Caro), 129.3, 128.5, 128.1 (CHaro), 125.9 (q, $^1J_{H,H}$ = 279 Hz, CF_3), 120.3 (=CH_2), 67.3 (CH_2Cbz), 60.7 (q, $^2J_{H,H}$ = 26.9 Hz, $CHCF_3$), 52.2 (CH_3), 29.8 (CH_2).

HRMS (ESI+TOF) m/z $C_{15}H_{17}F_3N_2O_4$ $[M+Na]^+$ calc. 369.1033, found. 369.1123

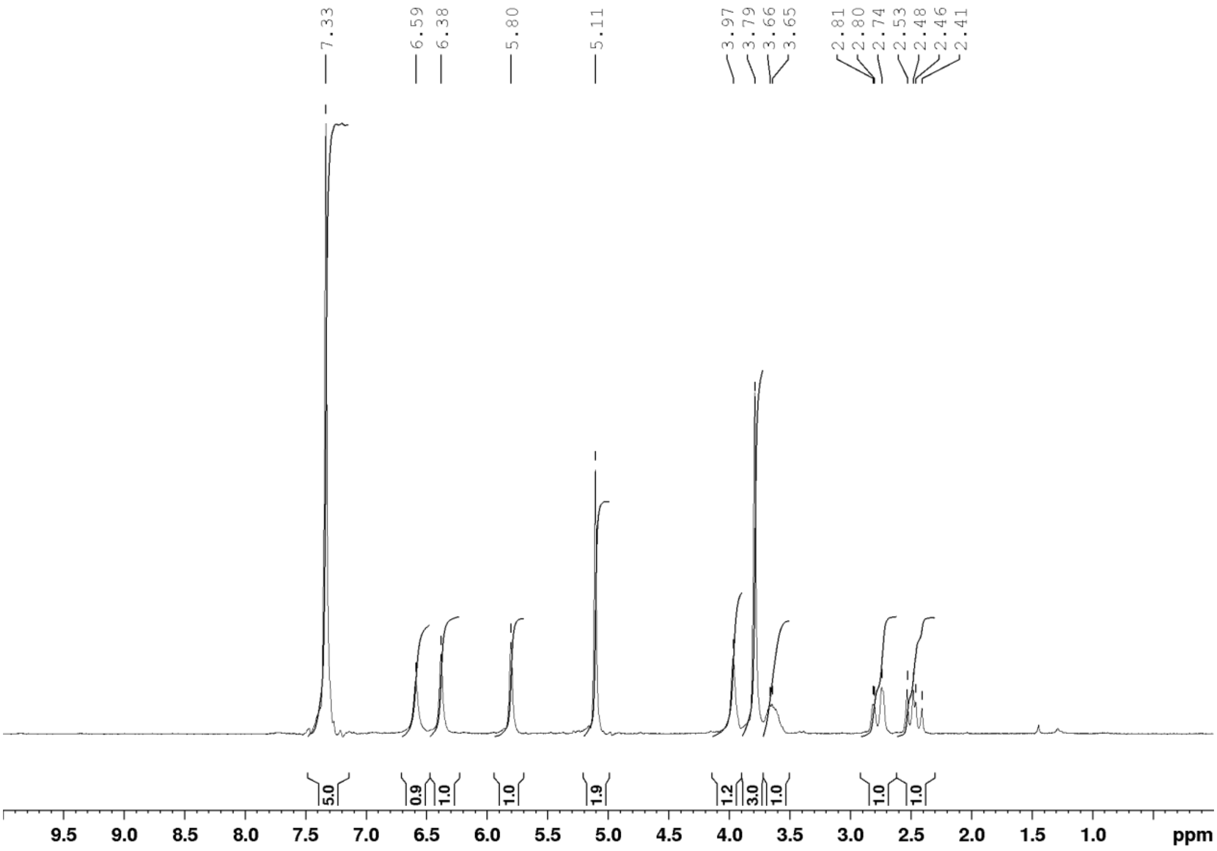

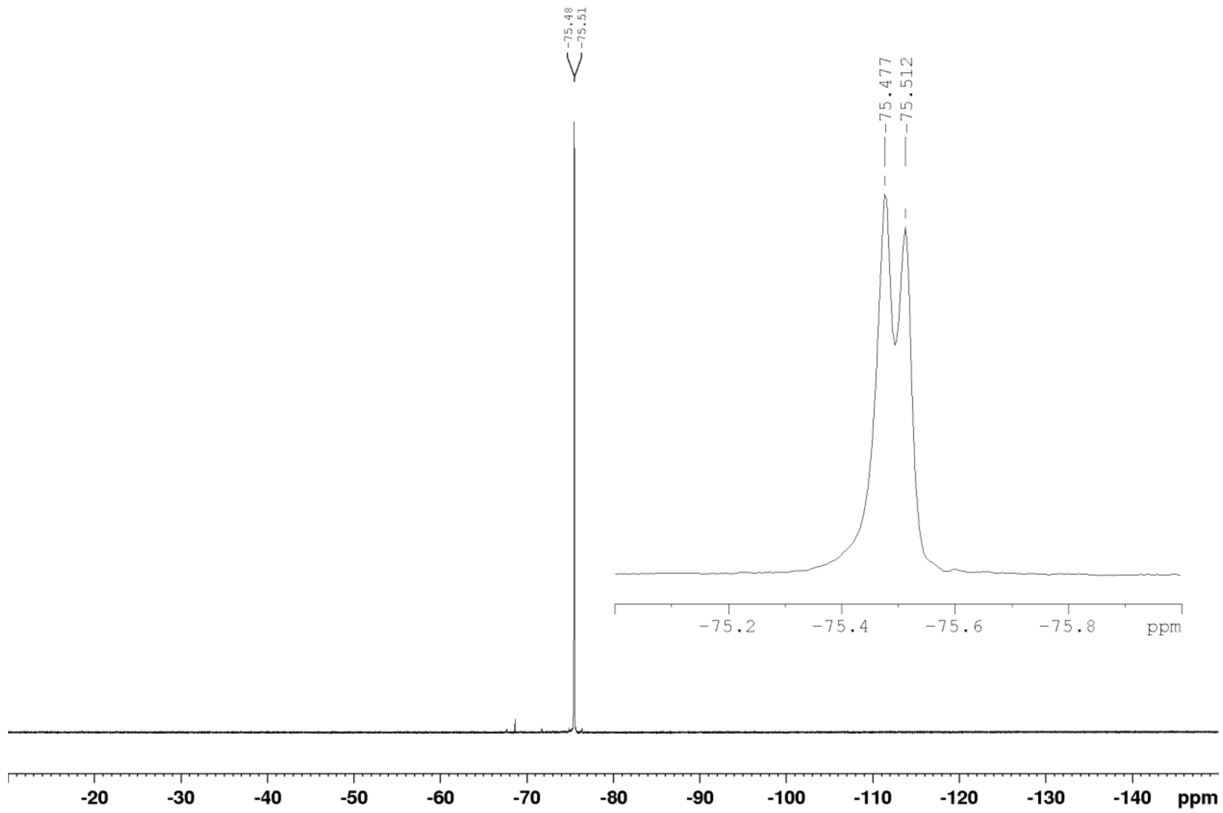
-75.48
-75.51
-75.477
-75.512
-75.2
-75.4
-75.6
-75.8
ppm
-20
-30
-40
-50
-60
-70
-80
-90
-100
-110
-120
-130
-140
ppm

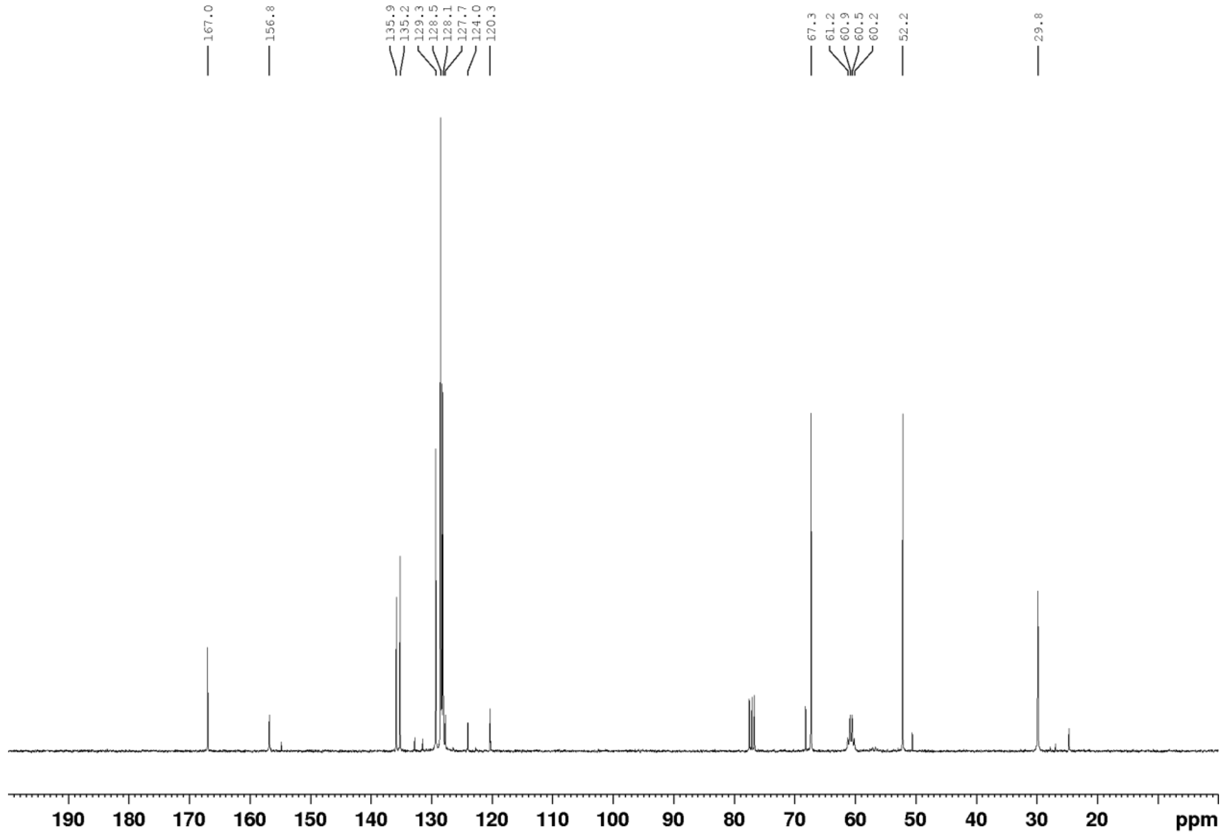
167.0
156.8
135.9
135.2
129.3
128.5
128.1
127.7
124.0
120.3
67.3
61.2
60.9
60.5
60.2
52.2
29.8
190
180
170
160
150
140
130
120
110
100
90
80
70
60
50
40
30
20
ppm

***tert*-Butyl -2-(1,1-difluoro-4-(methoxycarbonyl)pent-4-en-2-yl)hydrazine-1-carboxylate (5c)**

The product **5c** is obtained following the general procedure (**B**) as a yellow oil (0.669g, 88%).

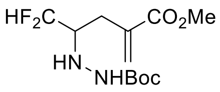

Chemical Formula: $C_{12}H_{20}F_2N_2O_4$
Exact Mass: 294,1391

^{1}H NMR (200 MHz, $CDCl_3$): δ = 6.30 (s, 1H, =CH_2), 6.29 (s, 1H, NHBoc), 5.77 (s, 1H, =CH_2), 5.76 (td, $^{3}J_{H,H}$ = 3.9 Hz, $^{2}J_{H,F}$ = 55.7 Hz, 1H, CF_2H), 3.94 (brs, 1H, NH), 3.76 (s, 3H, OCH_3), 3.35 (m, 1H, $CHCF_2H$), 2.69 (dd, $^{3}J_{H,H}$ = 8.7 Hz, $^{2}J_{H,H}$ = 14.5 Hz, 1H, CH_2), 2.42 (dd, $^{3}J_{H,H}$ = 4.1 Hz, $^{2}J_{H,H}$ = 14.5 Hz, 1H, CH_2), 1,42 (m, 9H, $(CH_3)_3$).

^{19}F NMR (188 MHz, $CDCl_3$): δ = - 126.4 (dd, $^{2}J_{F,F}$ = 288 Hz, $^{2}J_{H,F}$ = 54 Hz, 1F), -128.7 (dd, $^{2}J_{F,F}$ = 288 Hz, $^{2}J_{H,F}$ = 54 Hz, 1F).

^{13}C NMR (75 MHz, $CDCl_3$): δ = 167.1 (CO), 156.4 (COBoc), 135.9 (=Cq), 128.2 (=CH_2), 116.2 (t, $^{1}J_{C,F}$ = 243 Hz, CHF_2), 80.6 (Cq), 60.4 (t, $^{2}J_{C,F}$ = 21 Hz, CH-CHF_2), 51.9 (OCH_3), 28.9 (CH_2), 28.1 ($(CH_3)_3$).

HRMS (ESI+TOF) m/z $C_{12}H_{20}F_2N_2O_4$ $[M+Na]^+$ calc. 317.1283, found. 317.1266

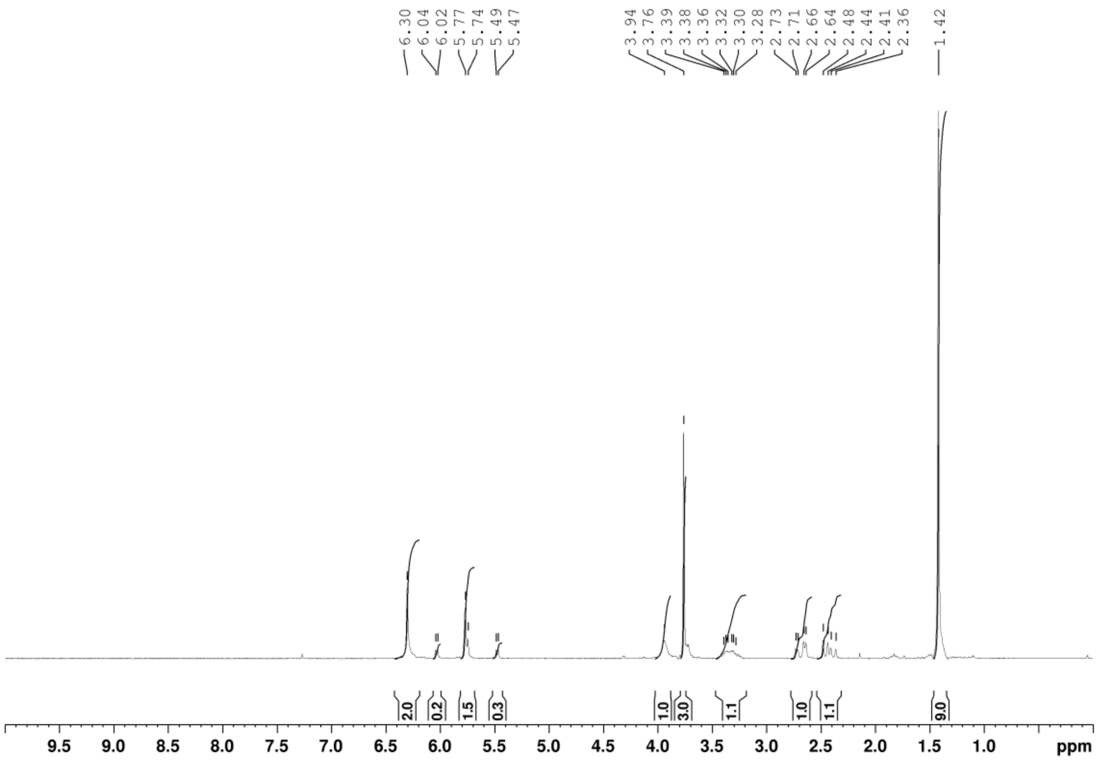

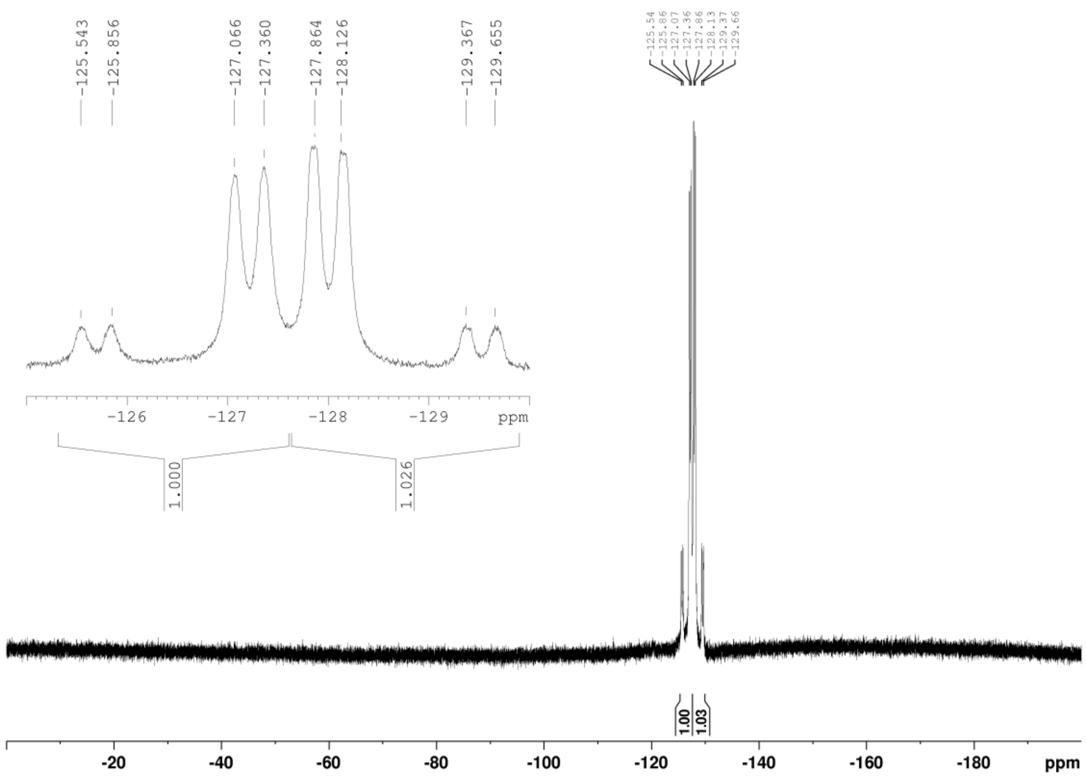
-125.543
-125.856
-127.066
-127.360
-127.864
-128.126
-129.367
-129.655
-126
-127
-128
-129
ppm
1.000
1.026
-125.54
-125.86
-127.07
-127.36
-127.86
-128.13
-129.37
-129.66
1.00
1.03
-20
-40
-60
-80
-100
-120
-140
-160
-180
ppm

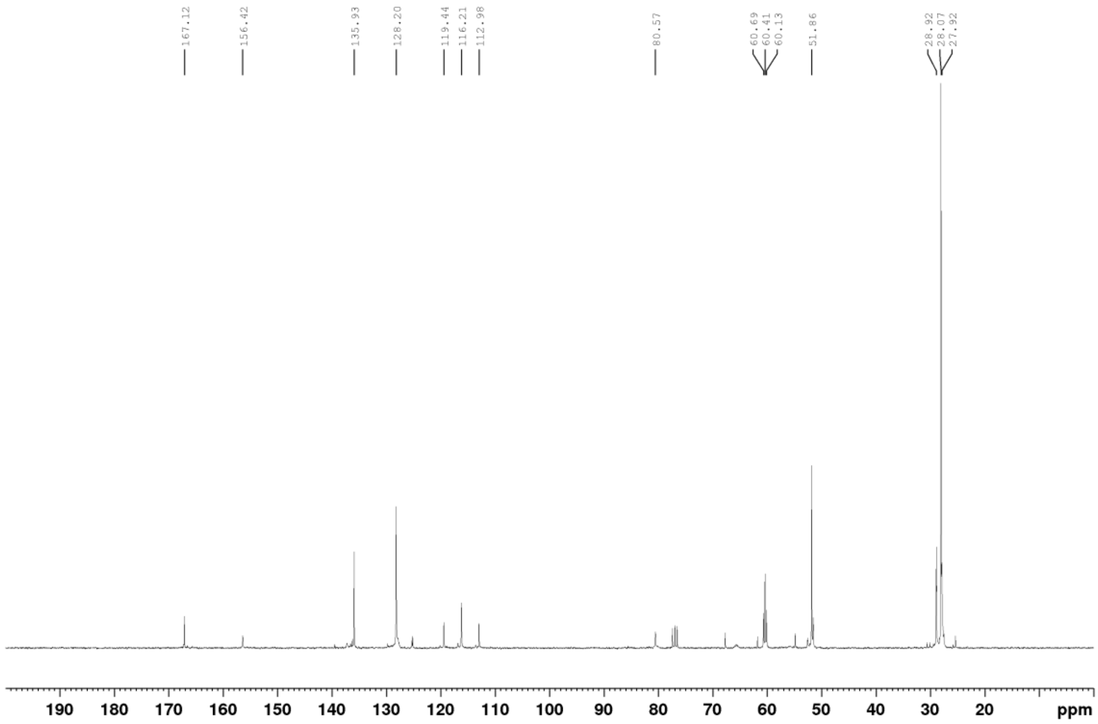
167.12
156.42
135.93
128.20
119.44
116.21
112.98
80.57
60.69
60.41
60.13
51.86
28.92
28.07
27.92
190 180 170 160 150 140 130 120 110 100 90 80 70 60 50 40 30 20 ppm

Benzyl -2-(1,1-difluoro-4-(methoxycarbonyl)pent-4-en-2-yl)hydrazine-1-carboxylate (5d)

The product **5d** is obtained following the general procedure (**B**) as a yellow oil (0.566 g, 79%).

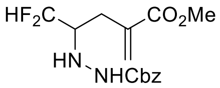

Chemical Formula: $C_{15}H_{18}F_2N_2O_4$
Exact Mass: 328,1235

^{1}H NMR (200 MHz, $CDCl_3$): δ = 7.35 (m, 5H, Haro), 6.47 (s, 1H, NHCbz), 6.34 (s, 1H, =CH_2), 5.79 (s, 1H, =CH_2), 5.75 (dt, $^3J_{H,H}$ = 3.9 Hz, $^2J_{H,F}$ = 55.7 Hz, 1H, CF_2H), 5.13 (s, 2H, CH_2Ph), 3.78 (s, 3H, OCH_3), 3.39 (m, 2H, NH and CH), 2.71 (dd, $^3J_{H,H}$ = 3.9 Hz, $^2J_{H,H}$ = 14.7 Hz, 1H, CH_2), 2.43 (dd, $^3J_{H,H}$ = 9.0 Hz, $^2J_{H,H}$ = 14.5 Hz, 1H, CH_2). ,

^{19}F NMR (188 MHz, $CDCl_3$): δ = - 126.4 (ddd, $^2J_{F,F}$ = 290 Hz, $^2J_{H,F}$ = 58 Hz, $^3J_{H,F}$ = 7 Hz, 1F), -128.3 (ddd, $^2J_{F,F}$ = 288 Hz, $^2J_{H,F}$ = 56 Hz, $^3J_{H,F}$ = 12 Hz, 1F).

^{13}C NMR (75 MHz, $CDCl_3$): δ = 167.2 (CO), 157.0 (CO_{Cbz}), 135.8 (=C), 128.5 (Caro), 128.4, 128.1, 128.0 (CHaro), 125.4 (=CH_2), 116.2 (t, $^1J_{C,F}$ = 242 Hz, CF_2H), 67.1 (CH_2Cbz), 60.5 (t, $^2J_{C,F}$ = 20 Hz, $CHCF_2H$), 52.0 (OCH_3), 29.0 (CH_2).

HRMS (ESI+TOF) m/z $C_{15}H_{18}F_2N_2O_4$ $[M+Na]^+$ calc. 351.1127, found. 351.1114

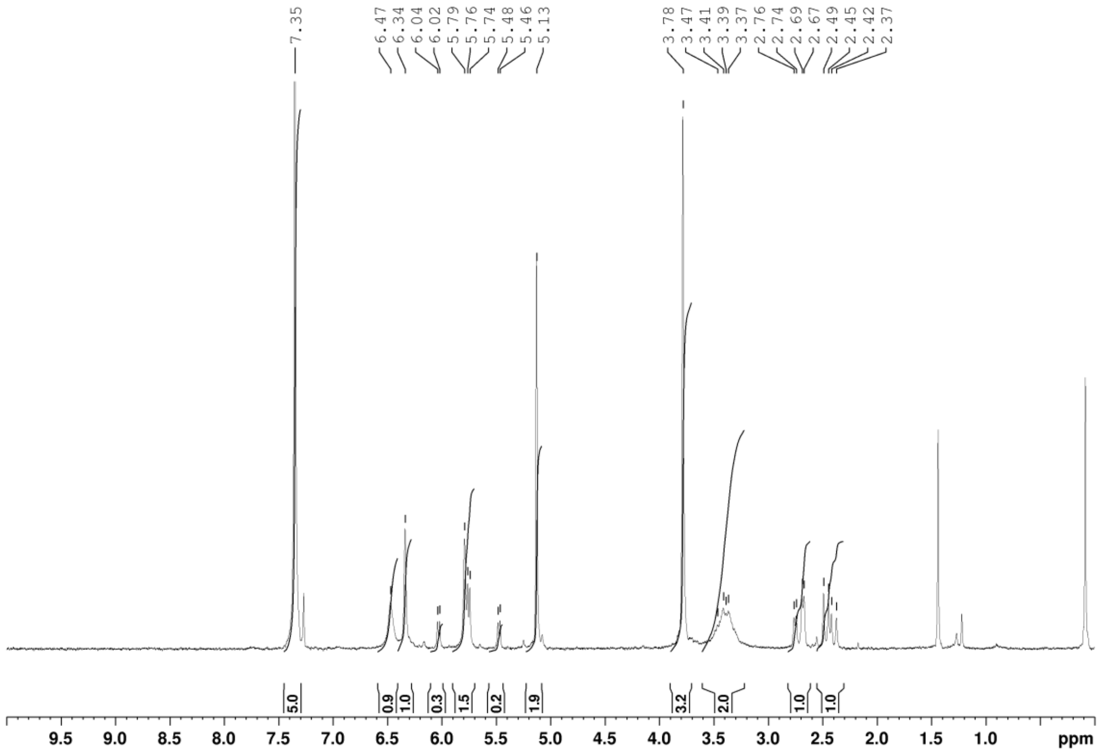

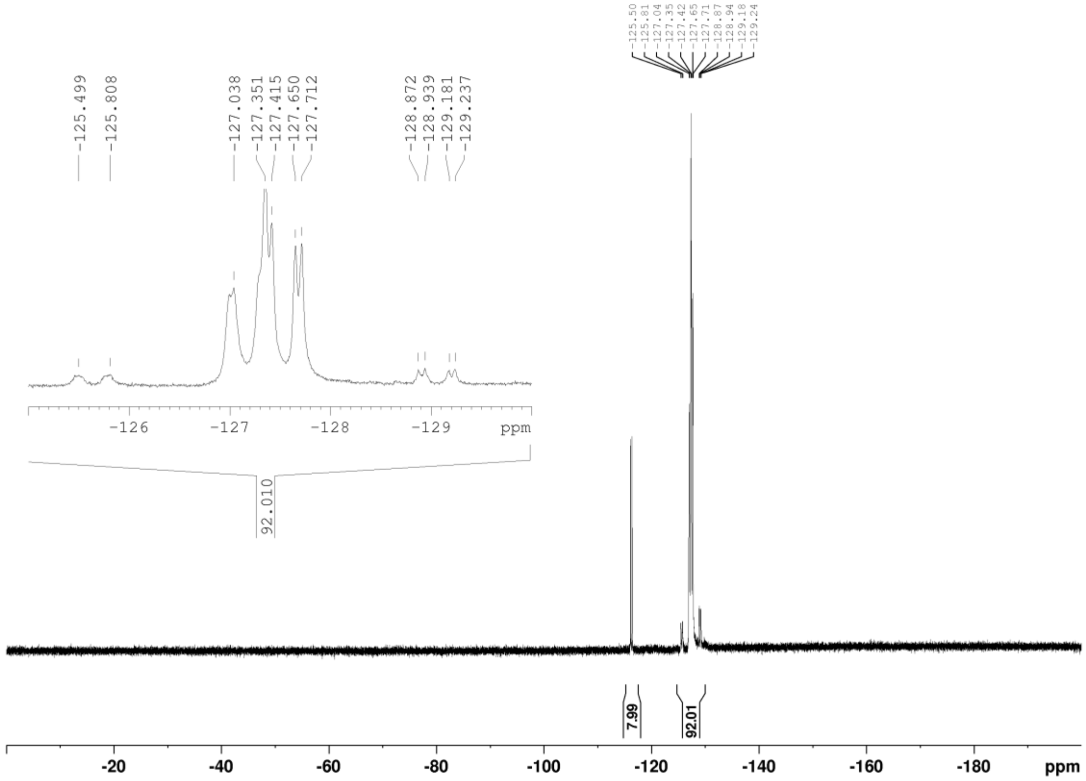
-125.50
-125.81
-127.04
-127.35
-127.42
-127.65
-127.71
-128.87
-128.94
-129.18
-129.24
-125.499
-125.808
-127.038
-127.351
-127.415
-127.650
-127.712
-128.872
-128.939
-129.181
-129.237
-126
-127
-128
-129
ppm
92.010
7.99
92.01
-20
-40
-60
-80
-100
-120
-140
-160
-180
ppm

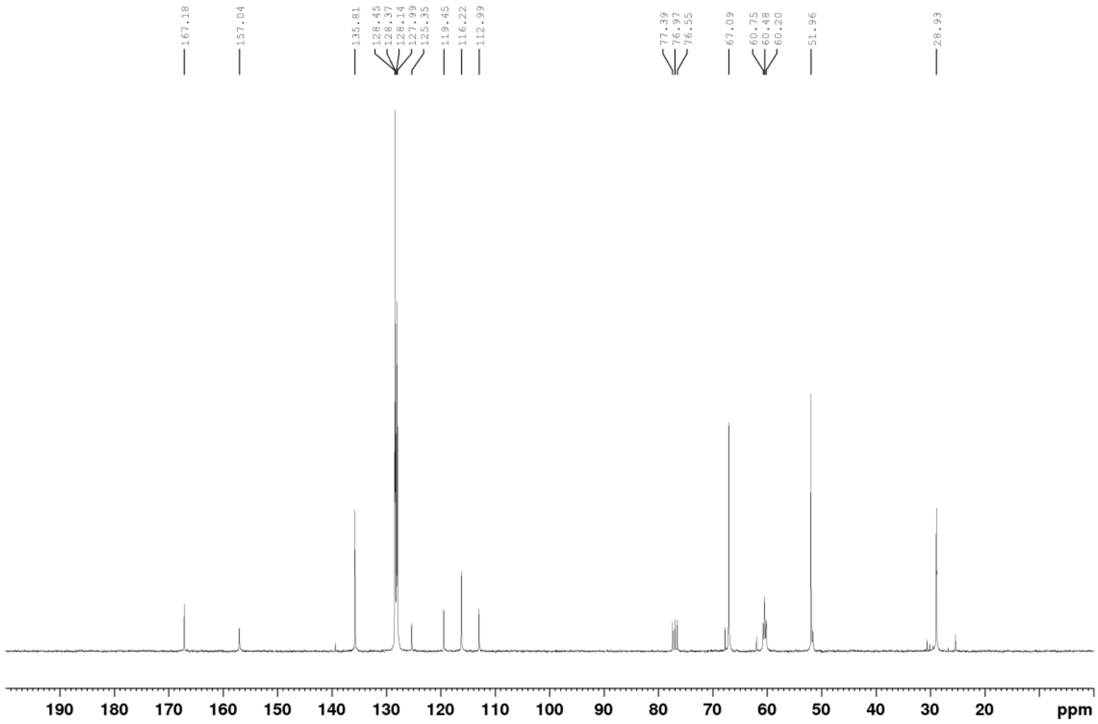
167.18
157.04
135.81
128.45
128.37
128.14
127.99
125.35
119.45
116.22
112.99
77.39
76.97
76.55
67.09
60.75
60.48
60.20
51.96
28.93
190
180
170
160
150
140
130
120
110
100
90
80
70
60
50
40
30
20
ppm

Methyl 4-(2-(((benzyloxy)carbonyl)-L-phenylalanyl)hydrazineyl)-5,5,5-trifluoro-2-methylenepentanoate (5e)

The product **5e** is obtained following the general procedure (**B**) as a yellow oil (0.580 g, 66%) as a mixture of two diastereoisomers (ratio: 52/48).

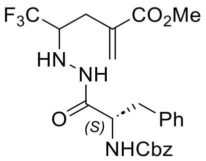

Chemical Formula: $C_{24}H_{26}F_3N_3O_5$
Exact Mass: 493,1825

^{1}H NMR (200 MHz, $CDCl_3$): δ = 7.89 (brs, 1H, NHCO), 7.25-7.02 (m, 10H, Haro), 6.29 and 6.28 (brs, 1H, $=CH_2$), 5.68 and 5.66 (brs, 1H, $=CH_2$), 5.46 (d, $^{3}J_{H,H}$ = 8.1 Hz, 0.5H, NHCbz), 5.33 (d, $^{3}J_{H,H}$ = 8.36 Hz, 0.5 H, NHCbz), 4.94 (s, 2H, CH_2Cbz), 4.68 (d, $^{3}J_{H,H}$ = 6.16 Hz, 0.5 H, NHN), 4.60 (d, $^{3}J_{H,H}$ = 6.16 Hz, 0.5 H, NHN), 4.32 (q, $^{3}J_{H,H}$ = 5.2 Hz, 1 H, CHα), 3.71 and 3.69 (2s, 3H, OCH_3), 3.42-3.38 (m, 0.5H, CH), 3.18-3.14 (m, 0.5H, CH), 2.92 (m, 2H, CH_2Ph), 2.60 (td, $^{2}J_{H,H}$ = 14.4 Hz, $^{3}J_{H,H}$ = 3.7 Hz, 1H, CH_2), 2.32 (dd, $^{2}J_{H,H}$ = 14.8 Hz, $^{3}J_{H,H}$ = 10.6 Hz, 1H, CH_2).

^{19}F NMR (188 MHz, $CDCl_3$): δ = -75.2 (d, $^{3}J_{H,F}$ = 6.96 Hz, 3F), -75.7 (d, $^{3}J_{H,F}$ = 6.63 Hz, 3F).

^{13}C NMR (75 MHz, $CDCl_3$): δ = 170.7 (CONH), 167.1 and 166.9 (COCbz), 155.9 (CO), 136.0 and 135.8 (=C), 135.1 and 134.9 (Caro), 129.4, 129.2, 128.7, 128.5, 128.3, 128.2, 128.2, 128.0, 127.9, 127.1 (CHaro), 126.2 and 125.7 ($=CH_2$), 125.8 (q, $^{1}J_{C,F}$ = 281Hz, CF_3), 125.7 (q, $^{1}J_{C,F}$ = 280 Hz, CF_3), 67.2 and 67.1 (CH_2Cbz), 60.7 (q, $^{2}J_{H,F}$ = 27 Hz, $CHCF_3$), 54.7 and 54.5 (CHα), 52.2 (OCH_3), 38.7 and 38.3 (CH_2Ph), 29.8 and 29.8 (CH_2).

HRMS (ESI+TOF) m/z $C_{24}H_{26}F_2N_3O_5$ $[M+H]^+$ calc. 494.1898, found. 494.1905

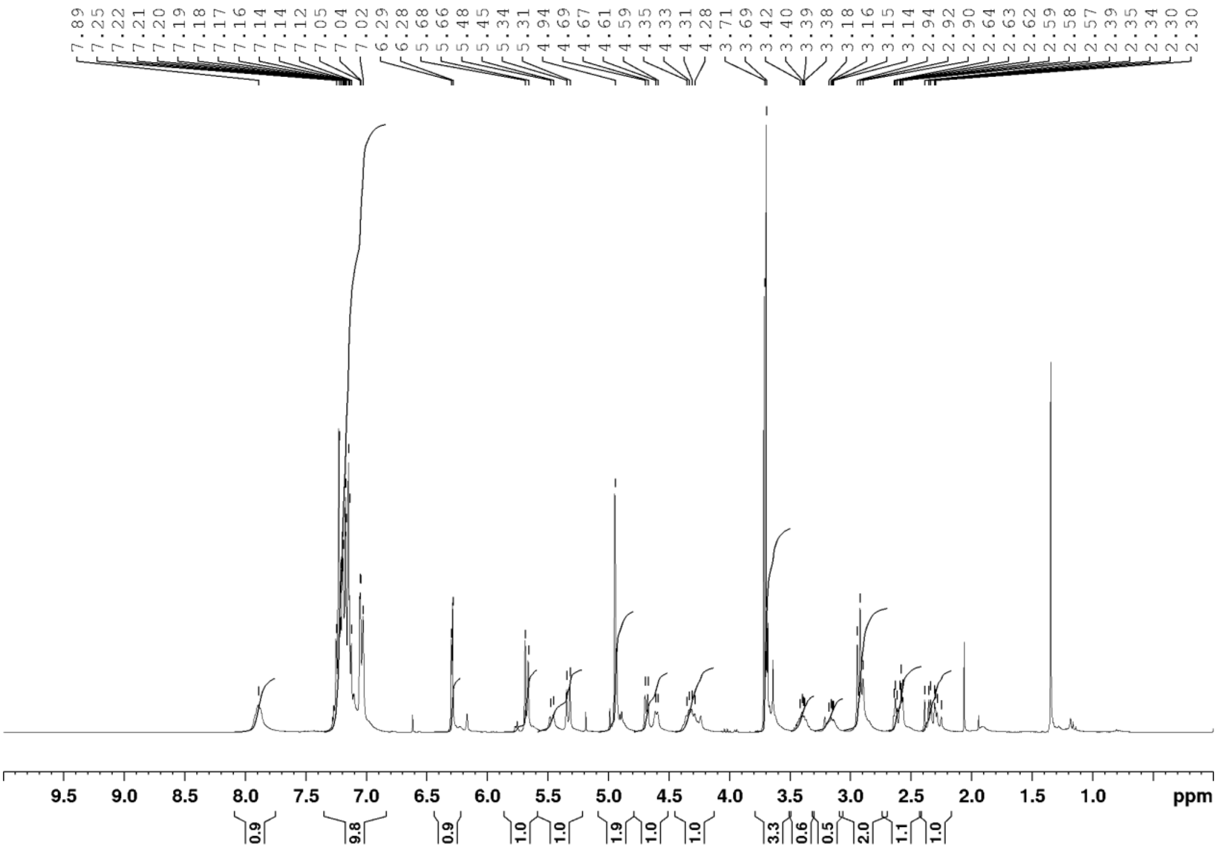

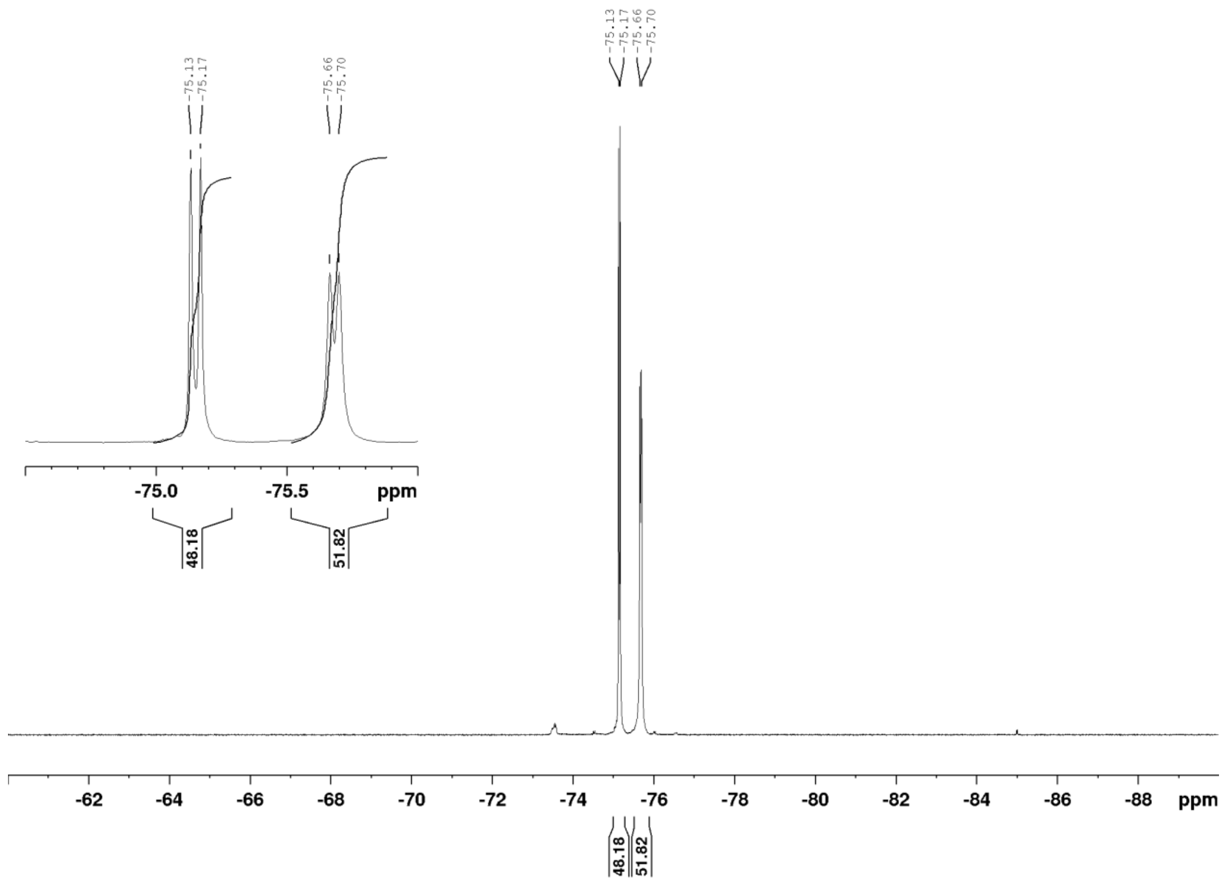
-75.13
-75.17
-75.66
-75.70
-75.0
-75.5
ppm
48.18
51.82
-62
-64
-66
-68
-70
-72
-74
-76
-78
-80
-82
-84
-86
-88
ppm

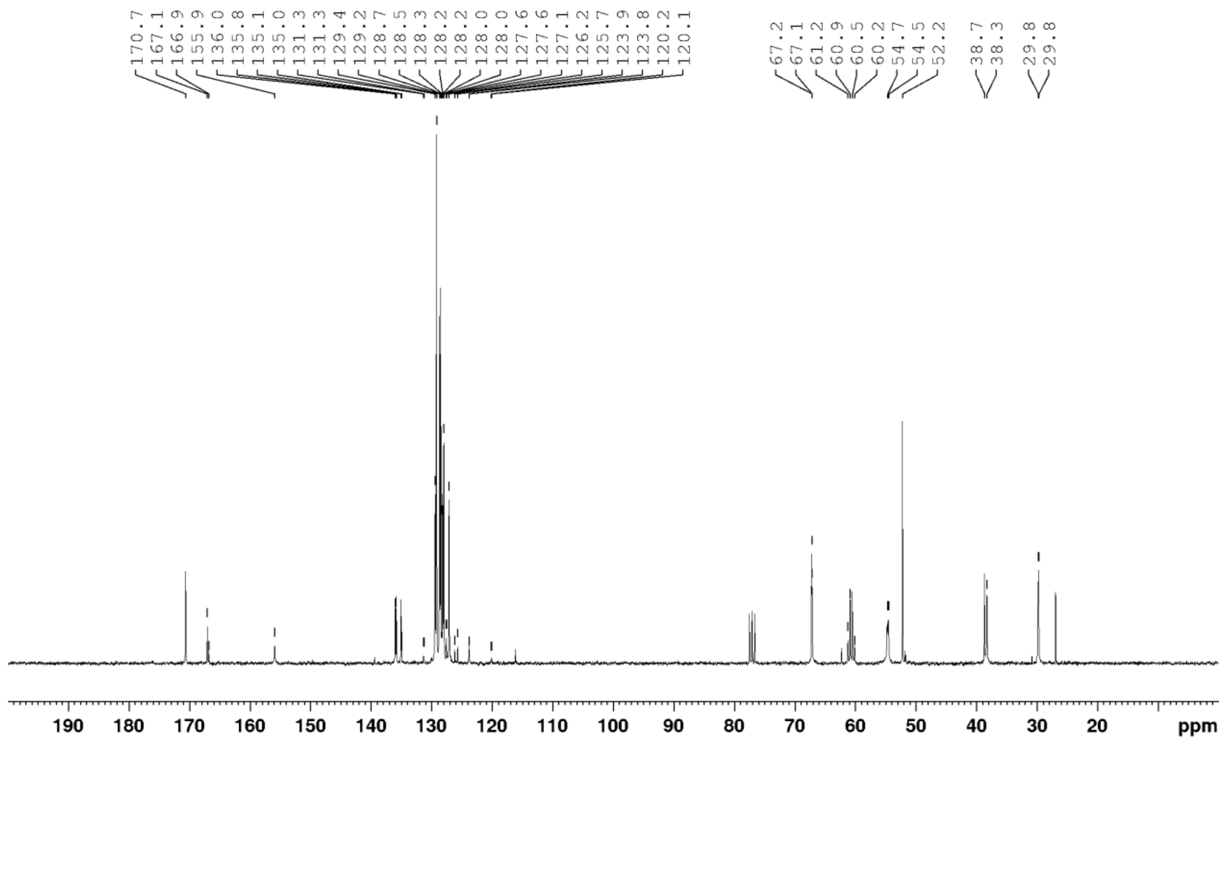
170.7
167.1
166.9
155.9
136.0
135.8
135.1
135.0
131.3
131.3
129.4
129.2
128.7
128.5
128.3
128.2
128.2
128.0
128.0
127.6
127.1
126.2
125.7
123.9
123.8
120.2
120.1
67.2
67.1
61.2
60.9
60.5
60.2
54.7
54.5
52.2
38.7
38.3
29.8
29.8
190
180
170
160
150
140
130
120
110
100
90
80
70
60
50
40
30
20
ppm

Methyl 4-(2-(((benzyloxy)carbonyl)-L-phenylalanyl)hydrazineyl)-5,5-difluoro-2-methylenepentanoate (5f)

The product **5f** is obtained following the general procedure (**B**) as a yellow oil (1.07 g, 75%) as a mixture of two diastereoisomers (ratio: 51/49).

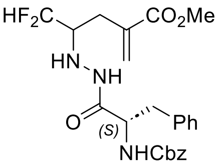

Chemical Formula: $C_{24}H_{27}F_2N_3O_5$
Exact Mass: 475,1919

^{1}H NMR (200 MHz, $CDCl_3$): δ = 7.76 (brs, 1H, NHCO), 7.28-7.07 (m, 10H, Haro), 6.23 (d, $^3J_{H,H}$ = 4.5 Hz, 1H, =CH_2), 5.65 (brs, 1H, =CH_2), 5.48 (td, $^2J_{H,F}$ = 55.7Hz, $^3J_{H,H}$ = 4.3 Hz, 0.5H, CF_2H), 5.39 (d, $^3J_{H,H}$ = 7.6 Hz, 1H, NHCbz), 5.34 (d, $^3J_{H,H}$ = 7.7 Hz, 1H, NHCbz), 5.21 (td, $^2J_{H,F}$ = 56.0 Hz, $^3J_{H,H}$ = 4.1 Hz, 0.5H, CF_2H), 4.97 (s, 2H, CH_2Cbz), 4.66 (sl, 1H, NHN), 4.30 (q, $^3J_{H,H}$ = 7.7 Hz, 1H, CHα), 3.68 and 3.67 (2s, 3H, OCH_3), 3.09 (m, 1H, CH), 2.97 and 2.95 (2d, $^3J_{H,H}$ = 6.6 Hz and $^3J_{H,H}$ = 7.2 Hz, 2H, CH_2Ph), 2.48 (dd, $^2J_{H,H}$ = 14.9 Hz, $^3J_{H,H}$ = 3.9 Hz, 1H, CH_2), 2.22 (dd, $^2J_{H,H}$ = 14.7 Hz, $^3J_{H,H}$ = 9.5 Hz, 1H, CH_2)

^{19}F NMR (188 MHz, $CDCl_3$): δ = -125.9 (ddd, $^2J_{F,F}$ = 287 Hz, $^2J_{H,F}$ = 55 Hz, $^3J_{H,F}$ = 10.1 Hz, 1F), -126.1 (ddd, $^2J_{F,F}$ = 286 Hz, $^2J_{H,F}$ = 55 Hz, $^3J_{H,F}$ = 8.4 Hz, 1F), -128.5 (ddd, $^2J_{F,F}$ = 287 Hz, $^2J_{H,F}$ = 55 Hz, $^3J_{H,F}$ = 11.5 Hz, 1F), -128.9 (ddd, $^2J_{F,F}$ = 286 Hz, $^2J_{H,F}$ = 55 Hz, $^3J_{H,F}$ = 12.4 Hz, 1F).

^{13}C NMR (75 MHz, $CDCl_3$): δ = 170.5 and 170.4 (CONH), 167.5 and 167.3 (COCbz), 155.9 (CO), 136.0 and 135.8 (=C), 129.2, 128.8, 128.7, 128.2, 128.0 (CHaro), 127.2 (=CH_2), 116.1 (t, $^1J_{C,F}$ = 244 Hz, CF_2H), 116.0 (t, $^1J_{C,F}$ = 244 Hz, CF_2H), 67.2 and 67.1 (CH_2Cbz), 60.8 (t, $^2J_{C,F}$ = 21 Hz, $CHCF_2H$), 54.9 (CHα), 52.2 (OCH_3), 38.6 and 38.3 (CH_2Ph), 28.9 and 28.8 (CH_2).

HRMS (ESI+TOF) m/z $C_{24}H_{28}F_2N_3O_5$ $[M+H]^+$ calc. 476.1997, found. 476.2000

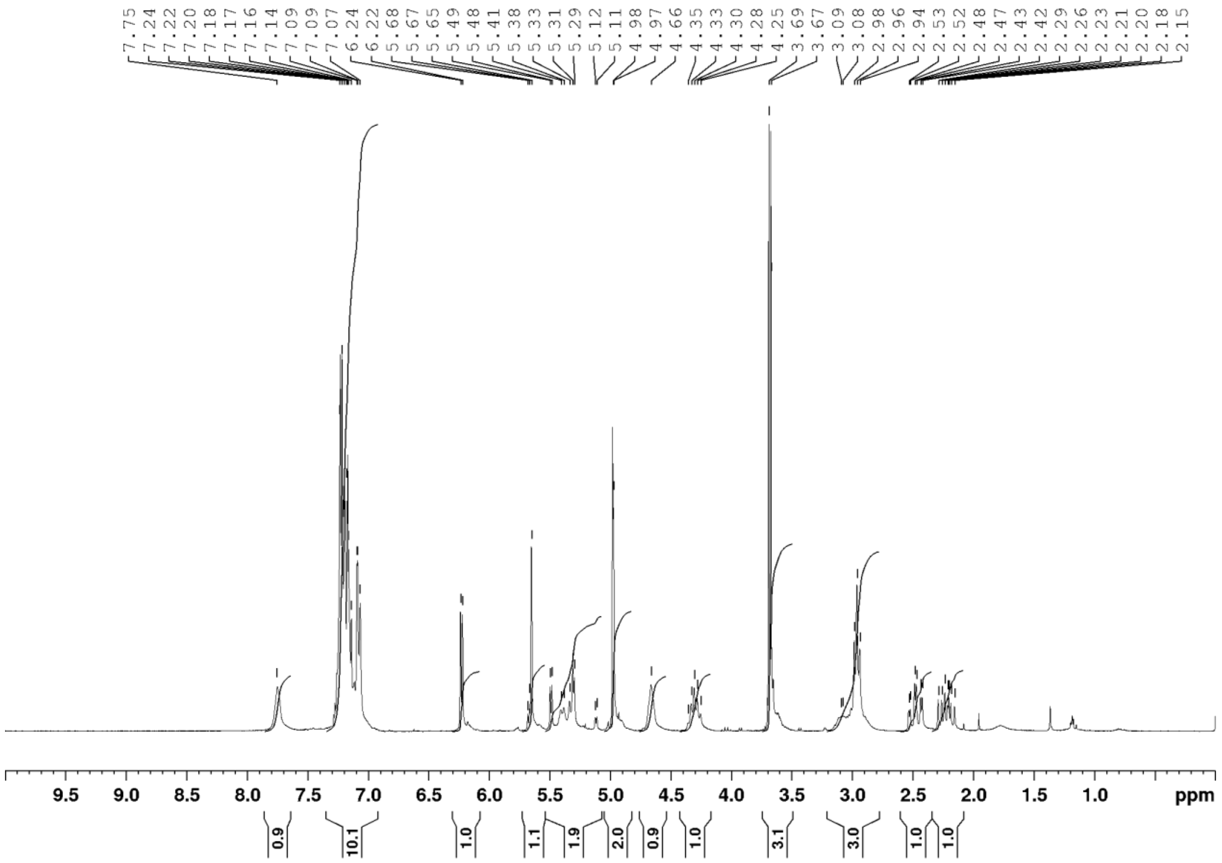

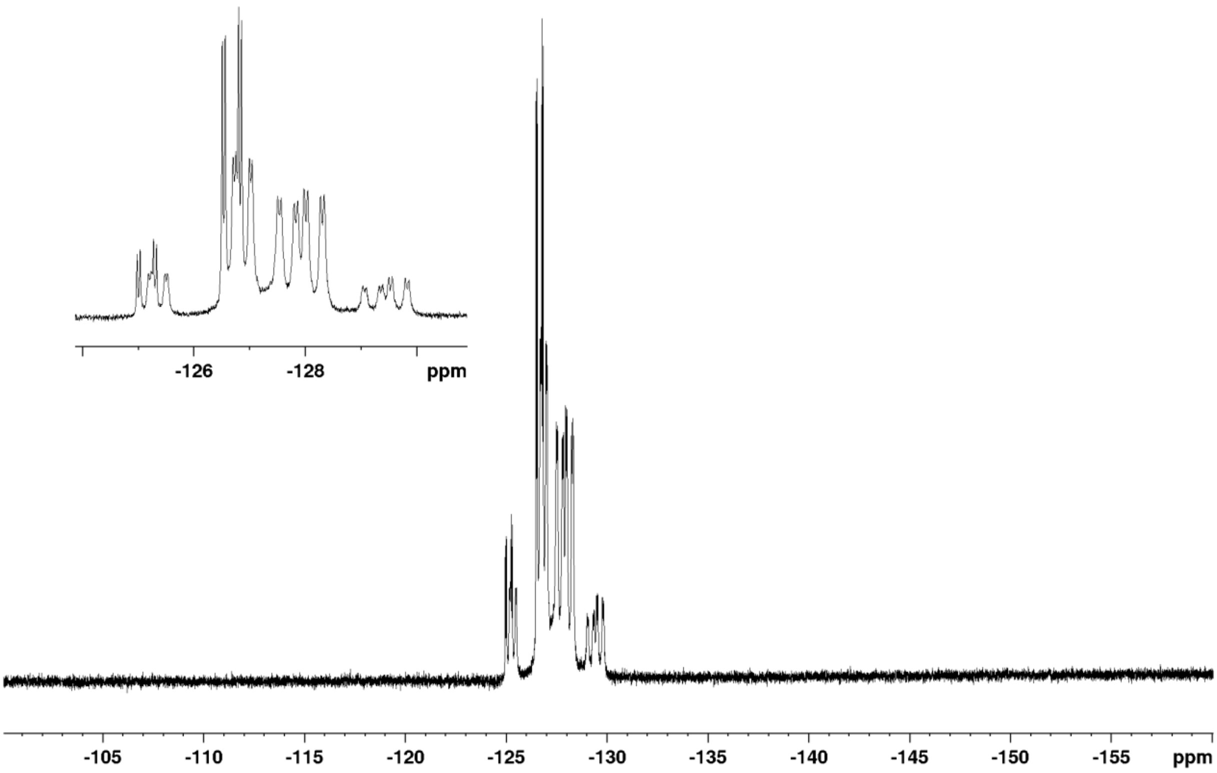
-126
-128
ppm
-105
-110
-115
-120
-125
-130
-135
-140
-145
-150
-155
ppm

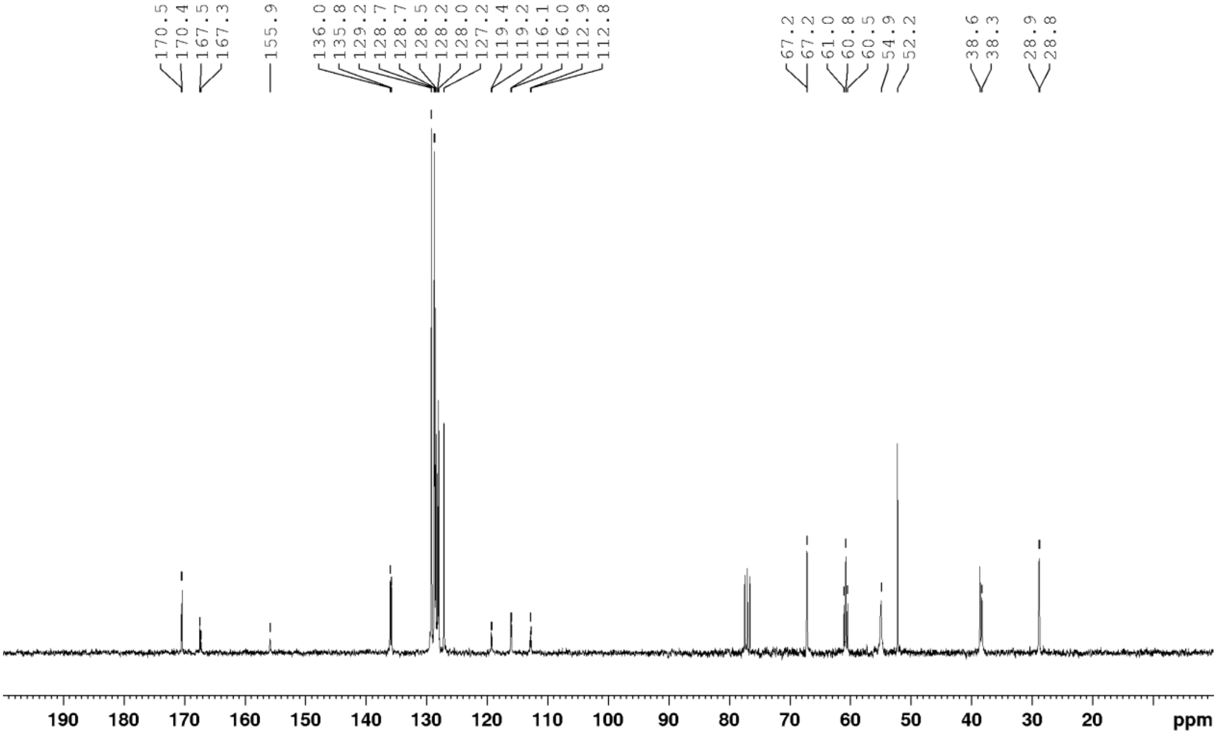
170.5
170.4
167.5
167.3
155.9
136.0
135.8
129.2
128.7
128.7
128.5
128.2
128.0
127.2
119.4
119.2
116.1
116.0
112.9
112.8
67.2
67.2
61.0
60.8
60.5
54.9
52.2
38.6
38.3
28.9
28.8
190
180
170
160
150
140
130
120
110
100
90
80
70
60
50
40
30
20
ppm

c) Hydrazones 6a–f

***tert*-Butyl (E)-2-(1,1,1-trifluoro-4-(methoxycarbonyl)pent-4-en-2-ylidene)hydrazine-1-carboxylate (6a)**

The product **6a** is obtained following the general procedure (**C**) as a slightly yellow oil (0.347 g, 80% (I_2) / 77%(NBS))

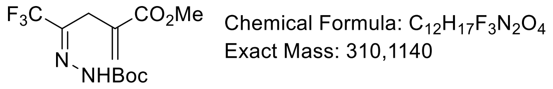

^{1}H NMR (200 MHz, $CDCl_3$): δ= 9.39 (s, 1H, NHBoc), 6.41 (s, 1H, $=CH_2$), 5.86 (s, 1H, $=CH_2$), 3.84 (s, 3H, OCH_3), 3.37 (s, 2H, CH_2), 1.53 (s, 9H, $(CH_3)_3$).

^{19}F NMR (188 MHz, $CDCl_3$): δ = - 68.69 (s, 3F)

^{13}C NMR (75 MHz, $CDCl_3$): δ = 167.6 (COBoc), 152.0 (CO), 136.3 (q, $^{2}J_{C,F}$ = 34 Hz, C=N), 132.1 (=C), 130.1 ($=CH_2$), 120.9 (q, $^{1}J_{C,F}$ = 274 Hz, CF_3), 82.2 (Cq), 52.8 (OCH_3), 28.0 (CH_2), 27.6 (CH_3).

HRMS (ESI+TOF) m/z $C_{12}H_{17}F_3N_2O_4$ $[M+Na]^+$ calc. 333.1038, found. 333.1152

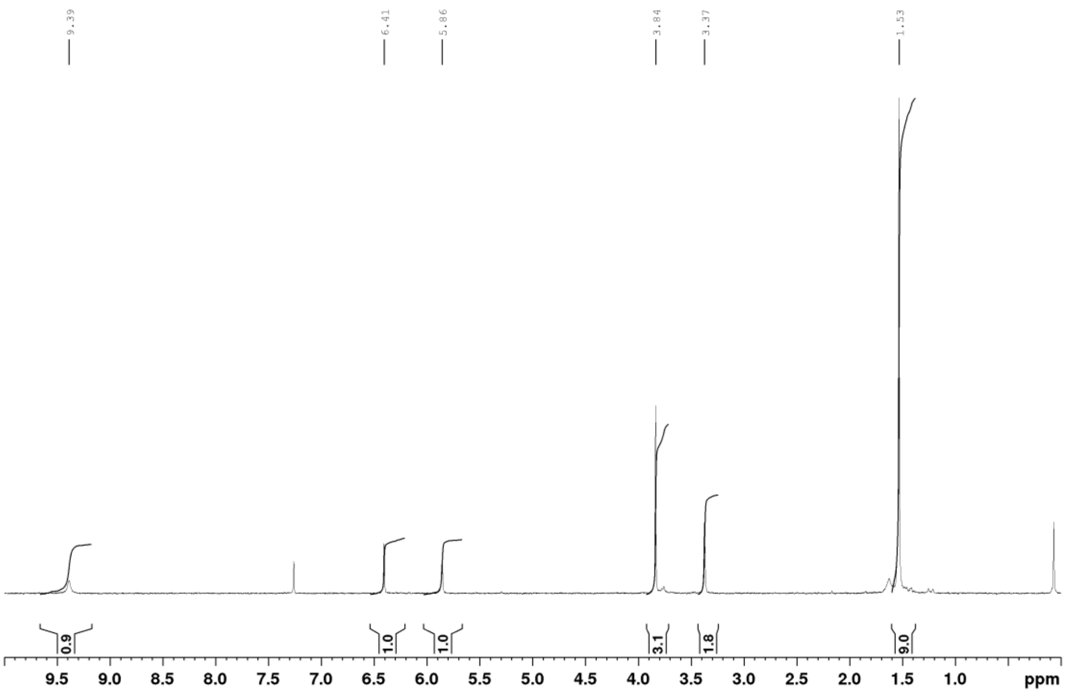

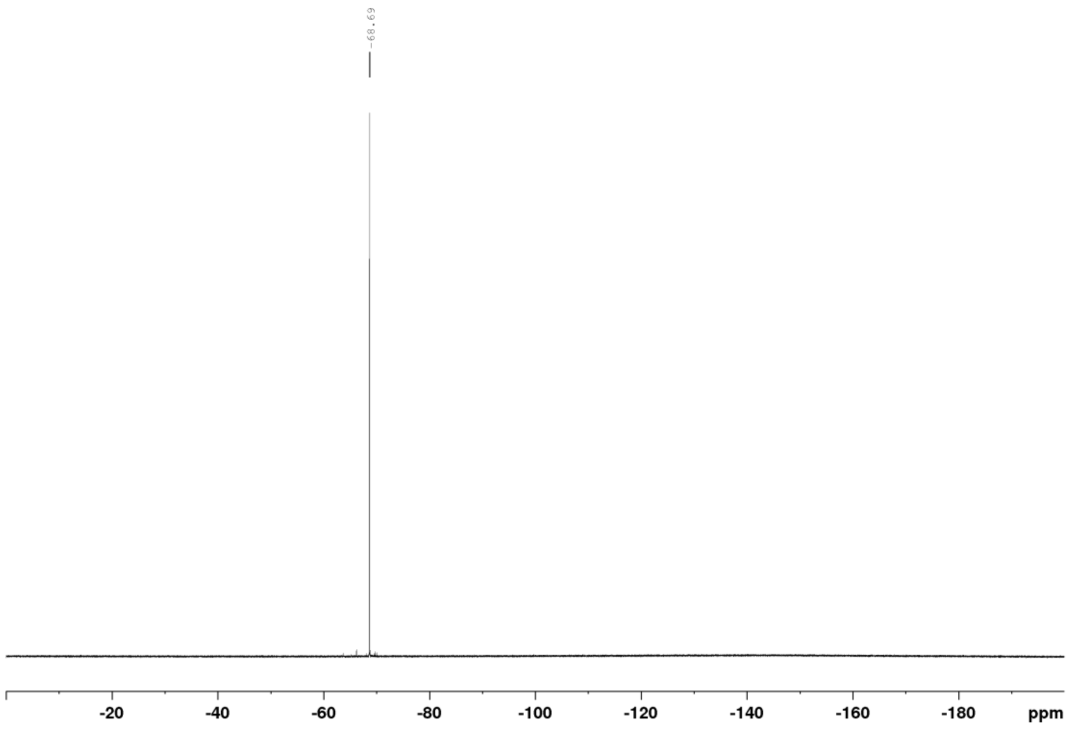

-68.69
-20
-40
-60
-80
-100
-120
-140
-160
-180
ppm

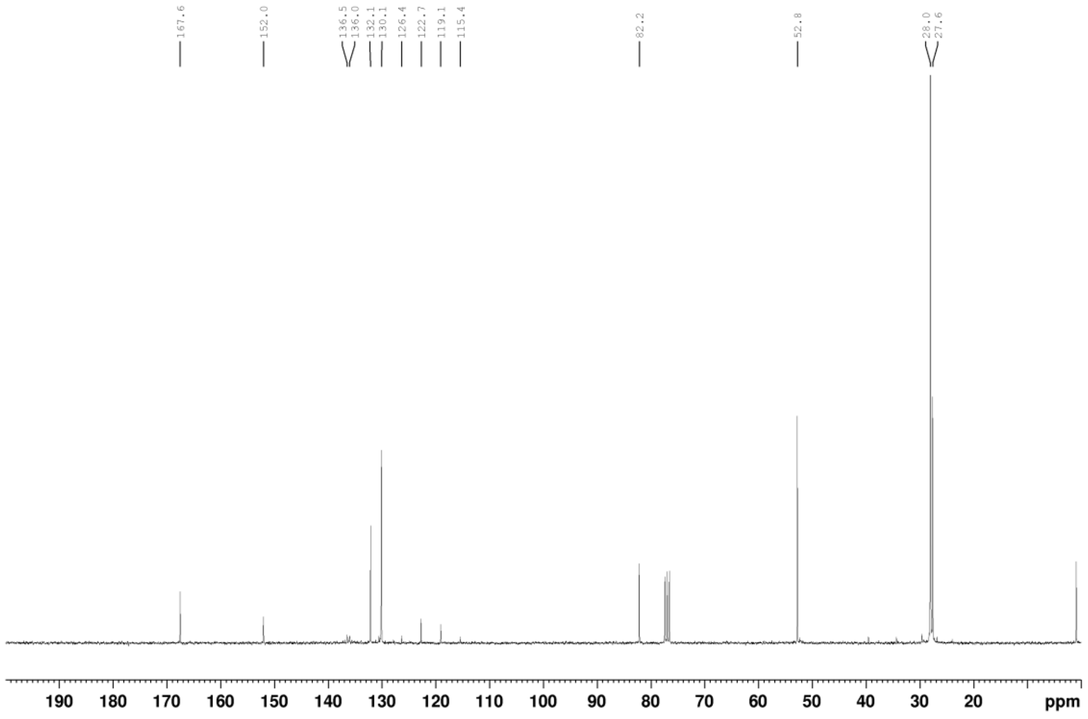

167.6
152.0
136.5
136.0
132.1
130.1
126.4
122.7
119.1
115.4
82.2
52.8
28.0
27.6
190
180
170
160
150
140
130
120
110
100
90
80
70
60
50
40
30
20
ppm

Benzyl (E)-2-(1,1,1-trifluoro-4-(methoxycarbonyl)pent-4-en-2-ylidene)hydrazine-1-carboxylate (6b)

The product **6b** is obtained following the general procedure (**C**) as a slightly yellow oil (0.470 g, 80% (I_2)/82% (NBS))

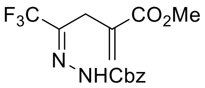

Chemical Formula: $C_{15}H_{15}F_3N_2O_4$
Exact Mass: 344,0984

^{1}H NMR (200 MHz, $CDCl_3$): δ 9.89 (brs, 1H, NH), 7.41-7.38 (m, 5H, Haro), 6.42 (s, 1H, =CH_2), 5.89 (s, 1H, =CH_2), 5.29 (s, 2H, CH_2Cbz), 3.82 (s, 3H, OCH_3), 3.39 (s, 2H, CH_2).

^{19}F NMR (188 MHz, $CDCl_3$): δ = - 68.59 (s, 3F)

^{13}C NMR (75 MHz, $CDCl_3$): δ = 167.9 (COCbz), 153.3 (CO), 137.6 (q, $^2J_{C,F}$ = 34 Hz, C=N), 135.5 (=C), 132.1 (=CH_2), 130.7 (Caro), 128.5, 128.3, 128.2 (CHaro), 120.8 (q, $^1J_{C,F}$ = 274 Hz, CF_3), 67.8 (CH_2Cbz), 52.9 (OCH_3), 27.8 (CH_2).

HRMS (ESI+TOF) m/z $C_{15}H_{15}F_3N_2O_4$ $[M+Na]^+$ calc. 367.0882, found. 367.0841

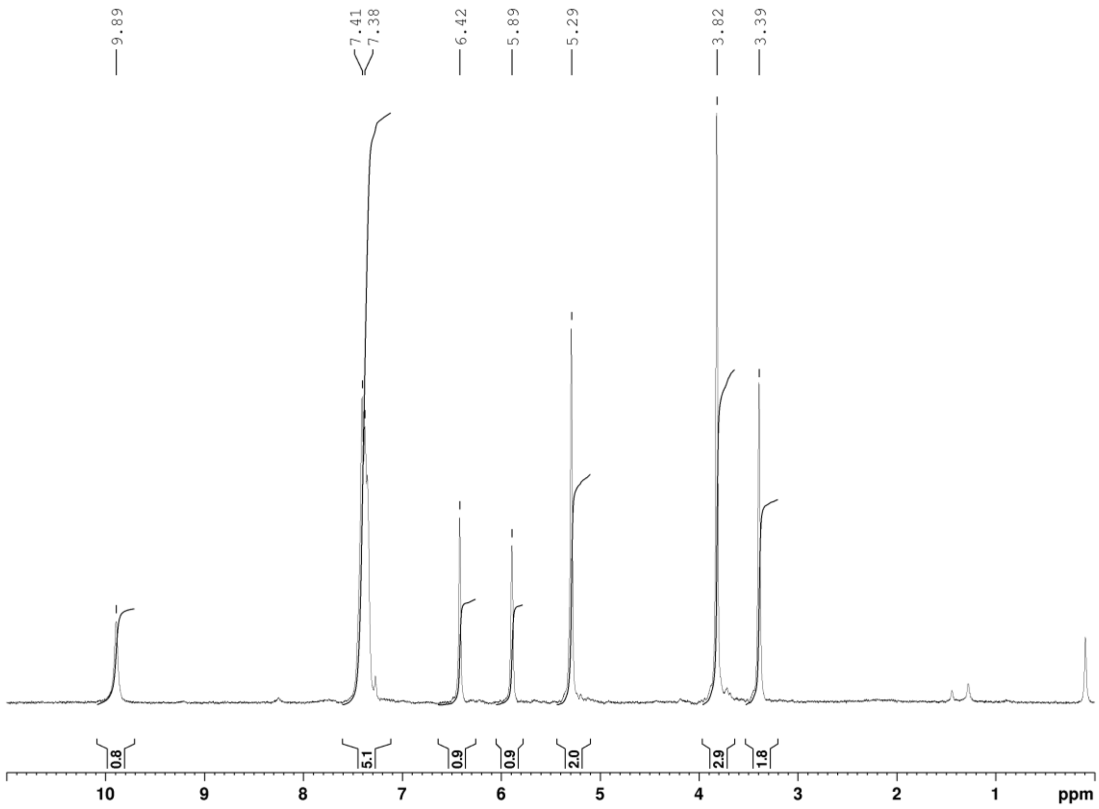

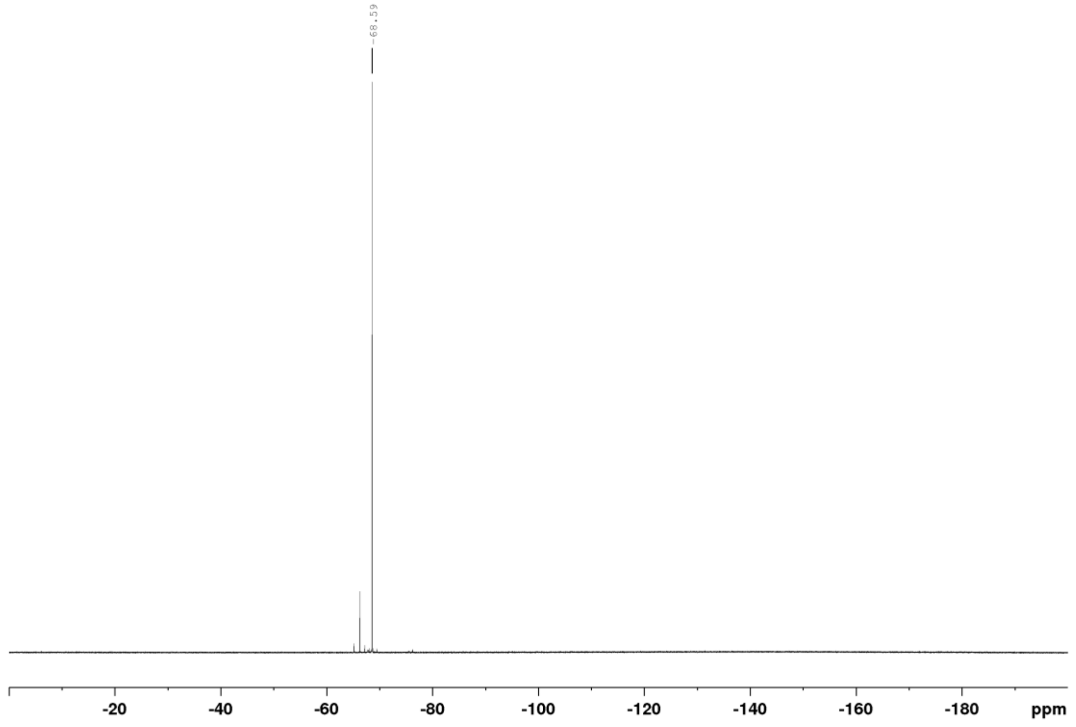
-68.59
-20
-40
-60
-80
-100
-120
-140
-160
-180
ppm

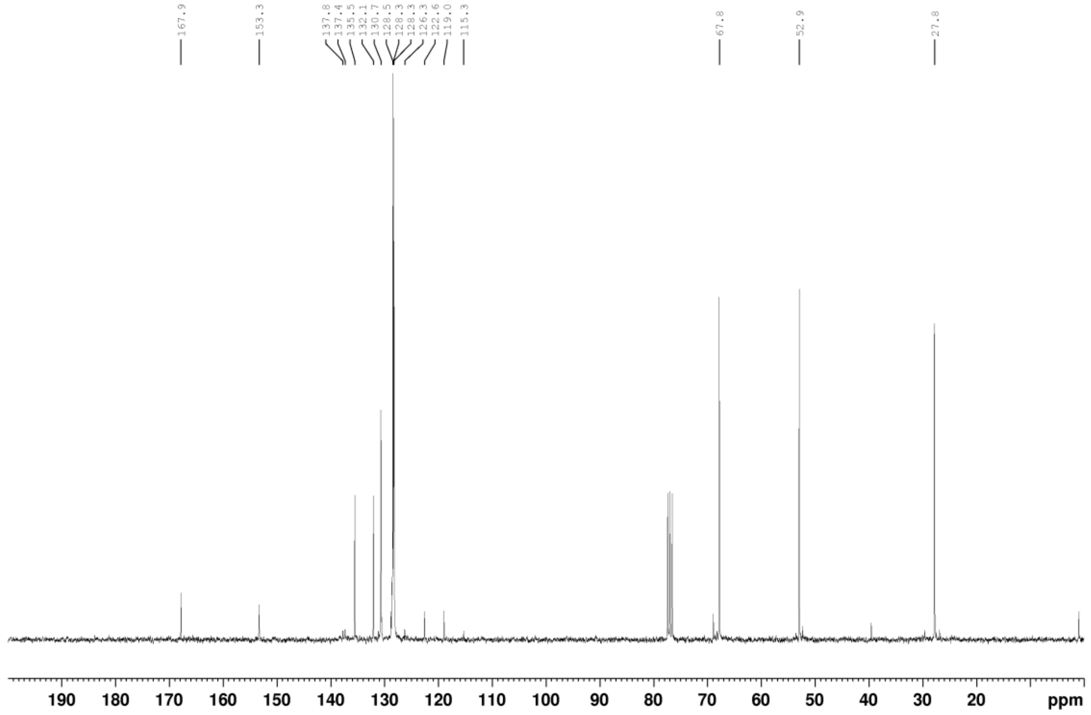
167.9
153.3
137.8
137.4
135.5
132.1
130.7
128.5
128.3
128.3
126.3
122.6
119.0
115.3
67.8
52.9
27.8
190
180
170
160
150
140
130
120
110
100
90
80
70
60
50
40
30
20
ppm

***tert*-Butyl (E)-2-(1,1-difluoro-4-(methoxycarbonyl)pent-4-en-2-ylidene)hydrazine-1-carboxylate (6c)**

The product **6c** is obtained following the general procedure (**C**) as a yellow solid (0.346 g, 69% (I_2)/73% (NBS))

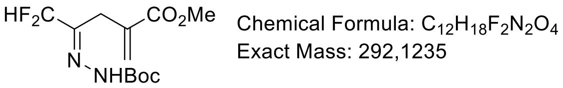

^{1}H NMR (200 MHz, $CDCl_3$): δ= 9.34 (s, 1H, NHBoc), 6.40 (s, 1H, =CH_2), 6.20 (t, $^{2}J_{H,F}$ = 54 Hz, 1H, CF_2H), 5.88 (s, 1H, =CH_2), 3.84 (s, 3H, OCH_3), 3.37 (s, 2H, CH_2), 1.54 (m, 9H, $(CH_3)_3$).

^{19}F NMR (188 MHz, $CDCl_3$): δ = - 116.4 (d, $^{2}J_{H,F}$ = 54 Hz, 2F)

^{13}C NMR (75 MHz, $CDCl_3$): δ = 167.9 (COBoc), 152.4 (CO), 142.0 (t, $^{2}J_{C,F}$ = 29 Hz, C=N), 132.7 (=C), 130.2 (=CH_2), 115.5 (t, $^{1}J_{C,F}$ = 238 Hz, CF_2H), 81.9 (Cq), 52.7 (OCH_3), 28.1 (CH_2), 25.9 (CH_3).

HRMS (ESI+TOF) m/z $C_{12}H_{18}F_2N_2O_4$ $[M+Na]^+$ calc. 315.1132, found. 315.1154

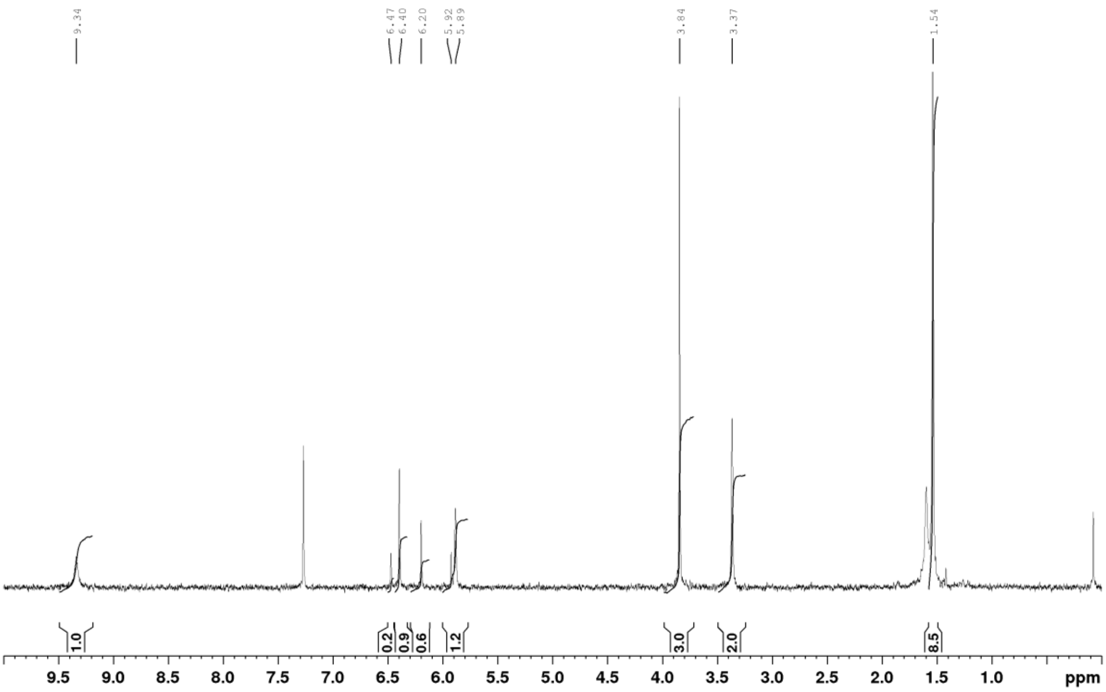

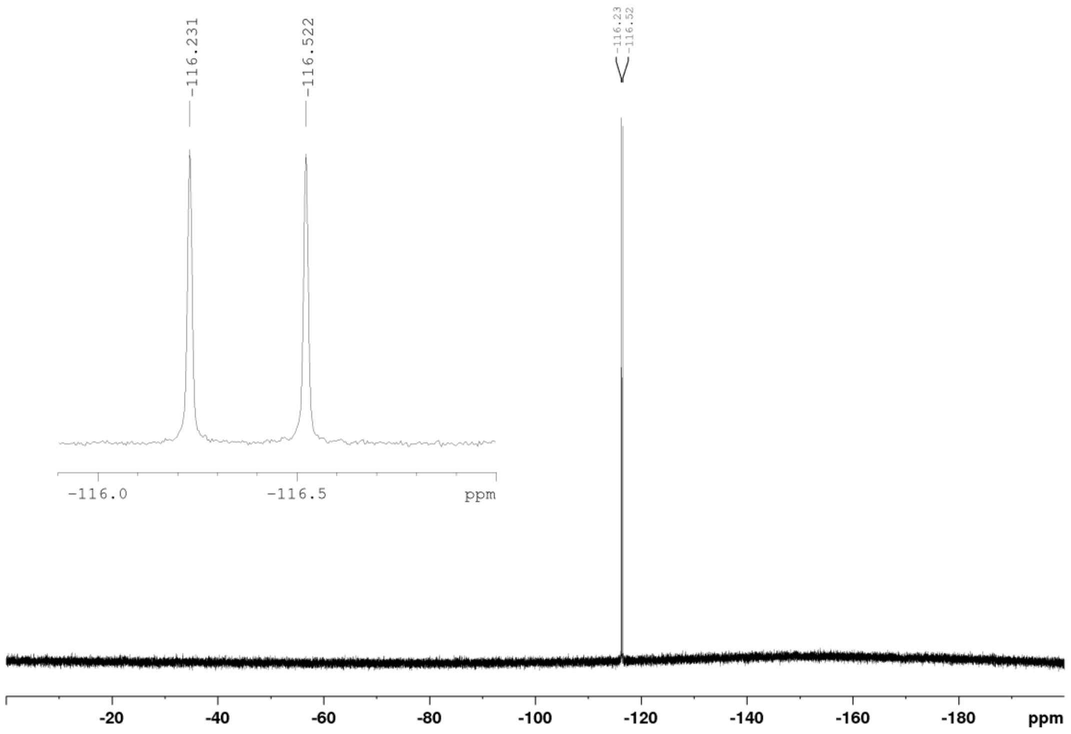

-116.231
-116.522
-116.0
-116.5
ppm
-116.23
-116.52
-20
-40
-60
-80
-100
-120
-140
-160
-180
ppm

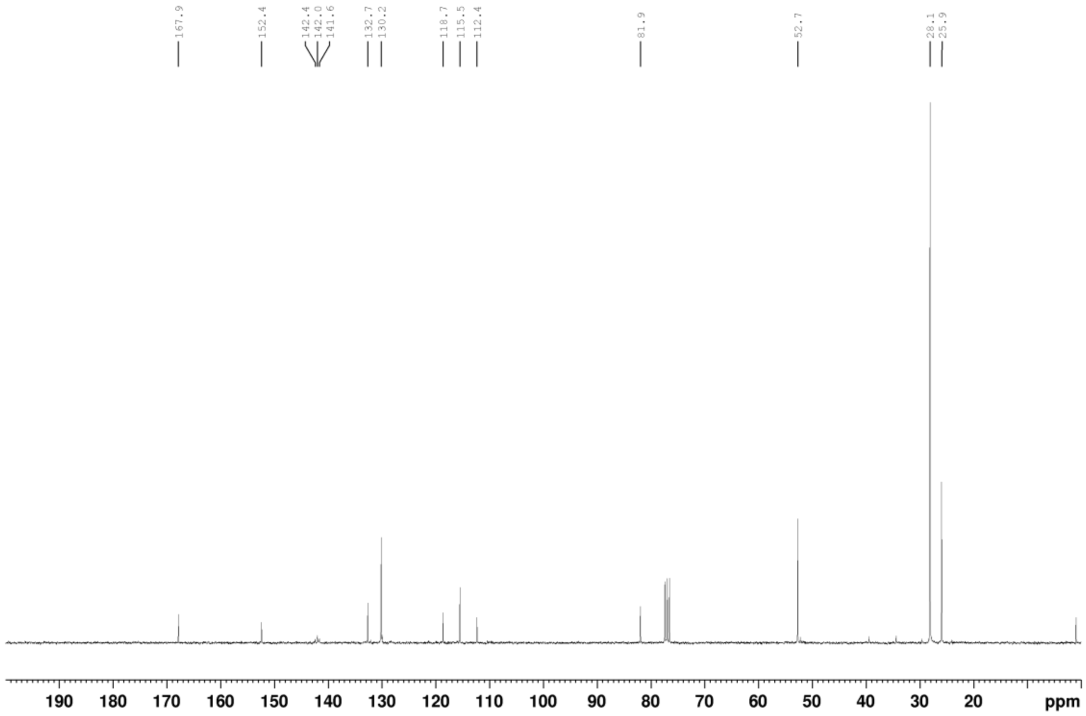

167.9
152.4
142.4
142.0
141.6
132.7
130.2
118.7
115.5
112.4
81.9
52.7
28.1
25.9
190 180 170 160 150 140 130 120 110 100 90 80 70 60 50 40 30 20 ppm

Benzyl (E)-2-(1,1-difluoro-4-(methoxycarbonyl)pent-4-en-2-ylidene)hydrazine-1-carboxylate (6d)

The product **6d** is obtained following the general procedure (**C**) as a yellow oil (0.222 g, 78% (I_2)/76% (NBS))

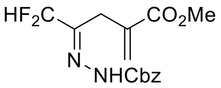

Chemical Formula: $C_{15}H_{16}F_2N_2O_4$
Exact Mass: 326,1078

^{1}H NMR (200 MHz, $CDCl_3$): δ = 9.86 (s, 1H, NHCbz), 7.40-7.37 (m, 5H, Haro), 6.39 (s, 1H, =CH_2), 6.19 (t, $^2J_{H,F}$ = 54 Hz, 1H, CF_2H), 5.92 (s, 1H, =CH_2), 5.27 (s, 2H, CH_2Cbz), 3.80 (s, 3H, OCH_3), 3.36 (s, 2H, CH_2).

^{19}F NMR (188 MHz, $CDCl_3$): δ = - 116.4 (d, $^2J_{H,F}$ = 54 Hz, 2F)

^{13}C NMR (75 MHz, $CDCl_3$): δ = 168.2 (COCbz), 153.6 (CO), 143.2 (t, $^2J_{C,F}$ = 30 Hz, C=N), 135.6 (=C), 132.6 (=CH_2), 130.7 (Caro), 128.5, 128.3, 128.2 (CHaro), 115.3 (t, $^1J_{C,F}$ = 238 Hz, CF_2H), 67.6 (CH_2Cbz), 52.8 (OCH_3), 26.1 (CH_2).

HRMS (ESI+TOF) m/z $C_{15}H_{16}F_2N_2O_4$ $[M+Na]^+$ calc. 349.0976, found. 349.0957

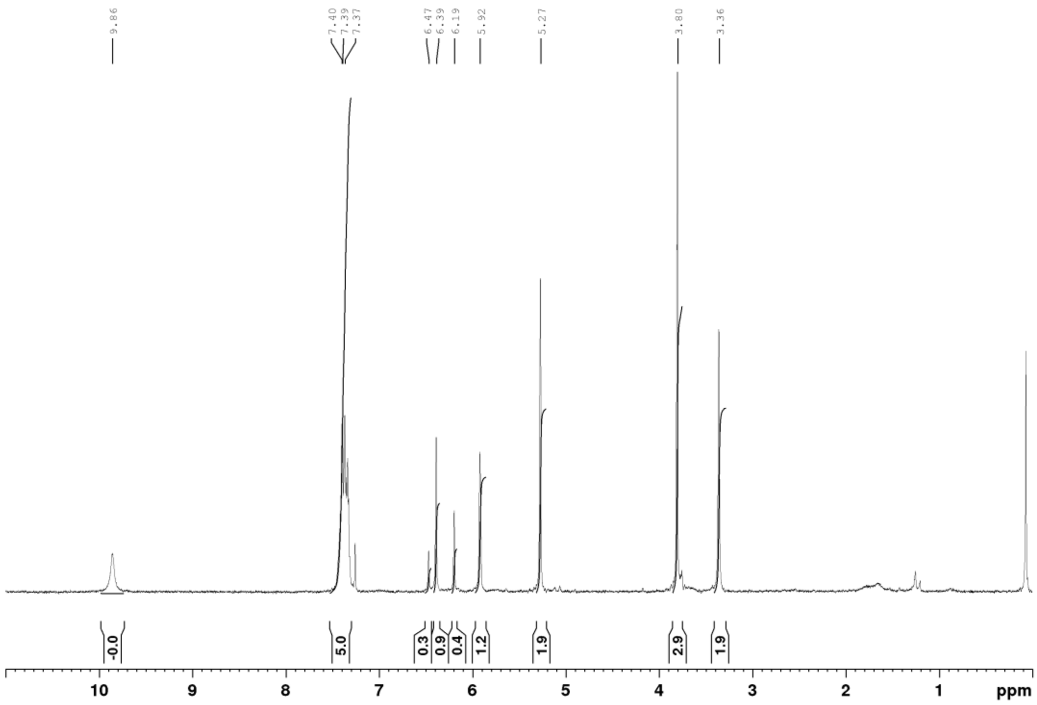

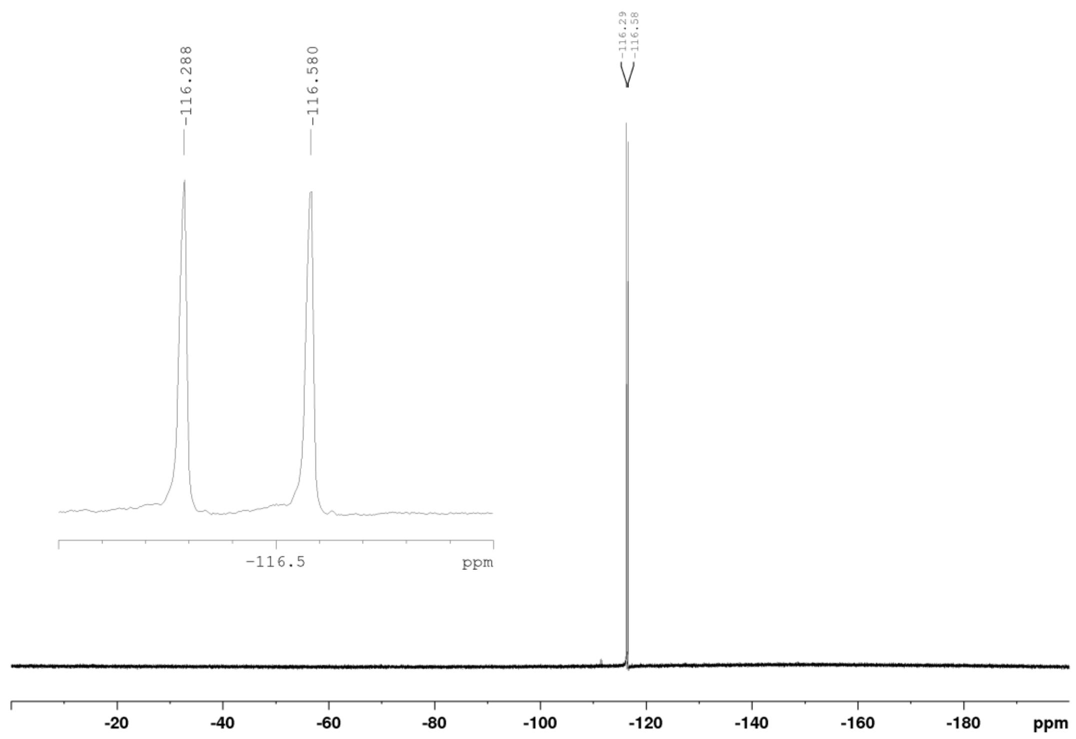

-116.288
-116.580
-116.5
ppm
-116.29
-116.58
-20
-40
-60
-80
-100
-120
-140
-160
-180
ppm

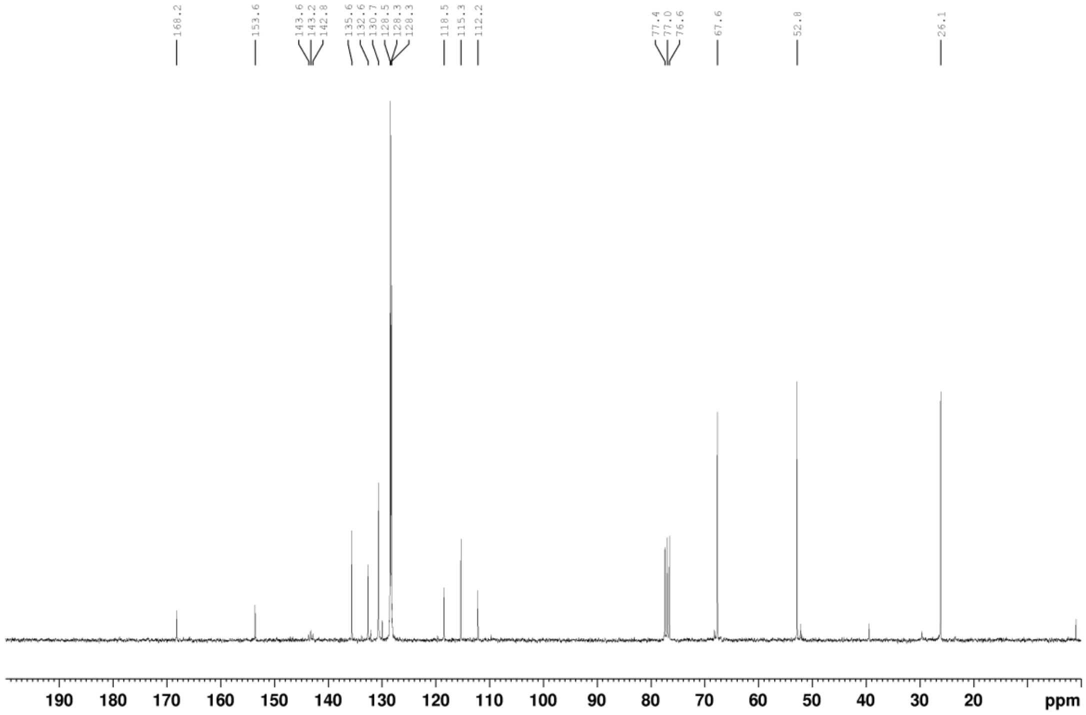

168.2
153.6
143.6
143.2
142.8
135.6
132.6
130.7
128.5
128.3
128.3
118.5
115.3
112.2
77.4
77.0
76.6
67.6
52.8
26.1
190 180 170 160 150 140 130 120 110 100 90 80 70 60 50 40 30 20 ppm

Methyl (E)-4-(2-(((benzyloxy)carbonyl)-L-phenylalanyl)hydrazineylidene)-5,5,5-trifluoro-2-methylenepentanoate (6e)

The product **6e** is obtained following the general procedure (**C**) as a mixture of two conformers (ratio 77/23) as a yellow oil (0.667 g, 75%).

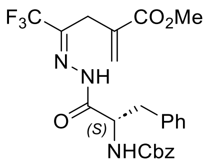

Chemical Formula: $C_{24}H_{24}F_3N_3O_5$
Exact Mass: 491,1668

^{1}H NMR (200 MHz, $CDCl_3$): δ = 10.62 and 10.12 (2brs, 0.2 and 0.8H, NHN), 7.23-7.08 (m, 10H, Haro), 6.26 (s, 1H, =CH_2), 5.75 and 5.48 (2s, 0.2 and 0.8H, NHCbz), 5.58 (d, $^3J_{H,H}$ = 7.6 Hz, 1H, =CH_2), 5.33 (d, $^3J_{H,H}$ = 6.6 Hz, 1H, CHα), 4.98 (s, 2H, CH_2Cbz), 3.71 and 3.69 (s, 3H, OCH_3), 3.21 (d, $^3J_{H,H}$ = 6.6 Hz, 2H, CH_2), 3.03 (dd, $^1J_{H,H}$ = 13.7 Hz, $^3J_{H,H}$ = 5.15 Hz, 1H, CH_2Ph), 2.87 (dd, $^1J_{H,H}$ = 13.2 Hz, $^3J_{H,H}$ = 7.96 Hz, 1H, CH_2Ph).

^{19}F NMR (188 MHz, $CDCl_3$): δ = -68.7(s, 3F), -69.3(s, 3F).

^{13}C NMR (75 MHz, $CDCl_3$): δ = 175.1 (CONH), 167.0 (COCbz), 156.15 and 155.7 (CO), 138.2 (q, $^2J_{C,F}$ = 36 Hz, C=N), 136.4 and 135.9 (=C), 131.6 (=CH_2), 129.5 (Caro), 129.1, 128.7, 128.5, 128.4, 128.1, 127.9, 127.0 (CHaro), 120.6 (q, $^1J_{C,F}$ = 278 Hz, CF_3), 67.1 and 66.8 (CH_2Cbz), 55.6 (CHα), 52.9 and 52.7 (OCH_3), 39.3 and 38.7 (CH_2Ph), 28.0 and 27.6 (CH_2).

HRMS (ESI+TOF) m/z $C_{24}H_{24}F_2N_3O_5$ $[M+H]^+$ calc. 492.1741, found. 492.1748

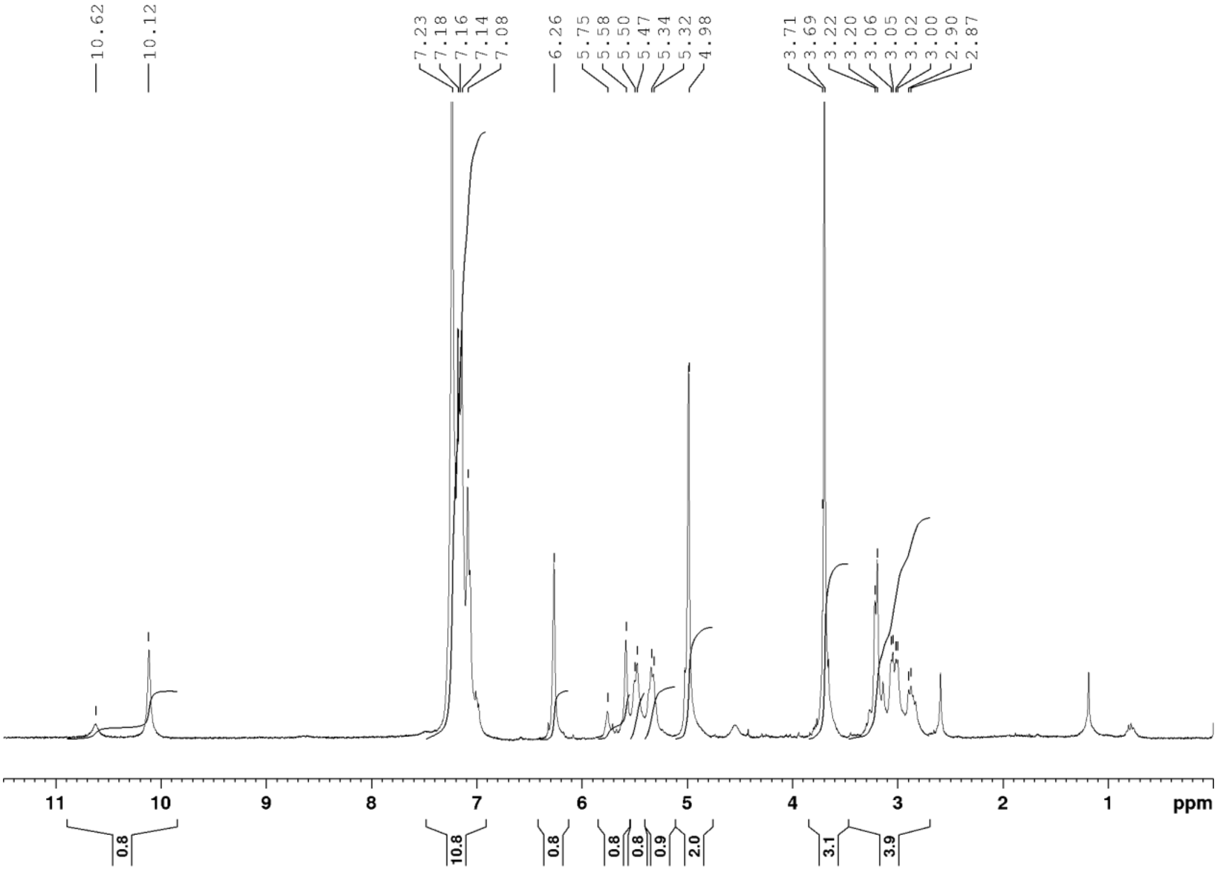

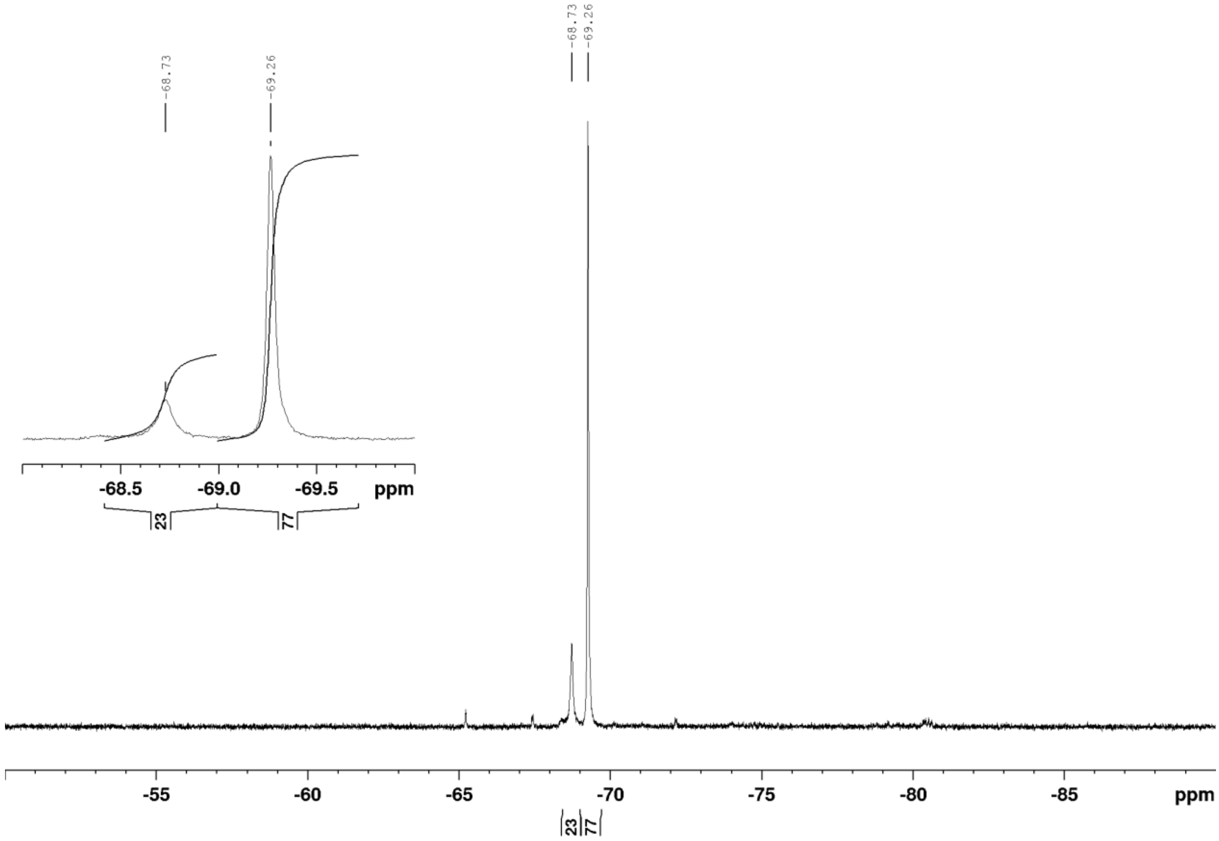
-68.73
-69.26
-68.5
-69.0
-69.5
ppm
23
77
-55
-60
-65
-70
-75
-80
-85
ppm

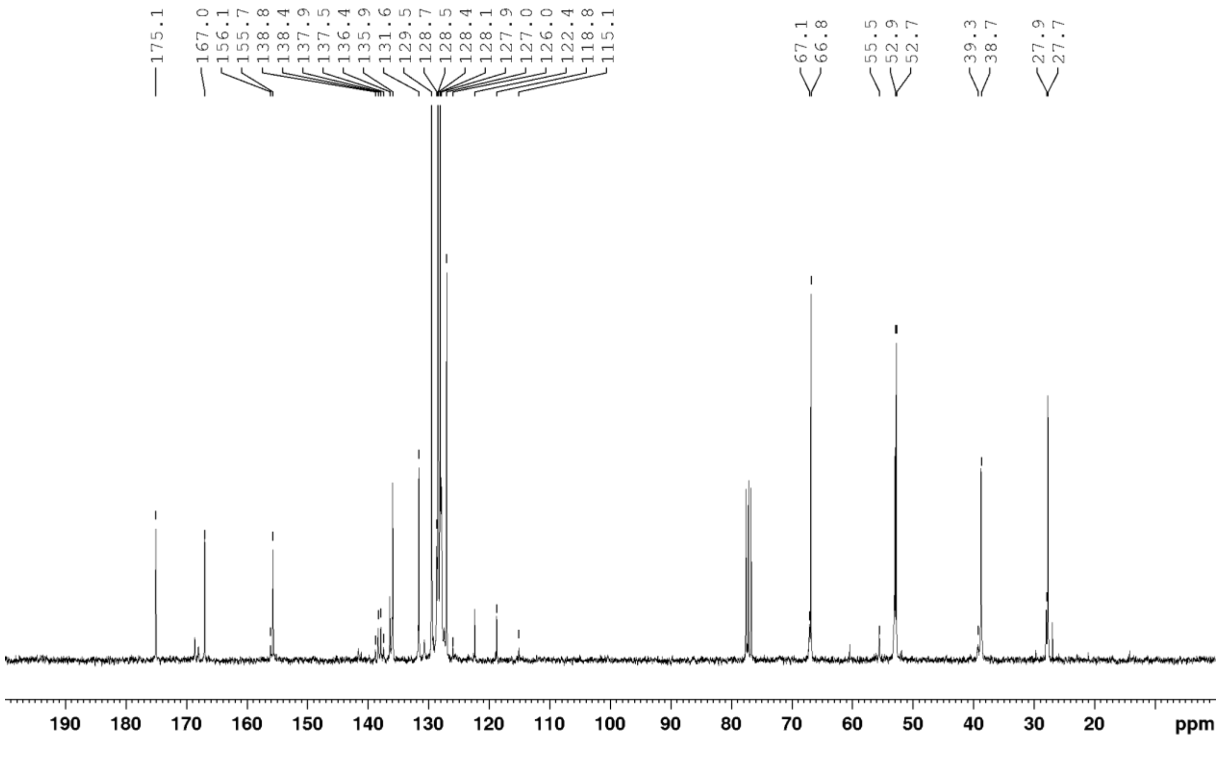
175.1
167.0
156.1
155.7
138.8
138.4
137.9
137.5
136.4
135.9
131.6
129.5
128.7
128.5
128.4
128.1
127.9
127.0
126.0
122.4
118.8
115.1
67.1
66.8
55.5
52.9
52.7
39.3
38.7
27.9
27.7
190
180
170
160
150
140
130
120
110
100
90
80
70
60
50
40
30
20
ppm

Methyl (E)-4-(2-(((benzyloxy)carbonyl)-L-phenylalanyl)hydrazineylidene)-5,5-difluoro-2-methylenepentanoate (6f)

The product **6f** is obtained following the general procedure (**C**) as a mixture of two conformers (ratio 52/48) a yellow oil (0.566 g, 69%).

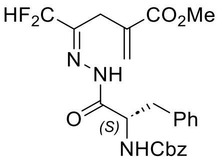

Chemical Formula: $C_{24}H_{25}F_2N_3O_5$
Exact Mass: 473,1762

^{1}H NMR (200 MHz, $CDCl_3$): δ = 10.63 and 10.06 (2s, 0.4 and 0.5H, NHN), 7.23-7.06 (m, 10H, Haro), 6.23 (d, $^{3}J_{H,H}$ = 11.28 Hz,1H, =CH_2), 6.11 (t, $^{2}J_{H,F}$ = 54 Hz, 0.5H, CF_2H), 5.91 (t, $^{2}J_{H,F}$ = 54 Hz, 0.5H, CF_2H), 5.78 (s, 0.5H, NHCbz), 5.61 (d, $^{3}J_{H,H}$ = 8.52 Hz, 0.5H, NHCbz), 5.53 (s, 1H, =CH_2), 5.33 (m, 0.6H, CHα), 4.99 (s, 2H), 4.52 (m, 0.4H, CHα), 3.67 (s, 3H, OCH_3), 3.15 (d, $^{2}J_{H,H}$ = 12.2 Hz, 2H, CH_2), 3.05 (d, $^{3}J_{H,H}$ = 6.81 Hz, 1H, CH_2Ph), 2.96 (d, $^{3}J_{H,H}$ = 7.11 Hz, 1H, CH_2Ph).

^{19}F NMR (188 MHz, $CDCl_3$): δ = -116.63 (d, $^{2}J_{H,F}$ = 54 Hz, 2F), -116.59 (d, $^{2}J_{H,F}$ = 54 Hz, 2F).

^{13}C NMR (75 MHz, $CDCl_3$): δ = 174.2 (CONH), 168.7 and 167.5 (COCbz), 155.7 (CO), 146.9 (t, $^{2}J_{C,F}$ = 29.6 Hz, C=N), 143.3 (t, $^{2}J_{C,F}$ = 29.1 Hz, C=N), 136.4 and 136.0 (=C), 131.1 and 129.4 (=CH_2), 129.2 and 129.0 (Caro), 128.7, 128.5, 128.3, 128.2, 128.1, 128.0, 127.0 (CHaro), 115.2 (t, $^{1}J_{C,F}$ = 239.4 Hz, CF_2H), 114.7 (t, $^{1}J_{C,F}$ = 239.4 Hz, CF_2H), 67.1 and 66.8 (CH_2Cbz), 55.6 (CHα), 53.0 and 52.7 (OCH_3), 39.1 and 38.8 (CH_2Ph), 26.3 and 26.1 (CH_2).

HRMS (ESI+TOF) m/z $C_{24}H_{25}F_2N_3O_5$ $[M+Na]^+$ calc. 496.1654, found. 496.1662

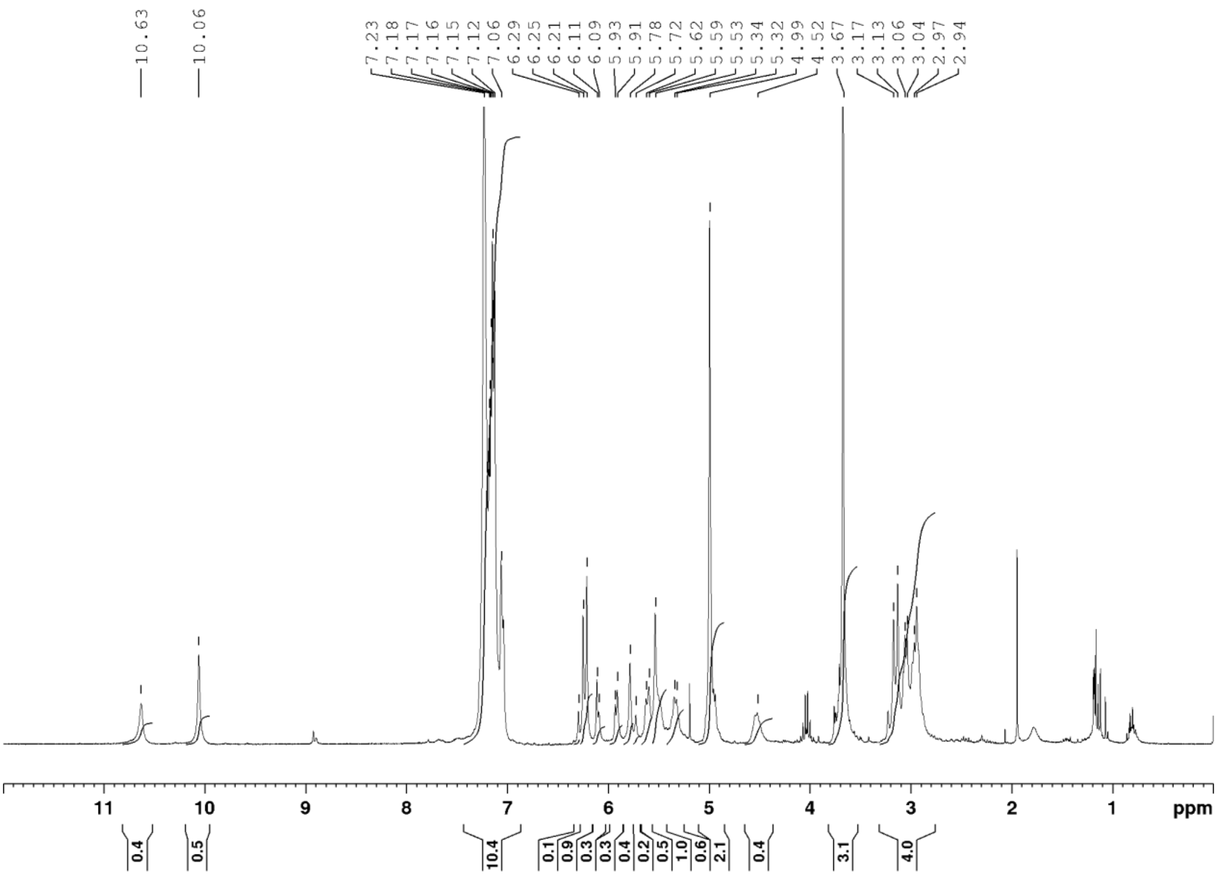

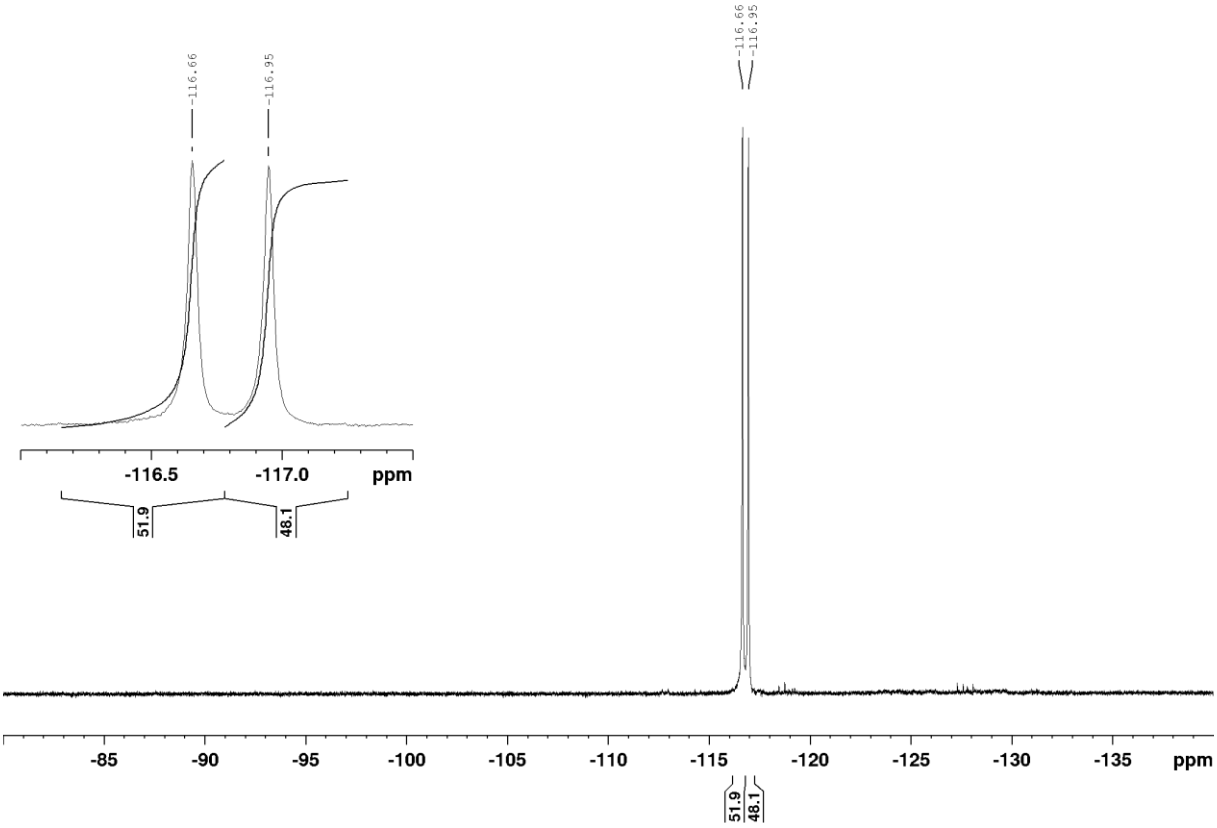
-116.66
-116.95
-116.5
-117.0
ppm
51.9
48.1
-85
-90
-95
-100
-105
-110
-115
-120
-125
-130
-135
ppm

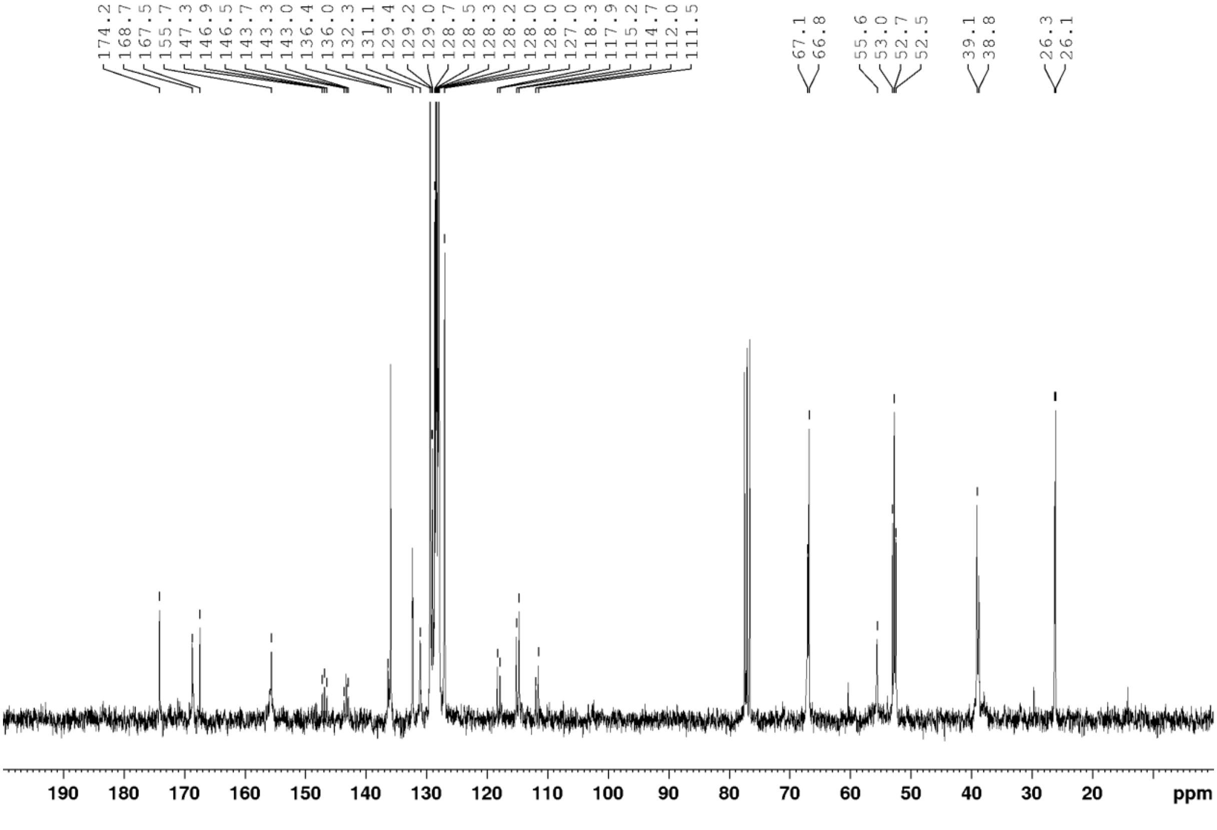
174.2
168.7
167.5
155.7
147.3
146.9
146.5
143.7
143.3
143.0
136.4
136.0
132.3
131.1
129.4
129.2
129.0
128.7
128.5
128.3
128.2
128.0
127.0
118.3
117.9
115.2
114.7
112.0
111.5
67.1
66.8
55.6
53.0
52.7
52.5
39.1
38.8
26.3
26.1
190
180
170
160
150
140
130
120
110
100
90
80
70
60
50
40
30
20
ppm

d) <u>Tetrahydropyridazines 7a–f</u>

1-(tert-Butyl) 5-methyl 3-(trifluoromethyl)-3,4-dihydropyridazine-1,5(2H)-dicarboxylate (7a)

The product **7a** is obtained following the general procedure **D** as a yellow oil (0.186 g, 63%).

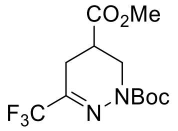

Chemical Formula: $C_{12}H_{17}F_3N_2O_4$
Exact Mass: 310,1140

^{1}H NMR (200 MHz, $CDCl_3$): δ= 4.16 (dd, $^{2}J_{H,H}$ = 13 Hz, $^{3}J_{H,H}$ = 4 Hz, 1H, CH_2NBoc), 3.73 (s, 3H, OCH_3), 3.50 (dd, $^{2}J_{H,H}$ = 13 Hz, $^{3}J_{H,H}$ = 9 Hz, 1H, CH_2NBoc), 2.92-2.84 (m, 1H, $CHCO_2$Me), 2.57-2.54 (m, 2H, CH_2), 1.50 (s, 9H, $(CH_3)_3$)

^{19}F NMR (188 MHz, $CDCl_3$): δ = - 71.10 (s, 3F).

^{13}C NMR (75 MHz, $CDCl_3$): δ = 170.4 (COBoc), 151.0 (CO), 135.0 (d, $^{2}J_{C,F}$ = 34 Hz, C=N), 118.0 (q, $^{1}J_{C,F}$ = 271 Hz, CF_3), 82.1 (Cq), 51.6 (OCH_3), 41.6 (CH_2NBoc), 32.6 (CH), 27.1 (CH_3), 21.6 (CH_2).

HRMS (ESI+TOF) m/z $C_{12}H_{17}F_3N_2O_4$ $[M+Na]^+$ calc. 333.1038, found. 333.1086

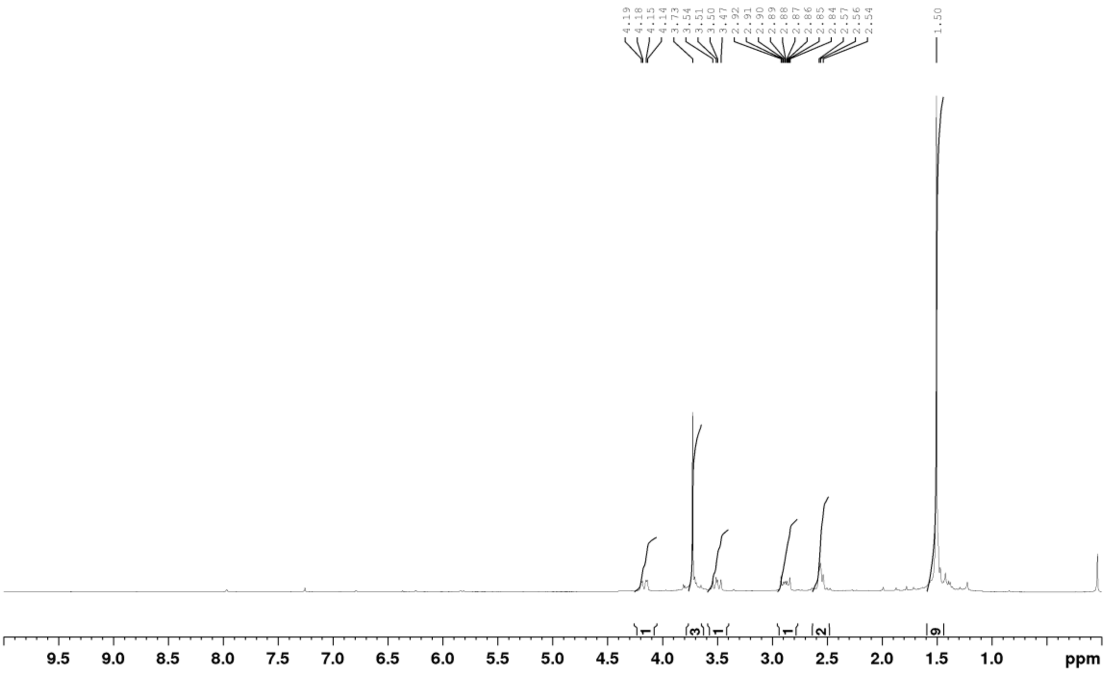

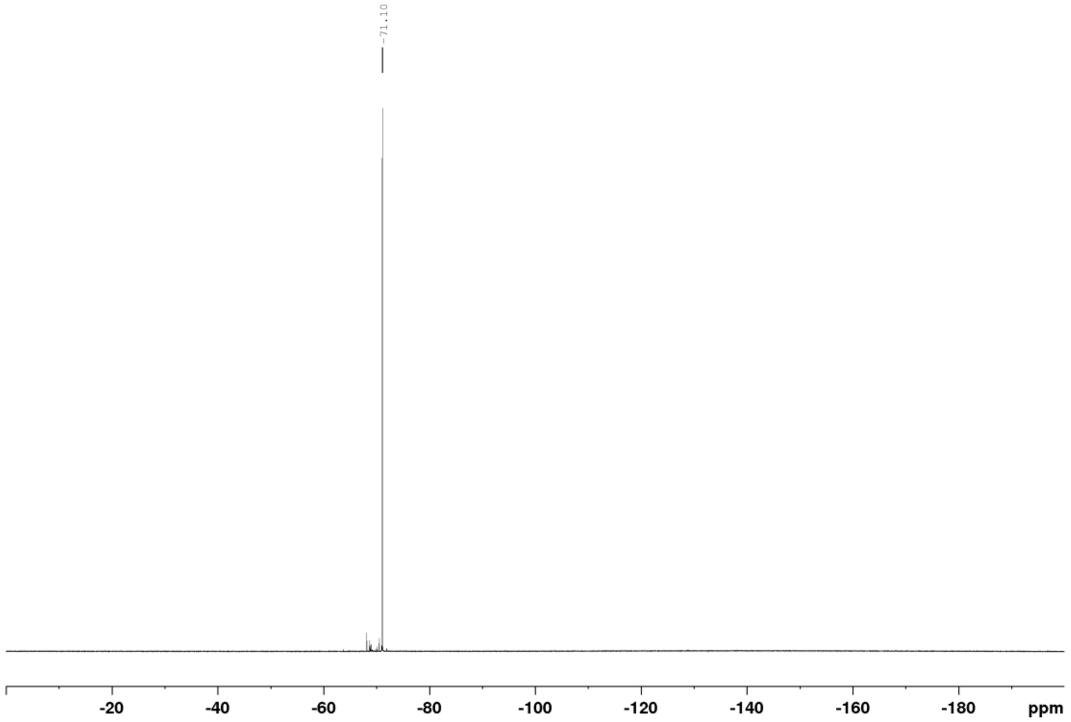
-71.10
-20
-40
-60
-80
-100
-120
-140
-160
-180
ppm

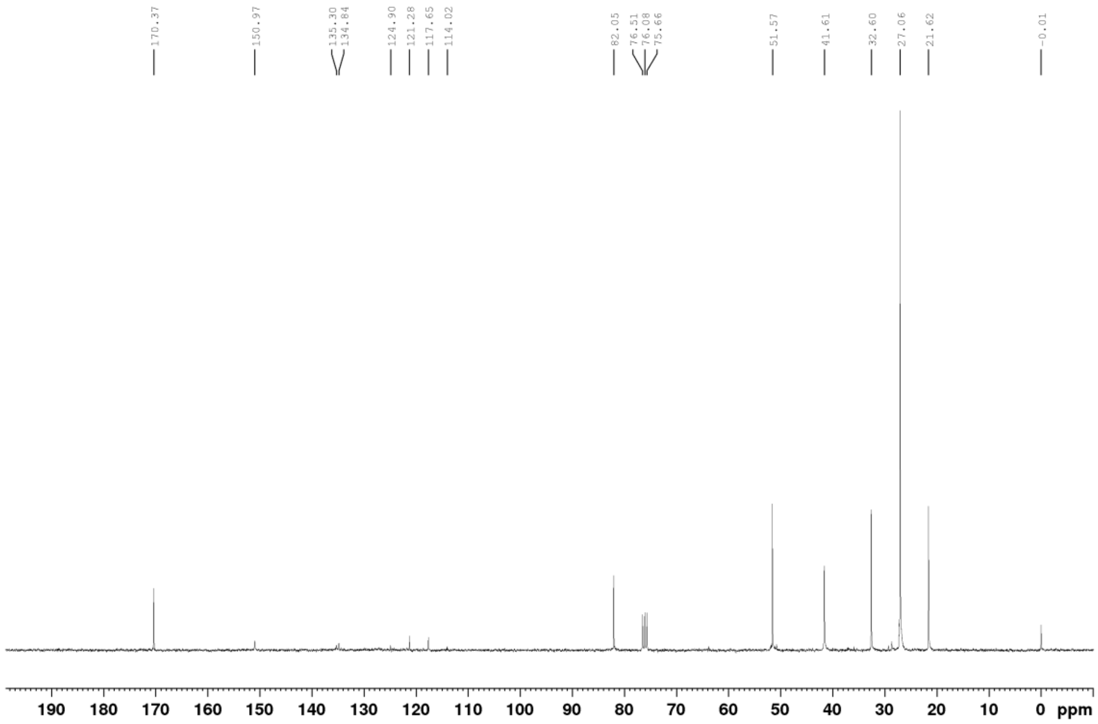
170.37
150.97
135.30
134.84
124.90
121.28
117.65
114.02
82.05
76.51
76.08
75.66
51.57
41.61
32.60
27.06
21.62
-0.01
190
180
170
160
150
140
130
120
110
100
90
80
70
60
50
40
30
20
10
0
ppm

1-Benzyl 5-methyl 3-(trifluoromethyl)-5,6-dihydropyridazine-1,5(4H)-dicarboxylate (7b)

The product **7b** is obtained following the general procedure **D** as a yellow oil (0.396 g, 94%)

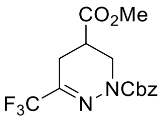

Chemical Formula: $C_{15}H_{15}F_3N_2O_4$
Exact Mass: 344,0984

^{1}H NMR (200 MHz, $CDCl_3$): δ = 7.42-7.31 (m, 5H, Haro), 5.32 (d, $^2J_{H,H}$ = 12 Hz, 1H, CH_2Cbz), 5.27 (d, $^2J_{H,H}$ = 12 Hz, 1H, CH_2Cbz), 4.22 (dd, $^2J_{H,H}$ = 13 Hz, $^3J_{H,H}$ = 4.0 Hz, 1H, CH_2NCbz), 3.73 (s, 3H, OCH_3), 3.62 (dd, $^2J_{H,H}$ = 13 Hz, $^3J_{H,H}$ = 9 Hz, 1H, CH_2NCbz), 2.95 (m, 1H, CH), 2.62 (d, $^3J_{H,H}$ = 7.3 Hz, 2H, CH_2).

^{19}F NMR (188 MHz, $CDCl_3$): δ = - 71.12 (s, 3F)

^{13}C NMR (75 MHz, $CDCl_3$): δ = 171.1 (COCbz), 153.4 (CO), 137.3 (q, $^2J_{C,F}$ = 35 Hz, C=N), 135.6 (Caro), 128.6, 128.4, 128.1 (CHaro), 118 (q, $^1J_{C,F}$ = 271 Hz, CF_3), 68.7 (CH_2Cbz), 52.6 (OCH_3), 42.8 (CH_2NBoc), 33.4 (CH), 22.6 (CH_2).

HRMS (ESI+TOF) m/z $C_{15}H_{15}F_3N_2O_4$ $[M+Na]^+$ calc. 367.0882, found. 367.0841

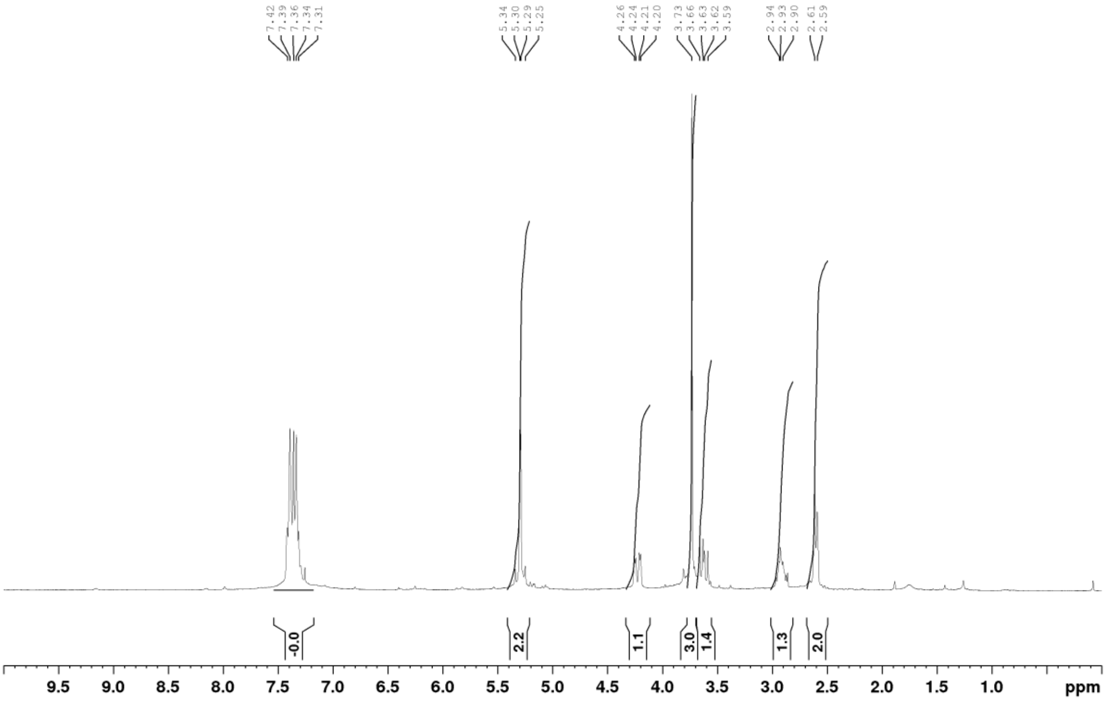

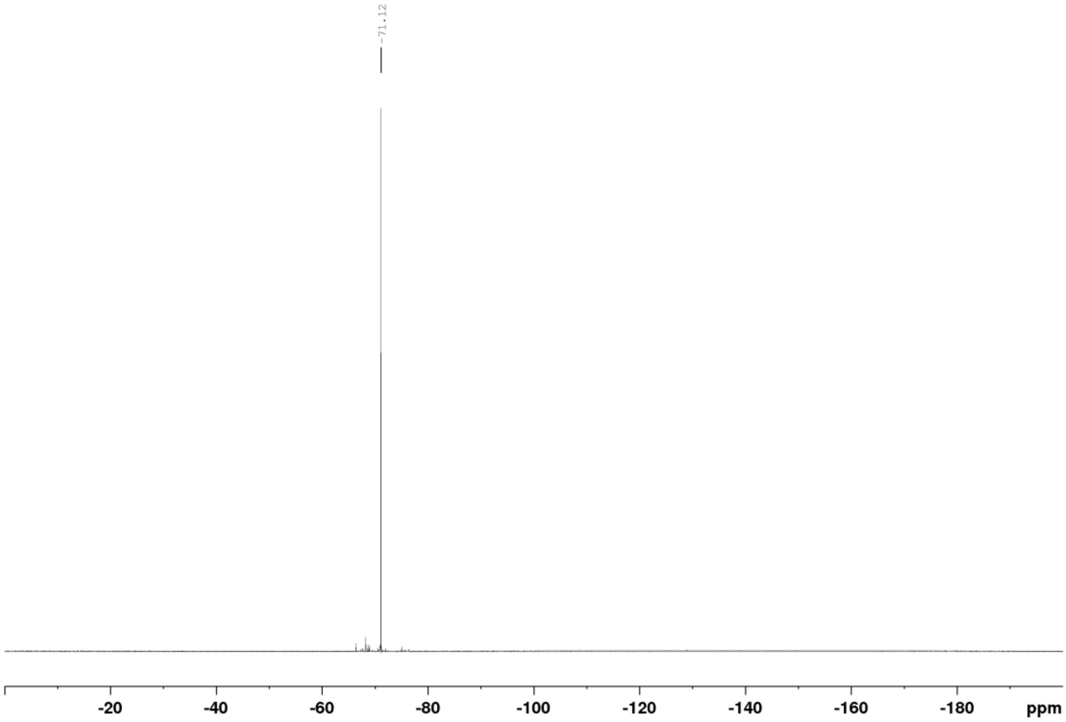

-71.12
-20
-40
-60
-80
-100
-120
-140
-160
-180
ppm

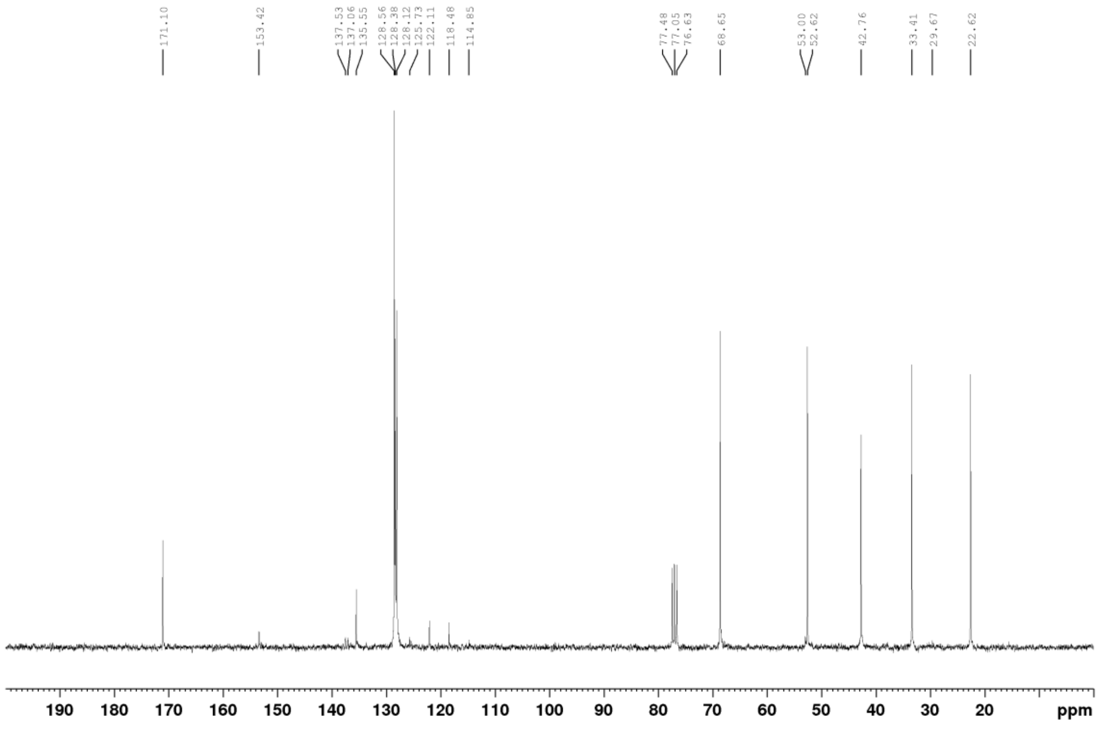

171.10
153.42
137.53
137.06
135.55
128.56
128.38
128.12
125.73
122.11
118.48
114.85
77.48
77.05
76.63
68.65
53.00
52.62
42.76
33.41
29.67
22.62
190
180
170
160
150
140
130
120
110
100
90
80
70
60
50
40
30
20
ppm

1-(tert-Butyl) 5-methyl 3-(difluoromethyl)-3,4-dihydropyridazine-1,5(2H)-dicarboxylate (7c)

The product **7c** is obtained following the general procedure **D** as an orange oil (0.286 g, 83%)

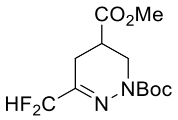

Chemical Formula: $C_{12}H_{18}F_2N_2O_4$
Exact Mass: 292,1235

^{1}H NMR (200 MHz, $CDCl_3$): δ= 6.04 (t, $^2J_{H,F}$ = 54 Hz, 1H, CF_2H), 4.13 (ddd, $^2J_{H,H}$ = 13 Hz, $^2J_{H,H}$ = 2 Hz, $^3J_{H,H}$ = 7 Hz, 1H, CH_2NBoc), 3.69 (s, 3H, OCH_3), 3.44 (dd, $^2J_{H,H}$ = 12 Hz, $^3J_{H,H}$ = 7Hz, 1H, CH_2NBoc), 2.81 (m, 1H, CH), 2.58 (dd, $^2J_{H,H}$ = 18 Hz, $^3J_{H,H}$ = 6 Hz, 1H, CH_2), 2.45 (dd, $^2J_{H,H}$ = 18 Hz, $^3J_{H,H}$ = 9 Hz, 1H, CH_2), 1.47 (m, 9H, $(CH_3)_3$).

^{19}F NMR (188 MHz, $CDCl_3$): δ = -117.5 (dd, $^2J_{F,F}$ = 317 Hz, $^2J_{H,F}$ = 54 Hz, 1F), - 120.3 (dd, $^2J_{F,F}$ = 317 Hz, $^2J_{H,F}$ = 54 Hz, 1F)

^{13}C NMR (75 MHz, $CDCl_3$): δ = 171.4 (COBoc), 151.9 (CO), 142.0 (t, $^2J_{C,F}$ = 31 Hz, C=N), 114.2 (t, $^1J_{C,F}$ = 236 Hz, CF_2H), 82.5 (Cq), 52.2 (OCH_3), 42.7 (CH_2NBoc), 33.3 (CH), 27.8 (CH_3), 20.6 (CH_2).

HRMS (ESI+TOF) m/z $C_{12}H_{18}F_2N_2O_4$ $[M+Na]^+$ calc. 315.1132, found. 315.1165

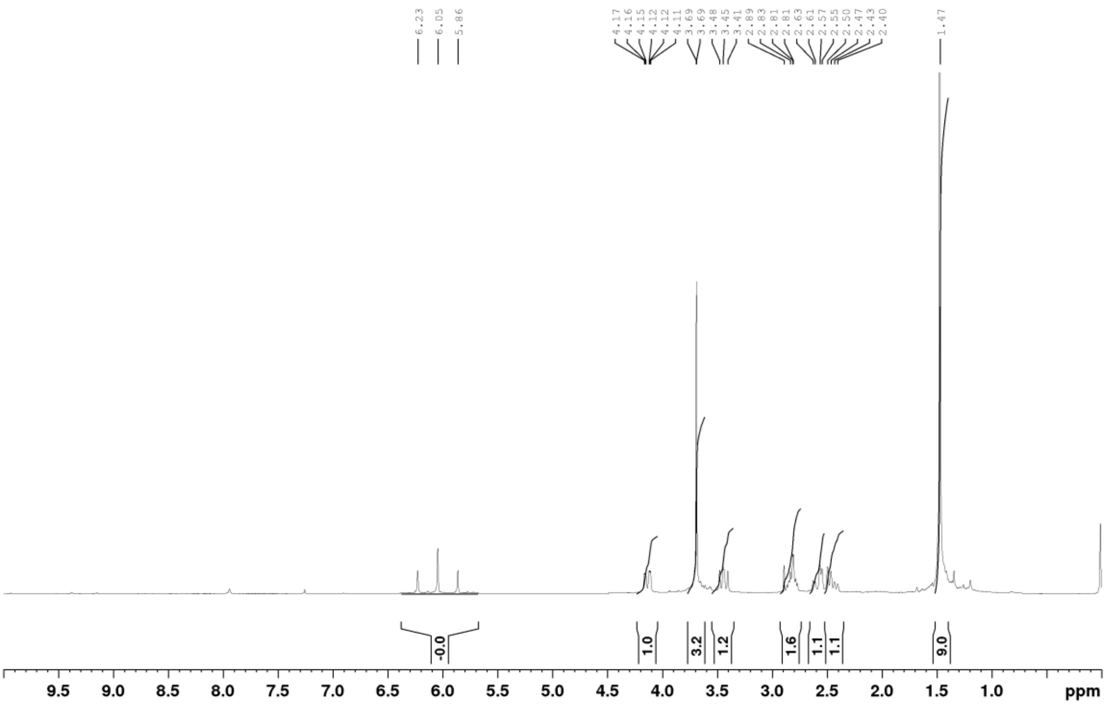

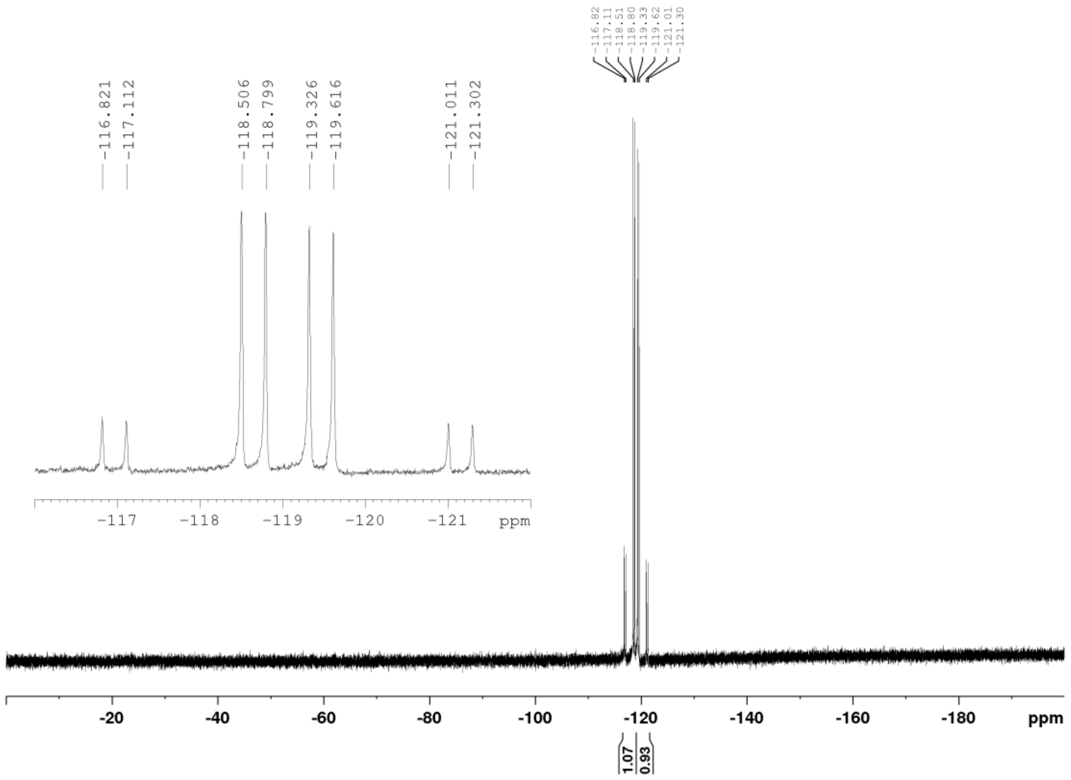
-116.821
-117.112
-118.506
-118.799
-119.326
-119.616
-121.011
-121.302
-117
-118
-119
-120
-121
ppm
-116.82
-117.11
-118.51
-118.80
-119.33
-119.62
-121.01
-121.30
-20
-40
-60
-80
-100
-120
-140
-160
-180
ppm
1.07
0.93

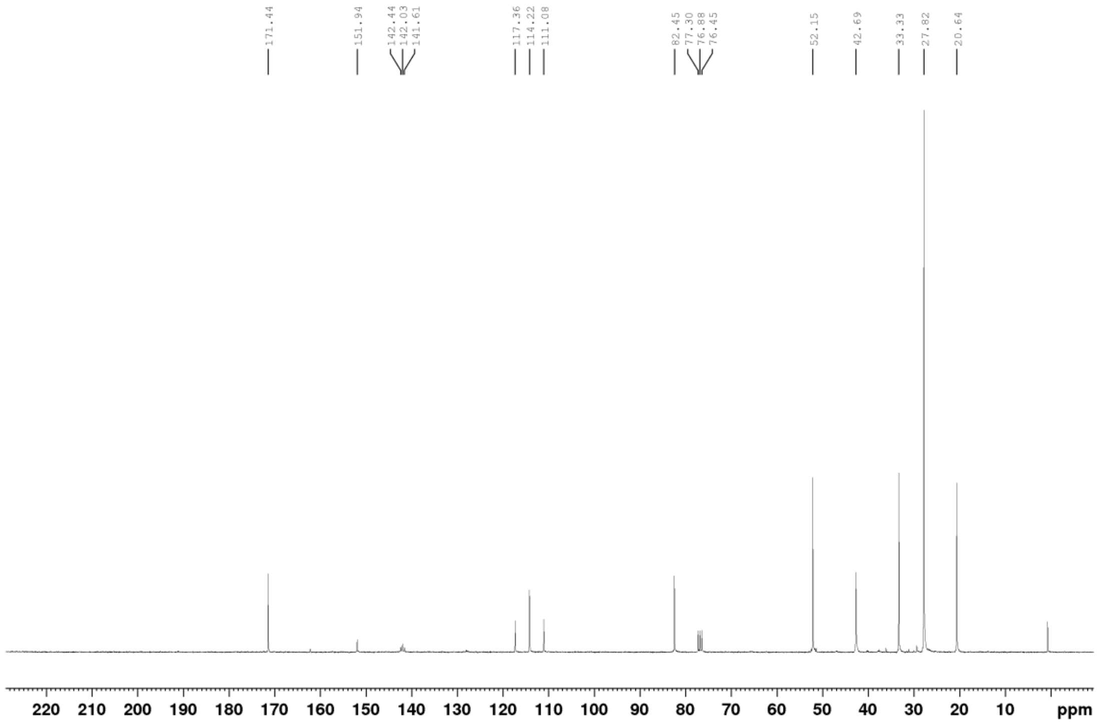
171.44
151.94
142.44
142.03
141.61
117.36
114.22
111.08
82.45
77.30
76.88
76.45
52.15
42.69
33.33
27.82
20.64
220 210 200 190 180 170 160 150 140 130 120 110 100 90 80 70 60 50 40 30 20 10 ppm

1-Benzyl 5-methyl 3-(difluoromethyl)-5,6-dihydropyridazine-1,5(4H)-dicarboxylate (7d)

The product **7d** is obtained following the general procedure **D** an orange oil (0.135 g, 79%)

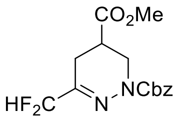

Chemical Formula: $C_{15}H_{16}F_2N_2O_4$
Exact Mass: 326,1078

^{1}H NMR (200 MHz, $CDCl_3$): δ = 7.38-7.33 (m, 5H, Haro)), 6.11 (t, $^2J_{H,F}$ = 54 Hz, 1H, CF_2H), 5.27 (s, 2H, CH_2Cbz), 4.23 (dd, $^2J_{H,H}$ = 12 Hz, $^3J_{H,H}$ = 4 Hz, 1H, CH_2NCbz), 3.72 (s, 3H, OCH_3), 3.60 (dd, $^2J_{H,H}$ = 12 Hz, $^3J_{H,H}$ = 9 Hz, 1H, CH_2NCbz), 2.60 (m, 1H, CH), 2.87 (m, 2H, CH_2),

^{19}F NMR (188 MHz, $CDCl_3$): δ = -118.0 (dd, $^2J_{H,F}$ = 54 Hz, $^2J_{F,F}$ = 318 Hz, 1F), -120.5 (dd, $^2J_{H,F}$ = 54 Hz, $^2J_{F,F}$ = 318 Hz, 1F).

^{13}C NMR (75 MHz, $CDCl_3$): δ = 170.5 (COCbz), 152.6 (CO), 142.4 (t, $^2J_{C,F}$ = 30 Hz, C=N), 134.6 (Caro), 127.6, 124.4, 127.3 (CHaro), 113.2 (t, $^1J_{C,F}$ = 237 Hz, CF_2H), 67.6 (CH_2Cbz), 51.5 (OCH_3) , 42.1 (CH_2NCbz), 32.4 (CH), 20.0 (CH_2).

HRMS (ESI+TOF) m/z $C_{15}H_{16}F_2N_2O_4$ $[M+Na]^+$ calc. 349.0976, found. 349.0942

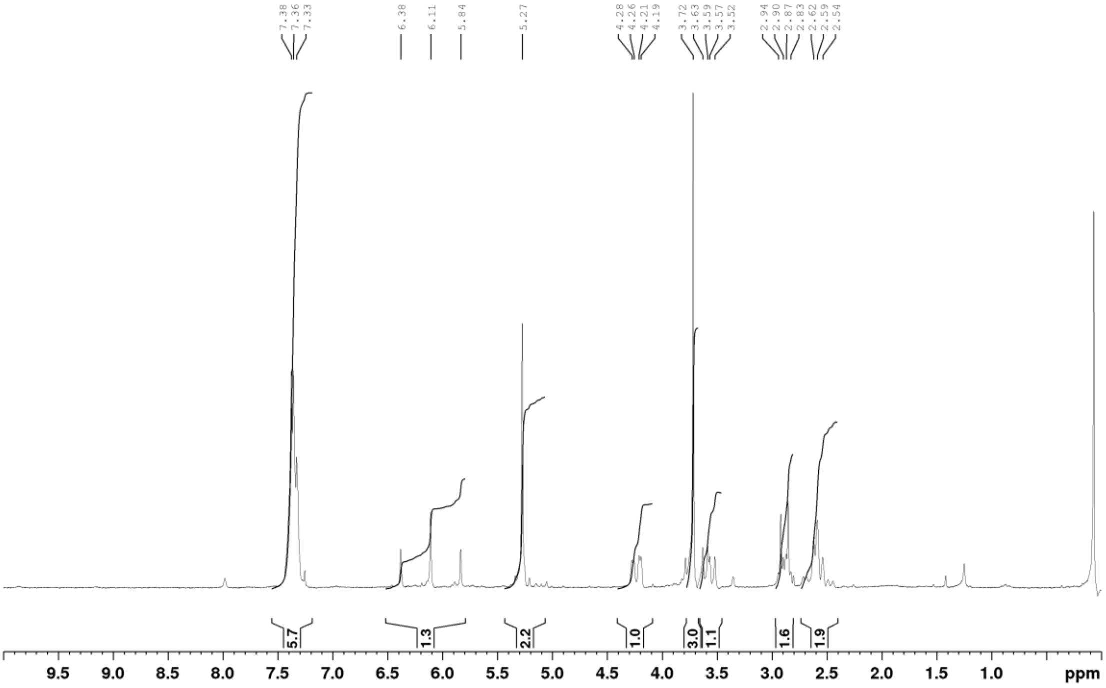

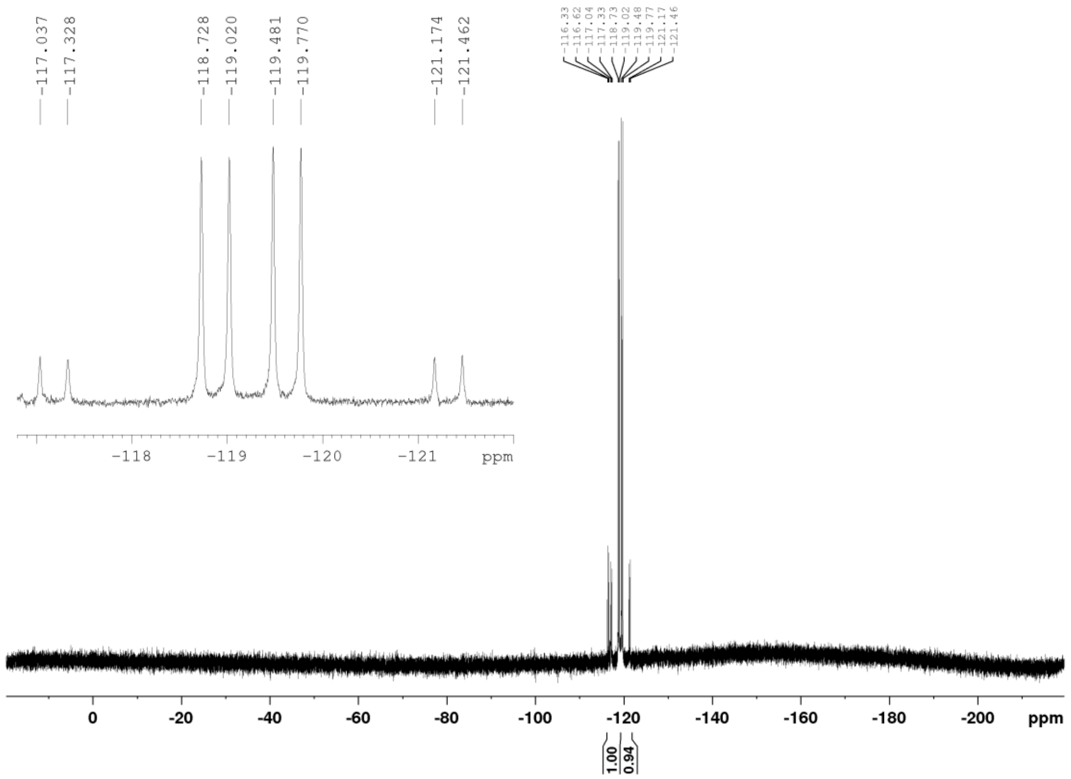
-117.037
-117.328
-118.728
-119.020
-119.481
-119.770
-121.174
-121.462
-118
-119
-120
-121
ppm
-116.33
-116.62
-117.04
-117.33
-118.73
-119.02
-119.48
-119.77
-121.17
-121.46
0
-20
-40
-60
-80
-100
-120
-140
-160
-180
-200
ppm
1.00
0.94

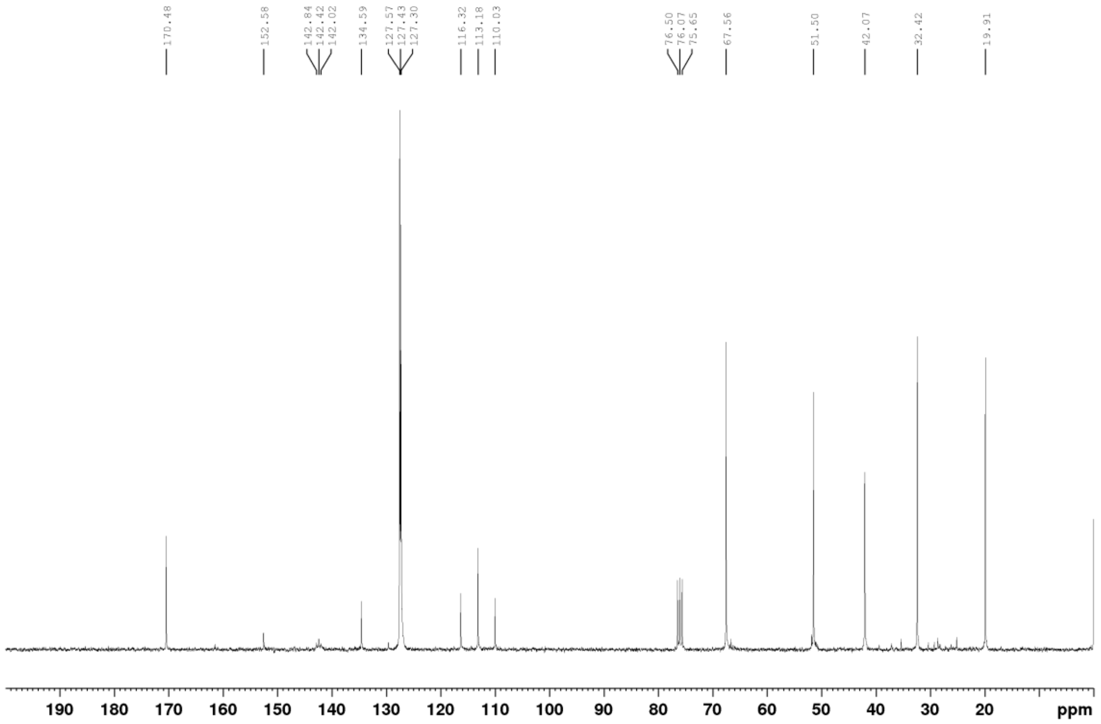
170.48
152.58
142.84
142.42
142.02
134.59
127.57
127.43
127.30
116.32
113.18
110.03
76.50
76.07
75.65
67.56
51.50
42.07
32.42
19.91
190
180
170
160
150
140
130
120
110
100
90
80
70
60
50
40
30
20
ppm

Methyl 2-(((benzyloxy)carbonyl)-L-phenylalanyl)-6-(trifluoromethyl)-2,3,4,5-tetrahydropyridazine-4-carboxylate (7e)

The product **7e** is obtained following the general procedure **D** as a yellow oil (0.480g, 78%).

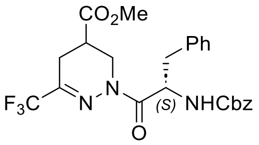

Chemical Formula: $C_{24}H_{24}F_{3}N_{3}O_{5}$
Exact Mass: 491,1668

Methyl (*S*)-2-(((benzyloxy)carbonyl)-L-phenylalanyl)-6-(trifluoromethyl)-2,3,4,5-tetrahydropyridazine-4-carboxylate (7e)

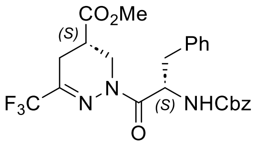

^{1}H NMR (200 MHz, $CDCl_3$): δ= 7.23-7.02 (m, 10H, H_{aro}), 5.48 (m, 2H, NHCbz and CHα), 4.98 (s, 2H, CH_2Cbz), 4.24 (d, $^{3}J_{H,H}$ = 8.2 Hz, 1H, CH_2N), 3.66 (s, 3H, OCH_3), 3.27 (t, $^{3}J_{H,H}$ = 6.4 Hz, 1H, CH_2N), 2.91 (s, 2H, CH_2Ph), 2.49 (m, 1H, CH), 2.44 (m, 2H, CH_2).

^{19}F NMR (188 MHz, $CDCl_3$): δ = - 71.26 (s, 3F).

^{13}C NMR (75 MHz, $CDCl_3$): δ = 172.8 (CON), 170.9 (COCbz), 155.6 (CO), 137.6 (q, $^{2}J_{C,F}$ = 36.7 Hz, C=N), 136.4 and 136.0 (Caro), 129.4, 128.5, 128.4, 128.1, 126.9 (CHaro), 119.9 (q, $^{1}J_{C,F}$ = 274 Hz, CF_3), 66.9 (CH_2Cbz), 52.8 (OCH_3), 52.7(CHα), 40.3 (CH_2N), 39.7 (CH), 32.8 (CH_2Ph), 22.8 (CH_2).

HRMS (ESI+TOF) m/z $C_{24}H_{24}F_{3}N_{3}O_{5}$ $[M+H]^+$ calc.492.1740, found 492.1753.

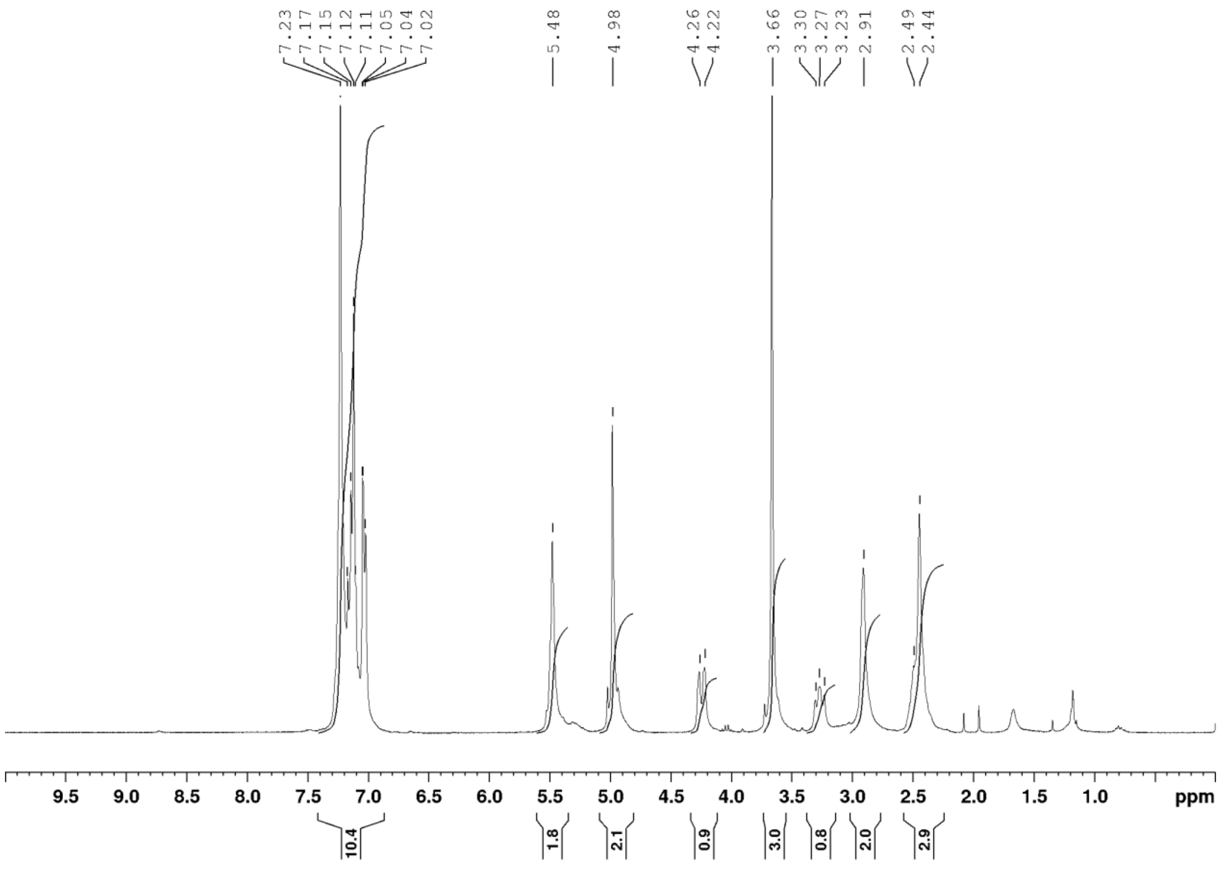

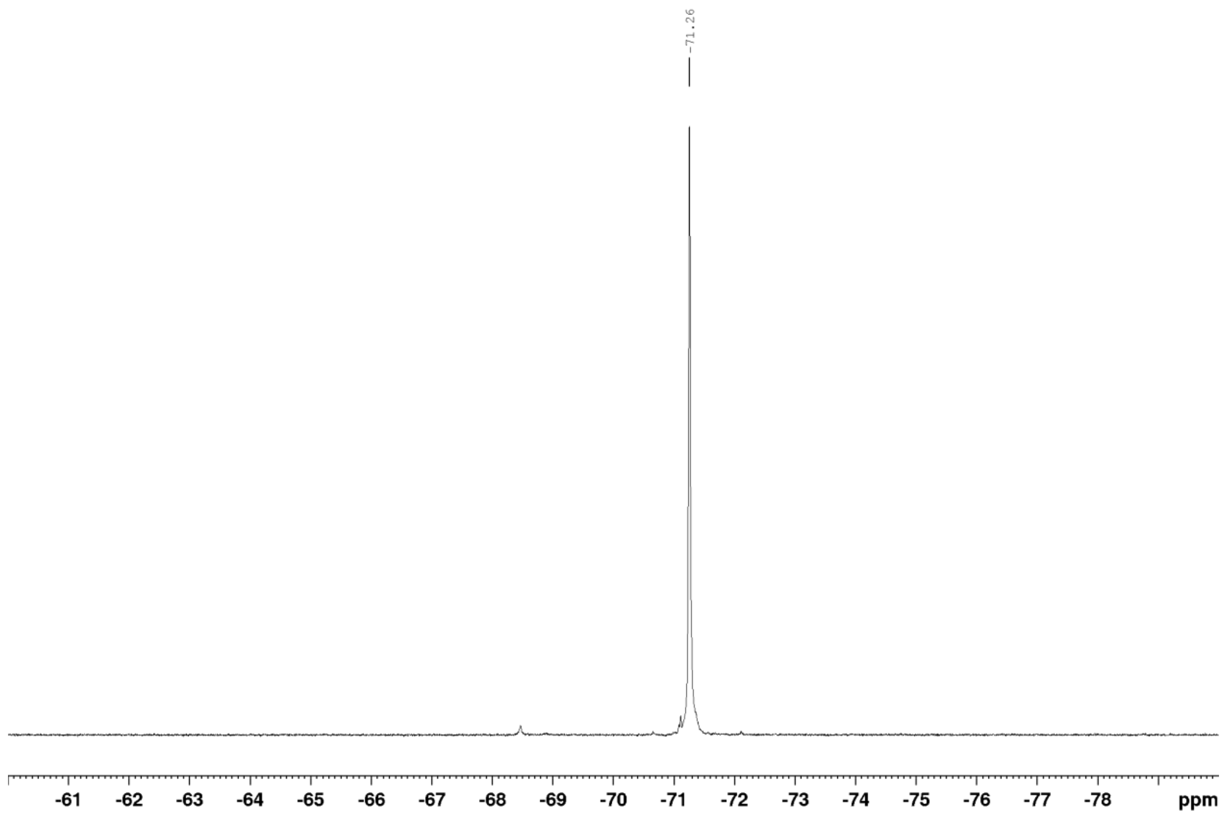
-71.26
-61 -62 -63 -64 -65 -66 -67 -68 -69 -70 -71 -72 -73 -74 -75 -76 -77 -78 ppm

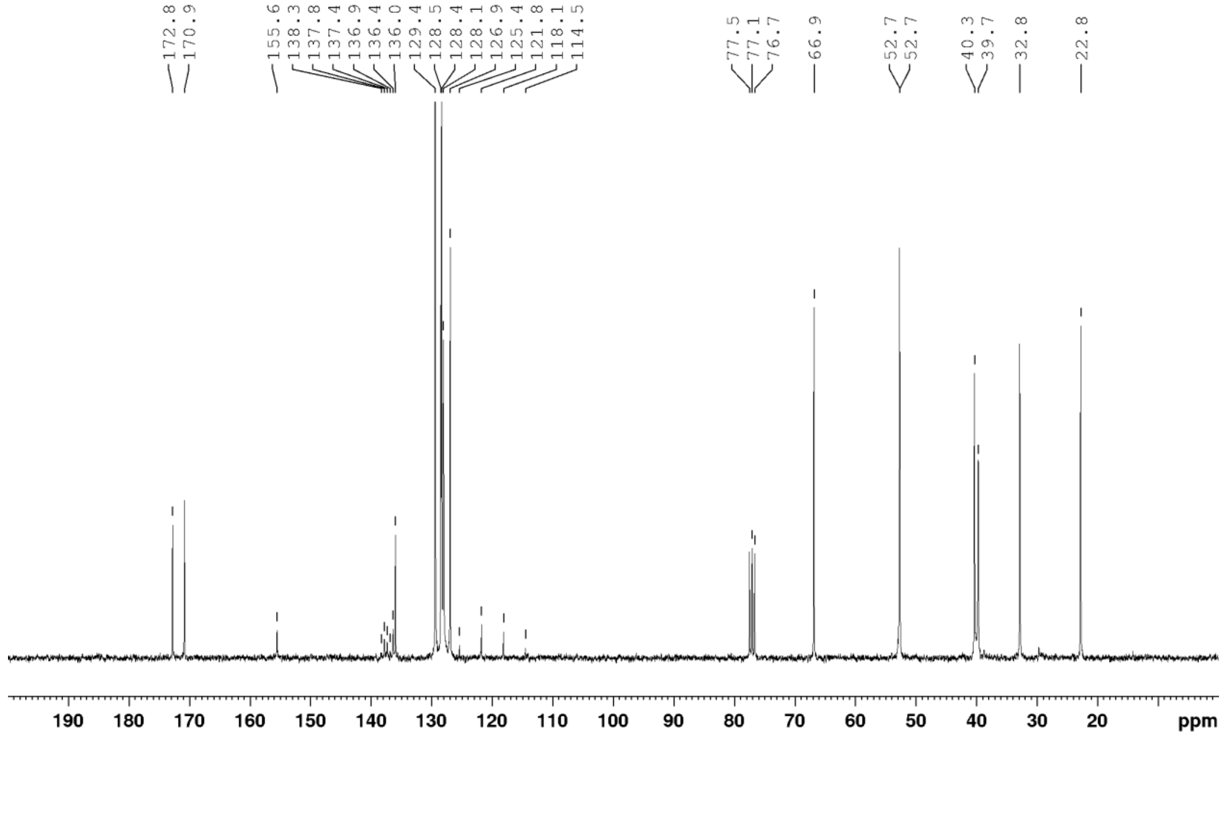
172.8
170.9
155.6
138.3
137.8
137.4
136.9
136.4
136.0
129.4
128.5
128.4
128.1
126.9
125.4
121.8
118.1
114.5
77.5
77.1
76.7
66.9
52.7
52.7
40.3
39.7
32.8
22.8
190 180 170 160 150 140 130 120 110 100 90 80 70 60 50 40 30 20 ppm

Methyl (*R*)-2-(((benzyloxy)carbonyl)-L-phenylalanyl)-6-(trifluoromethyl)-2,3,4,5-tetrahydropyridazine-4-carboxylate (7e')

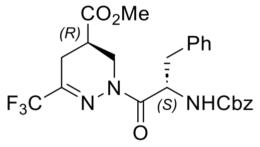

^{1}H NMR (200 MHz, $CDCl_3$): δ= 7.23-7.02 (m, 10H, Haro), 5.40-5.39 (m, 2H, NHCbz and CHα), 4.95 (s, 2H, CH_2Cbz), 4.12 (dd, $^2J_{H,H}$ = 13.6 Hz, $^3J_{H,H}$ = 3.1 Hz 1H, CH_2N), 3.66 (s, 3H, OCH_3), 3.51 (dd, $^2J_{H,H}$ = 13.4 Hz, $^3J_{H,H}$ = 8.6 Hz , 1H, CH_2N), 3.01 (dd, $^2J_{H,H}$ = 13.6 Hz, $^3J_{H,H}$ = 4.0 Hz, 1H, CH_2Ph), 2.87 (m, 1H, CH), 2.76 (dd, $^2J_{H,H}$ = 13.7 Hz, $^3J_{H,H}$ = 7.1 Hz , 1H, CH_2Ph), 2.53 (m, 2H, CH_2).

^{19}F NMR (188 MHz, $CDCl_3$): δ = - 71.24 (s, 3F).

^{13}C NMR (75 MHz, $CDCl_3$): δ = 172.8 (CON), 171.1 (COCbz), 155.7 (CO), 137.5 (q, $^2J_{C,F}$ = 35.8 Hz, C=N), 129.4 (Caro), 129.3 (Caro), 128.5, 128.4, 128.1, 128.0, 127.0 (CHaro), 120.1 (q, $^1J_{C,F}$ = 274 Hz, CF_3), 66.8 (CH_2Cbz), 53.0 (OCH_3), 52.7 (CHα), 40.4 (CH_2N), 38.7 (CH), 32.7 (CH_2Ph), 22.7 (CH_2).

HRMS (ESI+TOF) m/z $C_{24}H_{24}F_3N_3O_5$ $[M+H]^+$ calc.492.1740, found 492.1732.

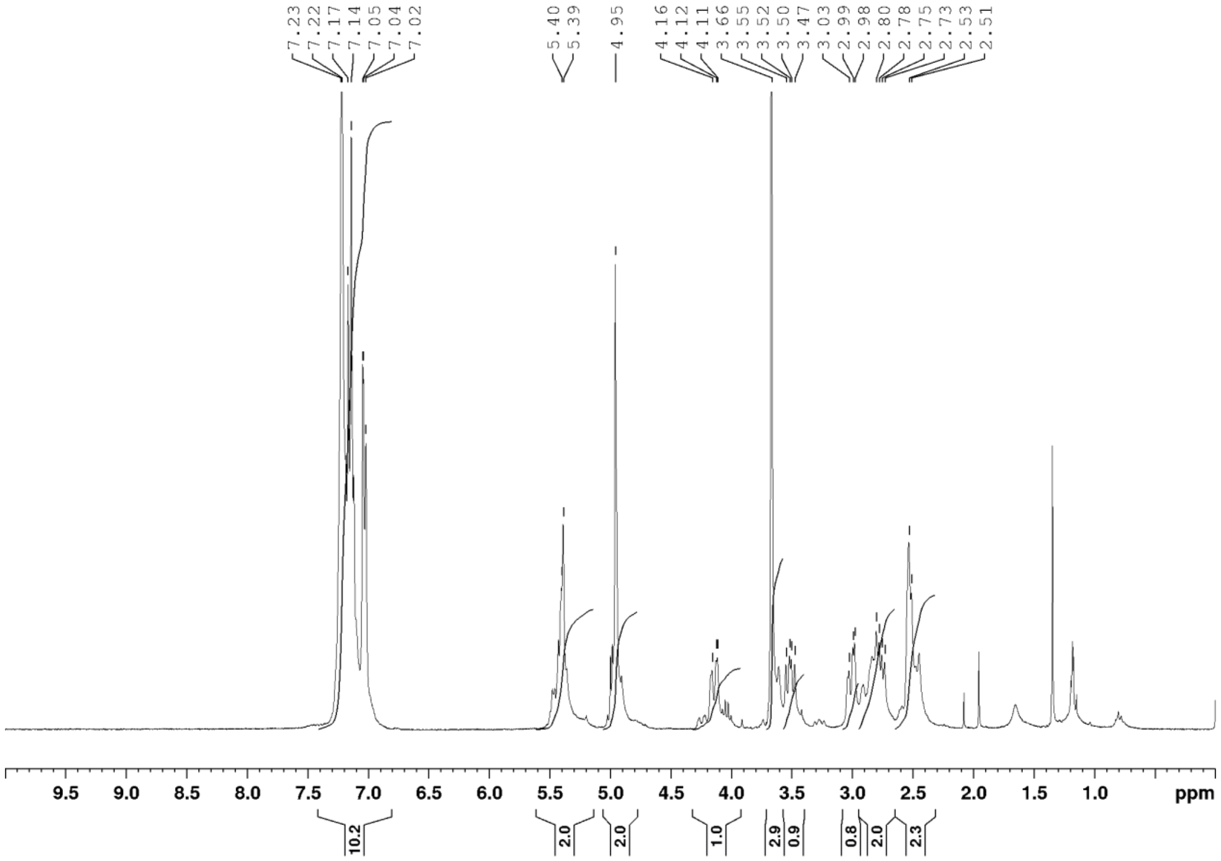

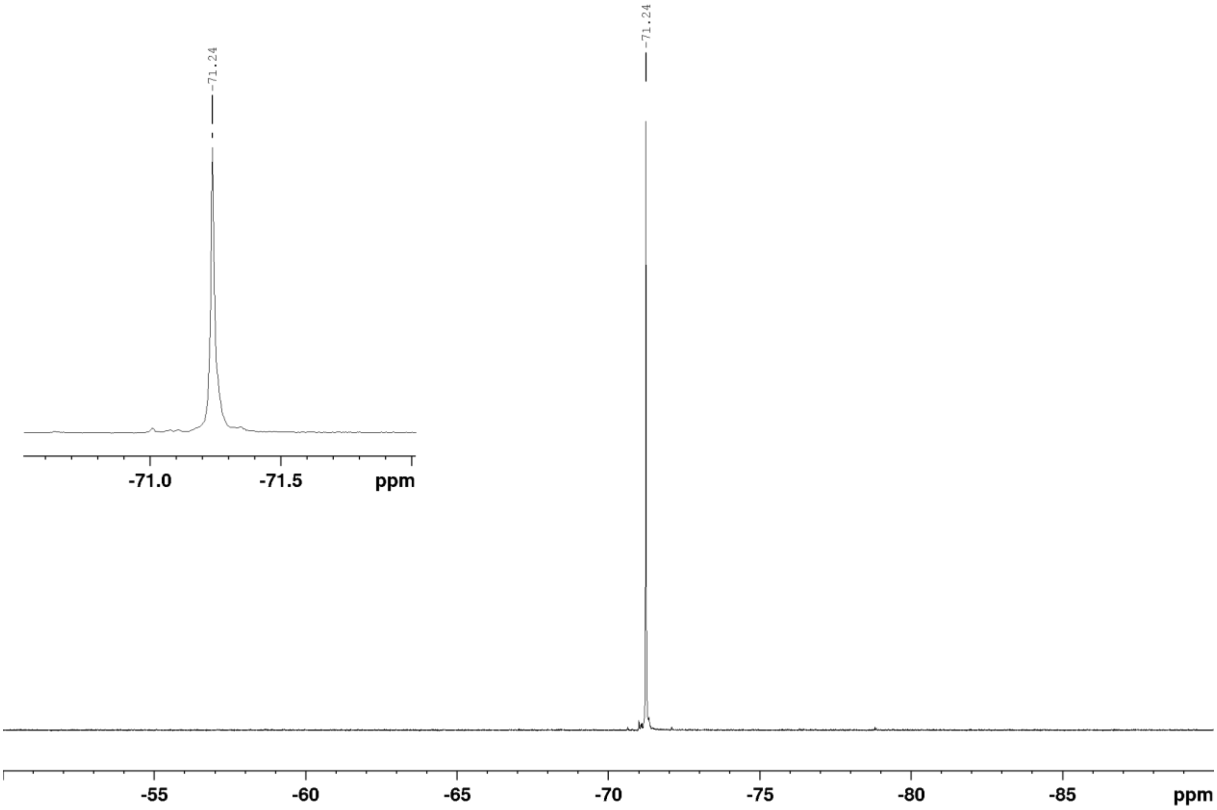
-71.24
-71.24
-71.0
-71.5
ppm
-55
-60
-65
-70
-75
-80
-85
ppm

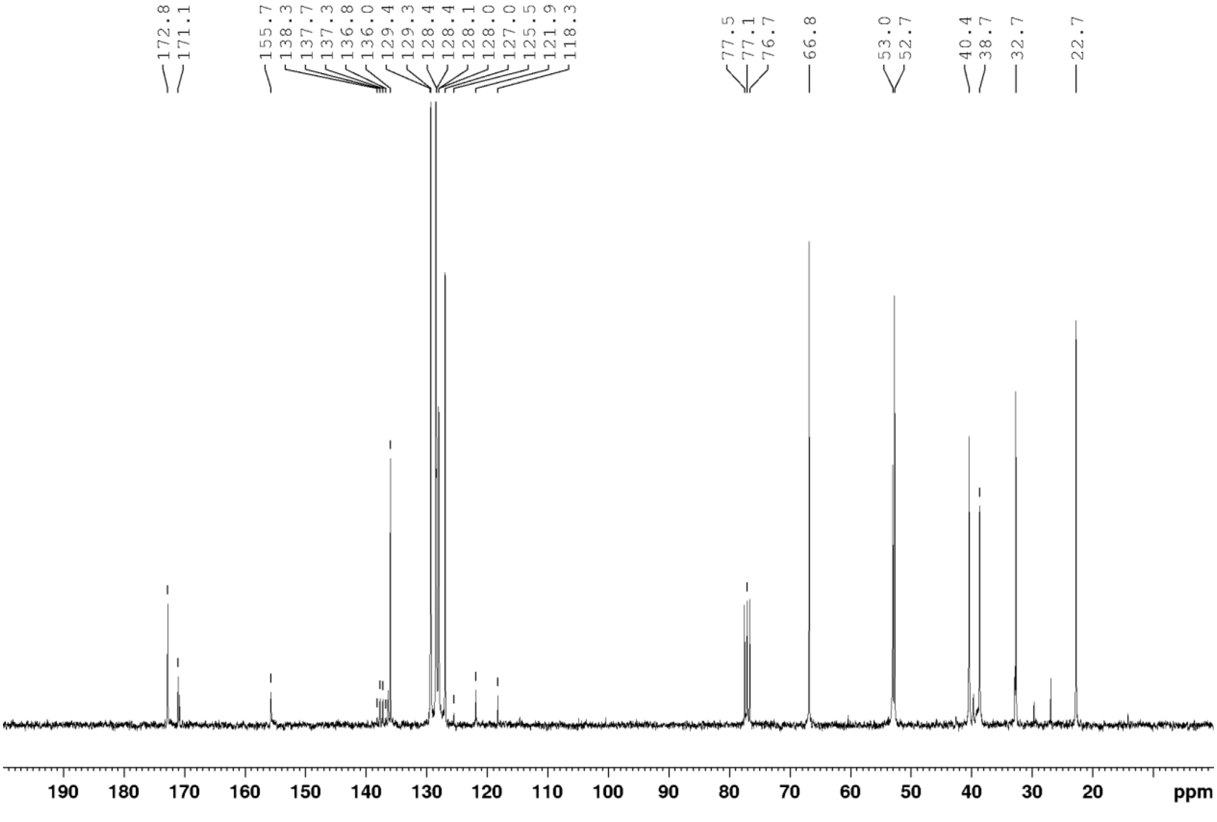
172.8
171.1
155.7
138.3
137.7
137.3
136.8
136.0
129.4
129.3
128.4
128.4
128.1
128.0
127.0
125.5
121.9
118.3
77.5
77.1
76.7
66.8
53.0
52.7
40.4
38.7
32.7
22.7
190
180
170
160
150
140
130
120
110
100
90
80
70
60
50
40
30
20
ppm

Methyl 2-(((benzyloxy)carbonyl)-L-phenylalanyl)-6-(difluoromethyl)-2,3,4,5-tetrahydropyridazine-4-carboxylate (7f)

The product **7f** is obtained following the general procedure **D** as a yellow oil (0.646 g, 65%).

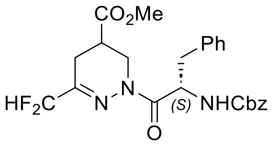

Chemical Formula: $C_{24}H_{25}F_2N_3O_5$
Exact Mass: 473,1762

Methyl (*S*)-2-(((benzyloxy)carbonyl)-L-phenylalanyl)-6-(difluoromethyl)-2,3,4,5-tetrahydropyridazine-4-carboxylate (7f)

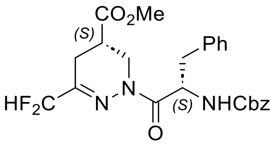

^{1}H NMR (300 MHz, $CDCl_3$): δ= 7.24-7.02 (m, 10H, Haro), 5.83 (t, $^2J_{H,F}$ = 54.8 Hz, 1H, CF_2H), 5.57 (d, $^3J_{H,H}$ = 8.5 Hz, 1H, NHCbz), 5.49 (q, $^3J_{H,H}$ = 7.3 Hz, 1H, CHα), 5.00 (s, 2H, CH_2Cbz), 4.29 (d, $^2J_{H,H}$ =14.3 Hz, 1H, CHN), 3.65 (s, 3H, OCH_3), 3.16 (d, $^2J_{H,H}$ = 11.1 Hz, 1H, CHN), 2.98 (dd, $^2J_{H,H}$ = 13.3 Hz, $^3J_{H,H}$ = 5.9 Hz, 1H, CH_2Ph), 2.85 (dd, $^2J_{H,H}$ = 12.9 Hz, $^3J_{H,H}$ = 7.4 Hz, 1H, CH_2Ph), 2.49-2.31 (m, 3H, CH and CH_2).

^{19}F NMR (188 MHz, $CDCl_3$): δ = - 118.0 (dd, $^2J_{F,F}$ = 319 Hz, $^2J_{H,F}$ = 54.6 Hz 1F), -120.7 (dd, $^2J_{F,F}$ = 319 Hz, $^2J_{H,F}$ = 55.2 Hz 1F).

^{13}C NMR (75 MHz, $CDCl_3$): δ = 171.4 (CON), 170.2 (COCbz), 154.6 (CO), 142.6 (t, $^2J_{C,F}$ = 31.1 Hz, C=N), 135.1, 128.4 (Caro), 127.5, 127.3, 127.1, 127.0, 125.6 (CHaro), 112.6 (t, $^1J_{C,F}$ = 238 Hz, CF_2H), 65.8 (CH_2Cbz), 51.6 (CHα), 51.2 (OCH_3), 39.5 (CH_2N), 39.0 (CH), 31.9 (CH_2Ph), 20.2 (CH_2).

HRMS (ESI+TOF) m/z $C_{24}H_{24}F_3N_3O_5$ $[M+H]^+$ calc. 474.1834, found 474.1854.

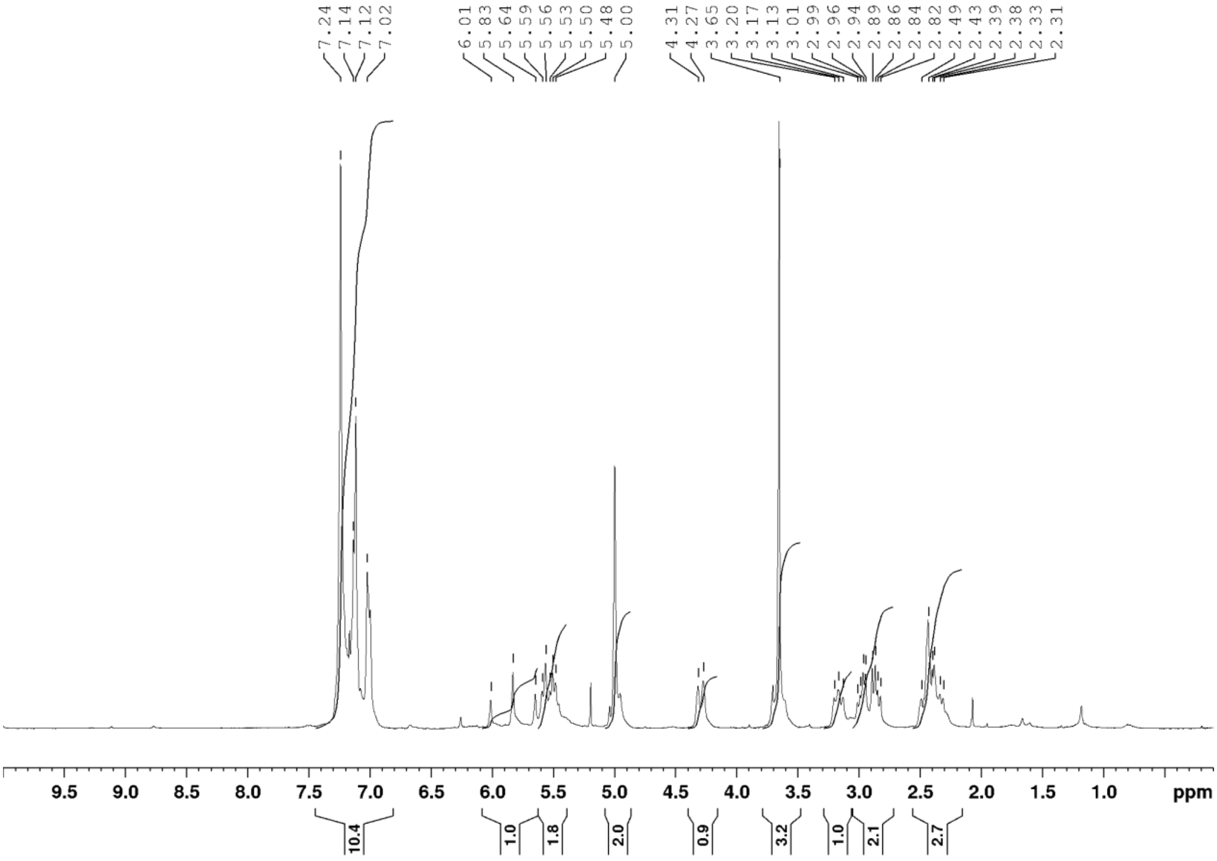

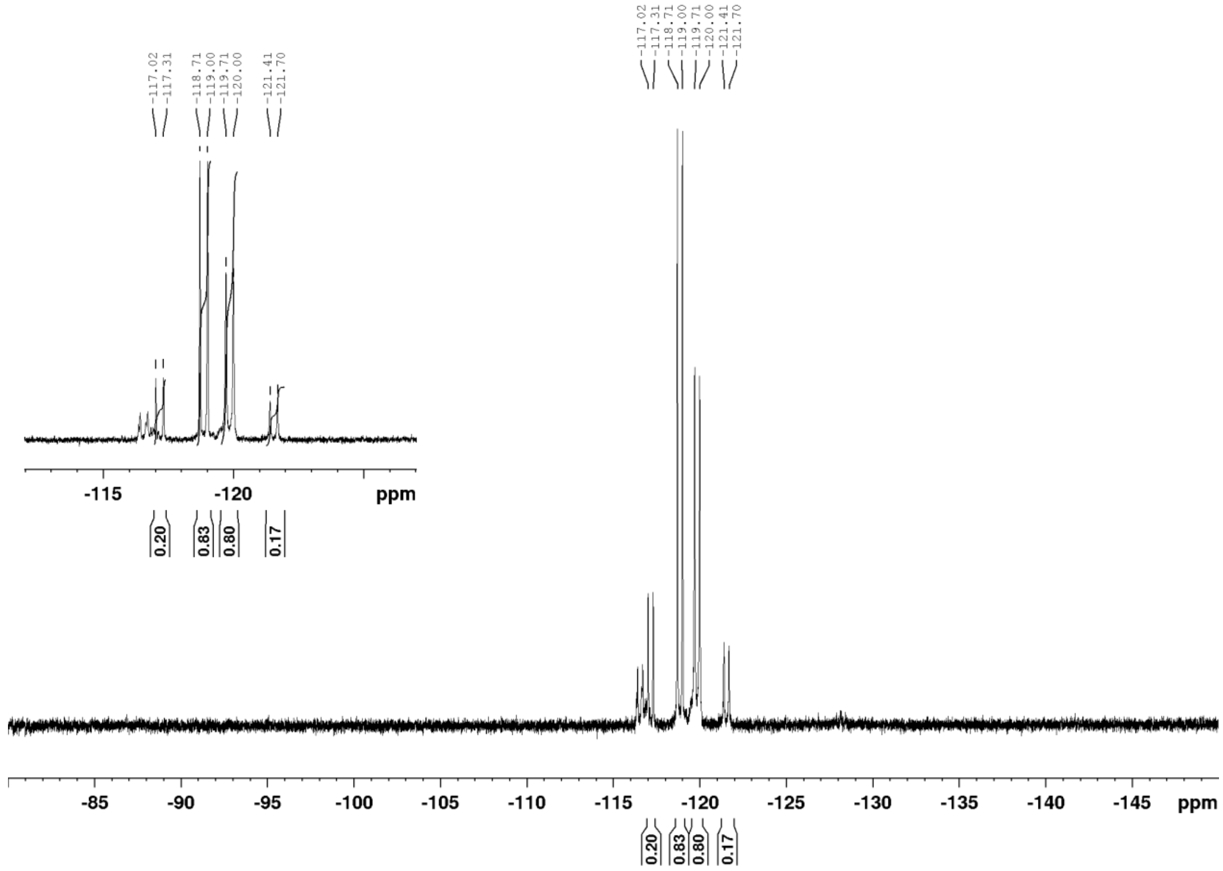
-117.02
-117.31
-118.71
-119.00
-119.71
-120.00
-121.41
-121.70
-115
-120
ppm
0.20
0.83
0.80
0.17
-85
-90
-95
-100
-105
-110
-115
-120
-125
-130
-135
-140
-145
ppm

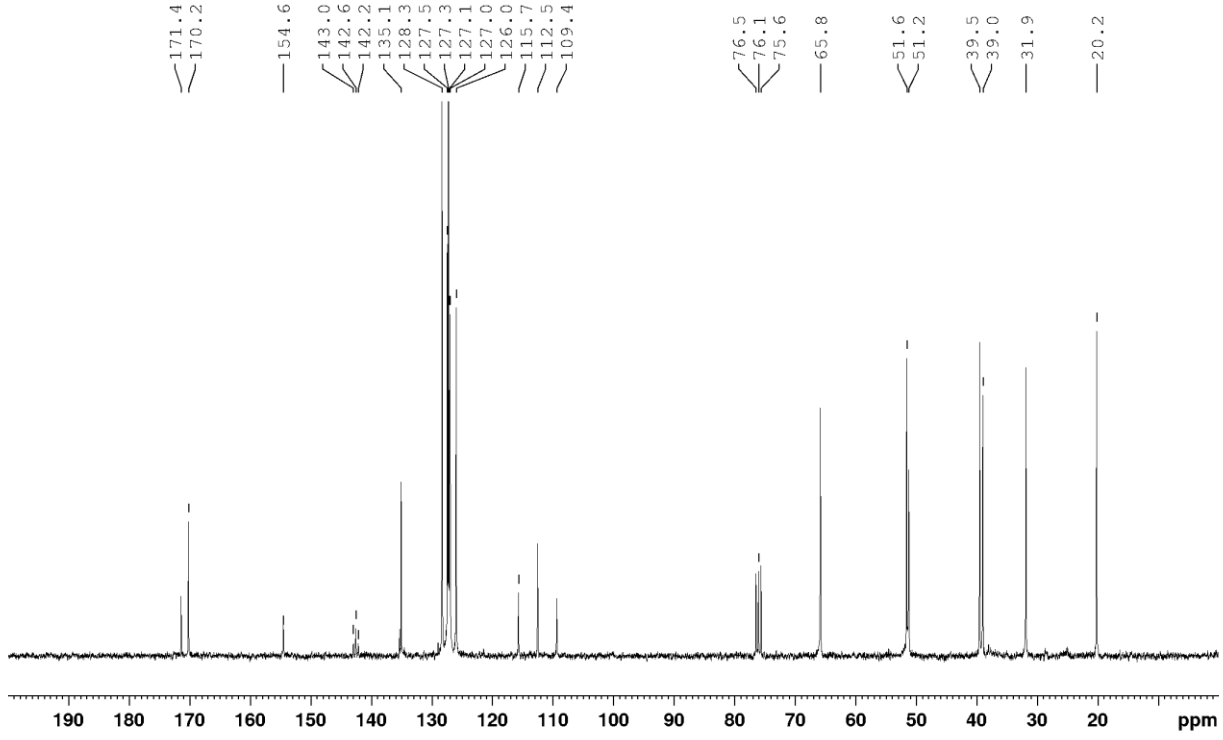
171.4
170.2
154.6
143.0
142.6
142.2
135.1
128.3
127.5
127.3
127.1
127.0
126.0
115.7
112.5
109.4
76.5
76.1
75.6
65.8
51.6
51.2
39.5
39.0
31.9
20.2
190
180
170
160
150
140
130
120
110
100
90
80
70
60
50
40
30
20
ppm

Methyl (*R*)-2-(((benzyloxy)carbonyl)-L-phenylalanyl)-6-(difluoromethyl)-2,3,4,5-tetrahydropyridazine-4-carboxylate (7f')

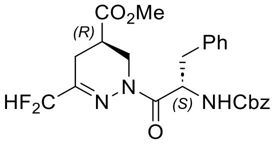

^{1}H NMR (300 MHz, $CDCl_3$): δ= 7.24-6.99 (m, 10H, Haro), 5.88 (t, $^2J_{H,F}$ = 54.8 Hz, 1H, CF_2H), 5.45 (brs, 2H, NHCbz and CHα), 4.98 (s, 2H, CH_2Cbz), 4.13 (dd, $^2J_{H,H}$ =13.4 Hz, $^3J_{H,H}$ = 3.3 Hz, 1H, CH_2N), 3.66 (s, 3H, OCH_3), 3.47 (dd, $^2J_{H,H}$ = 13.4 Hz, $^3J_{H,H}$ = 8.8 Hz, 1H, CH_2N), 2.95 (m, 1H, CH_2Ph), 2.86 (d, $^3J_{H,H}$ = 5.9 Hz, 1H, CH_2Ph), 2.79 (m, 1H, CH), 2.55 (dd, $^2J_{H,H}$ = 18.6Hz, $^3J_{H,H}$ = 5.3 Hz, 1H, CH_2), 2.43 (dd, $^2J_{H,H}$ = 18.4 Hz, $^3J_{H,H}$ = 8.1 Hz, 1H, CH_2).

^{19}F NMR (188 MHz, $CDCl_3$): δ = - 118.2 (dd, $^2J_{F,F}$ = 319 Hz, $^2J_{H,F}$ = 54.1 Hz 1F), -120.2 (dd, $^2J_{F,F}$ = 319 Hz, $^2J_{H,F}$ = 54.3 Hz 1F).

^{13}C NMR (75 MHz, $CDCl_3$): δ = 172.5 (CON), 171.4 (COCbz), 155.7 (CO), 143.4 (t, $^2J_{C,F}$ = 31.2 Hz, C=N), 136.1, 129.3 (Caro), 128.5, 128.4, 128.1, 128.0, 127.0 (CHaro), 113.7 (t, $^1J_{C,F}$ = 238 Hz, CF_2H), 66.9 (CH_2Cbz), 52.6 (CHα), 52.5 (OCH_3), 40.7 (CH_2N), 39.1 (CH), 32.7 (CH_2Ph), 21.1 (CH_2).

HRMS (ESI+TOF) m/z $C_{24}H_{25}F_5N_3O_5$ $[M+H]^+$ calc. 474.1834, found 474.1838.

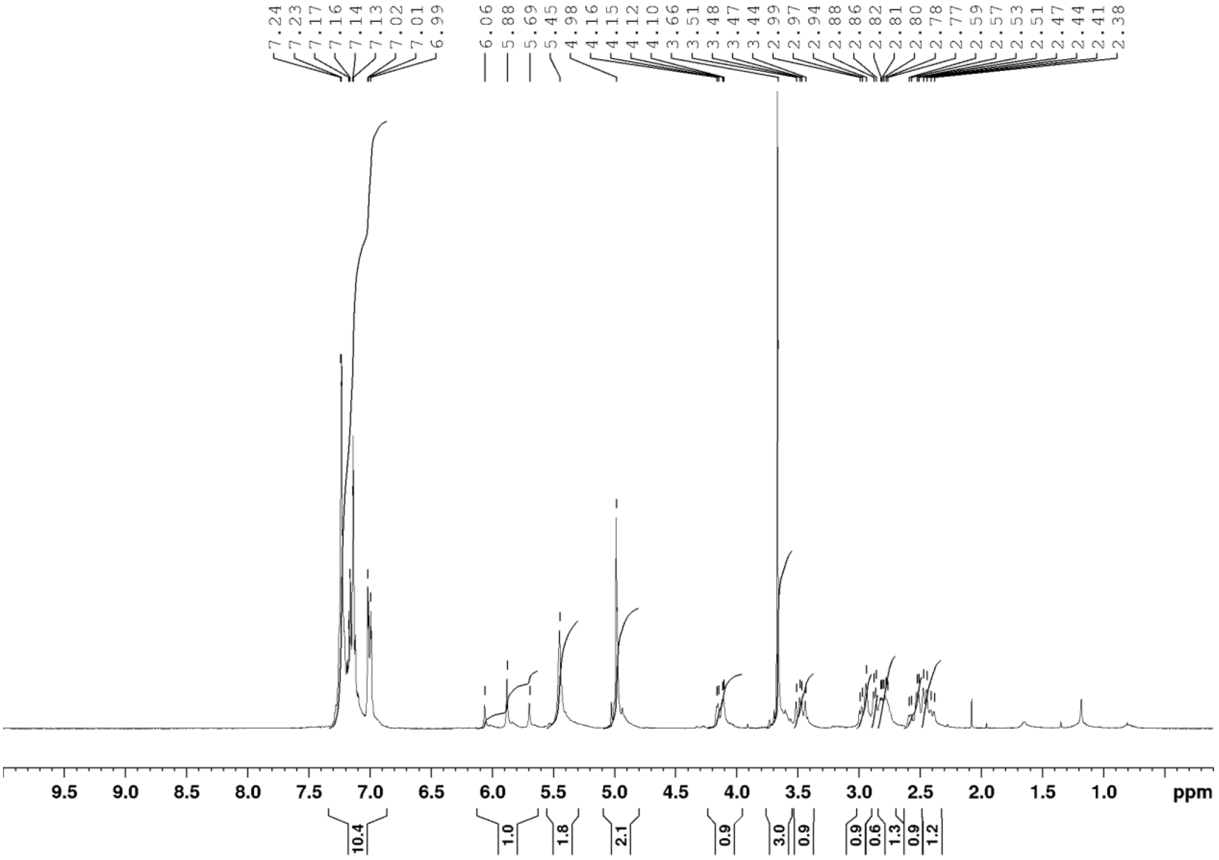

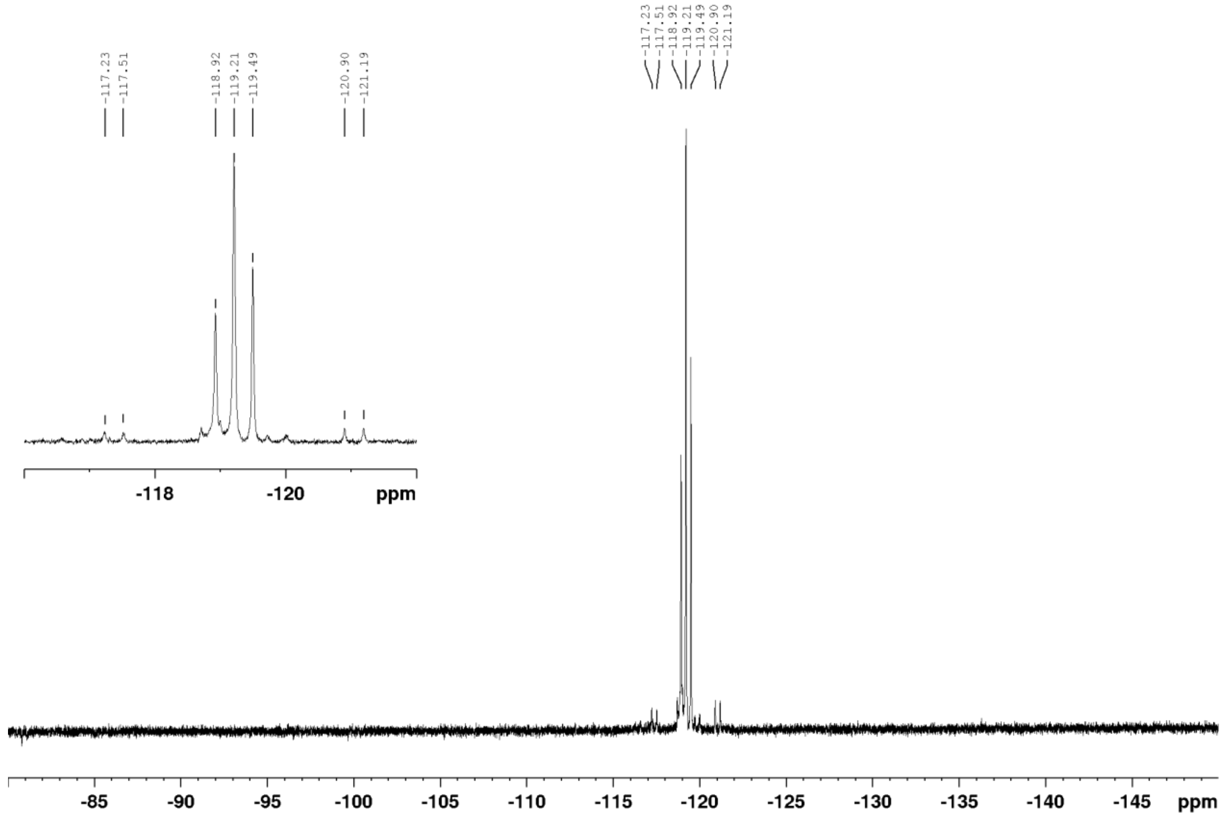

-117.23
-117.51
-118.92
-119.21
-119.49
-120.90
-121.19
-118
-120
ppm
-85
-90
-95
-100
-105
-110
-115
-120
-125
-130
-135
-140
-145
ppm

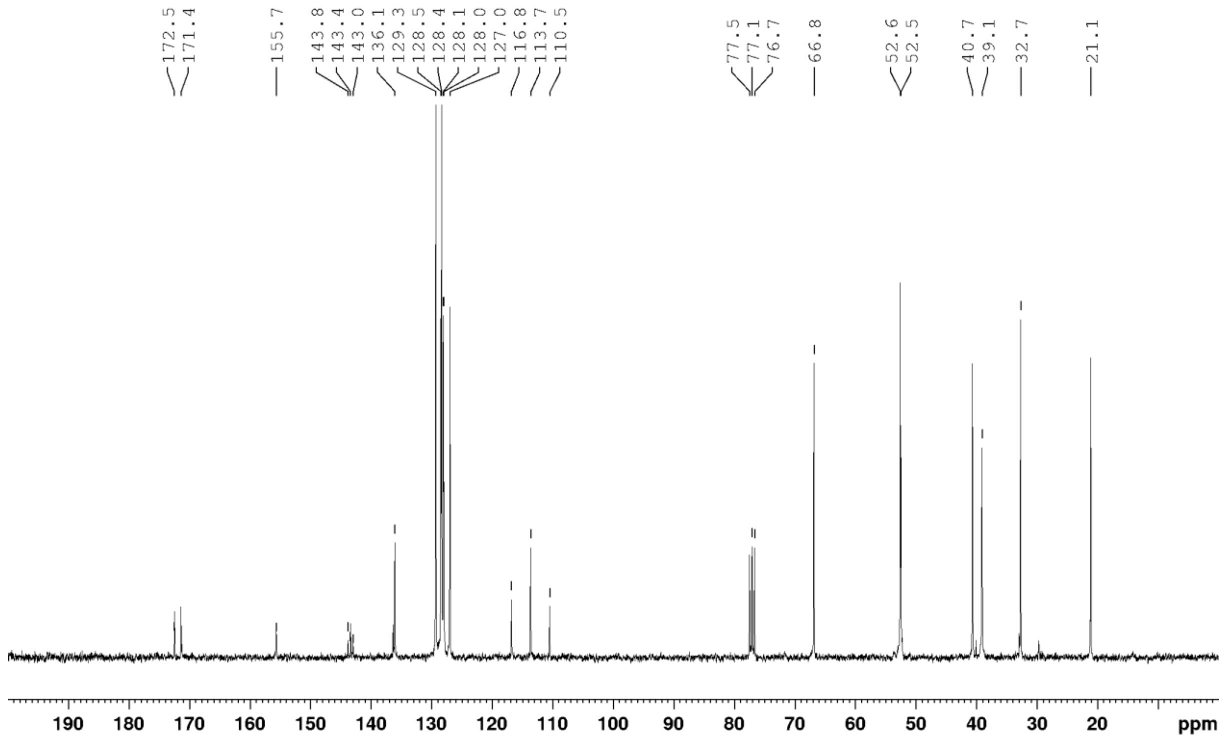

172.5
171.4
155.7
143.8
143.4
143.0
136.1
129.3
128.5
128.4
128.1
128.0
127.0
116.8
113.7
110.5
77.5
77.1
76.7
66.8
52.6
52.5
40.7
39.1
32.7
21.1
190 180 170 160 150 140 130 120 110 100 90 80 70 60 50 40 30 20
ppm

e) **Tripeptides 8e, 8e', 8f and 8f':**

Methyl ((S)-2-(((benzyloxy)carbonyl)-L-phenylalanyl)-6-(trifluoromethyl)-2,3,4,5-tetrahydropyridazine-4-carbonyl)-L-valinate (8e)

The product **8e** is obtained following the general procedure **E** as a white foam (160 mg, 65%).

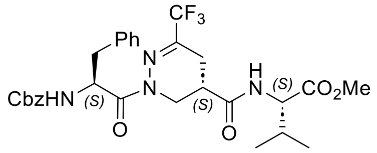

Chemical Formula: $C_{29}H_{33}F_3N_4O_6$
Exact Mass: 590,2352

^{1}H NMR (300 MHz, $CDCl_3$): δ= 7.30-7.12 (m,10H, Haro), 6.46 (m, 1H, NHVal), 5.60-5.53 (m, 2H, NHPhe and CHαPhe), 5.0 (m, 2H, CH_2Cbz), 4.56 (dd, $^3J_{H,H}$ = 8.4 Hz, $^3J_{H,H}$ = 4.9 Hz, 1H, CHαVal), 4.43 (d, $^2J_{H,H}$ = 12.7 Hz, 1H, CH2N), 3.74 (s, 3H, OCH_3), 3.19 (t, $^2J_{H,H}$ = 11.9 H, 1H, CH_2N), 3.06 - 2.85 (m, 2H, CH_2Ph), 2.60 (m, 1H, CH_2), 2.48 (m, 1H, CH_2), 2.38 (m, 1H, CH), 2.16 (s, $^3J_{H,H}$ = 6.8 Hz, 1H, CHiPr), 0.93 (d, $^3J_{H,H}$ = 6.8 Hz, 3H, CH_3), 0.91 (d, $^3J_{H,H}$ = 6.8 Hz, 3H, CH_3).

^{19}F NMR (188 MHz, $CDCl_3$): δ = - 71.1 (s, 3F).

^{13}C NMR (75 MHz, $CDCl_3$): δ = 172.9 (CON), 172.3 (CONH), 170.1 (COCbz), 155.7 (CO), 138.4 (q, $^2J_{C,F}$ = 35 Hz, C=N), 136.3, 129.5 (Caro), 128.5, 128.4, 128.1, 126.9 (CHaro), 120.1 (q, $^1J_{C,F}$ = 274 Hz, CF_3), 67.0 (CH_2Cbz), 57.5 (CHαVal), 53.0 (CHαPhe), 52.5 (OCH_3), 41.0 (CH_2N), 39.7 (CH_2Ph), 34.0 (CH), 31.3 (CHiPr), 23.7 (CH_2), 19.0, 18.0 (CH_3iPr).

HRMS (ESI+TOF) m/z $C_{29}H_{33}F_3N_4O_6$ $[M+H]^+$ calc.591.2425, found 591.2430.

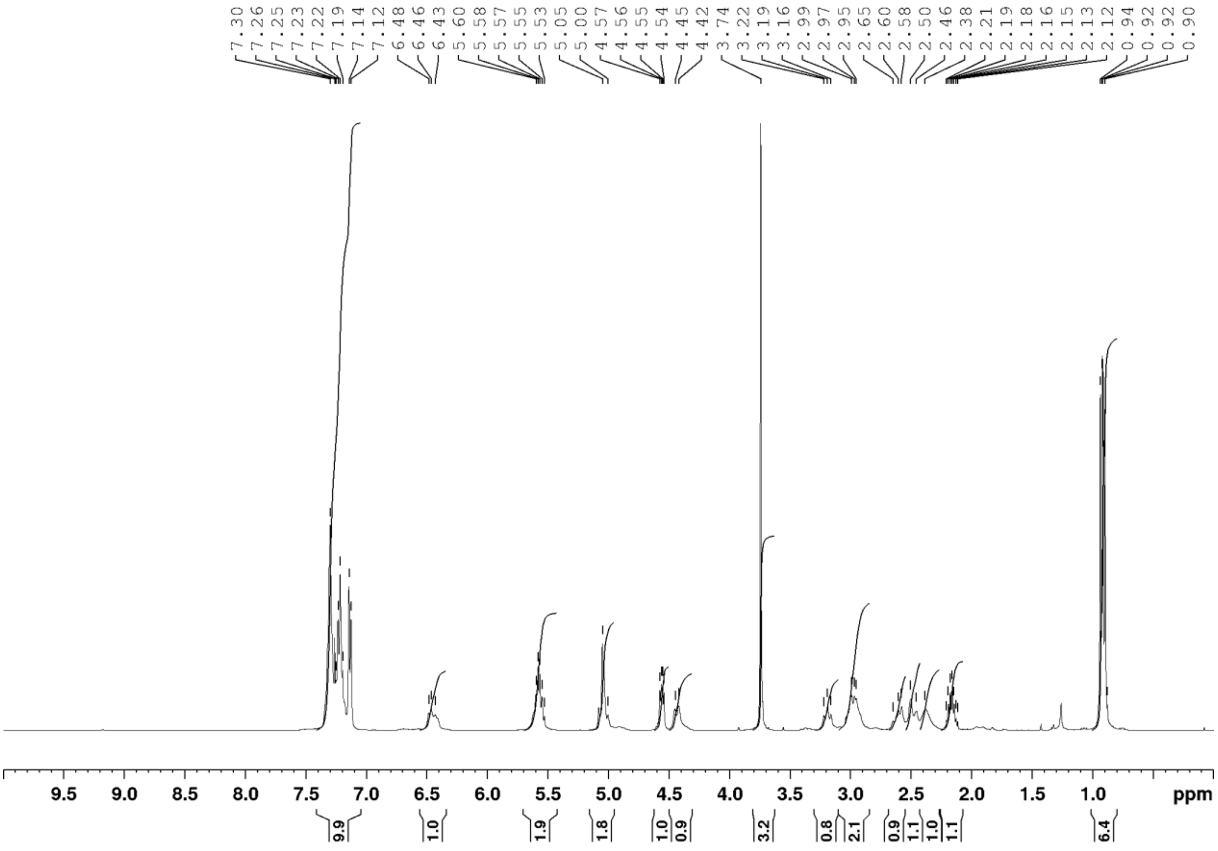

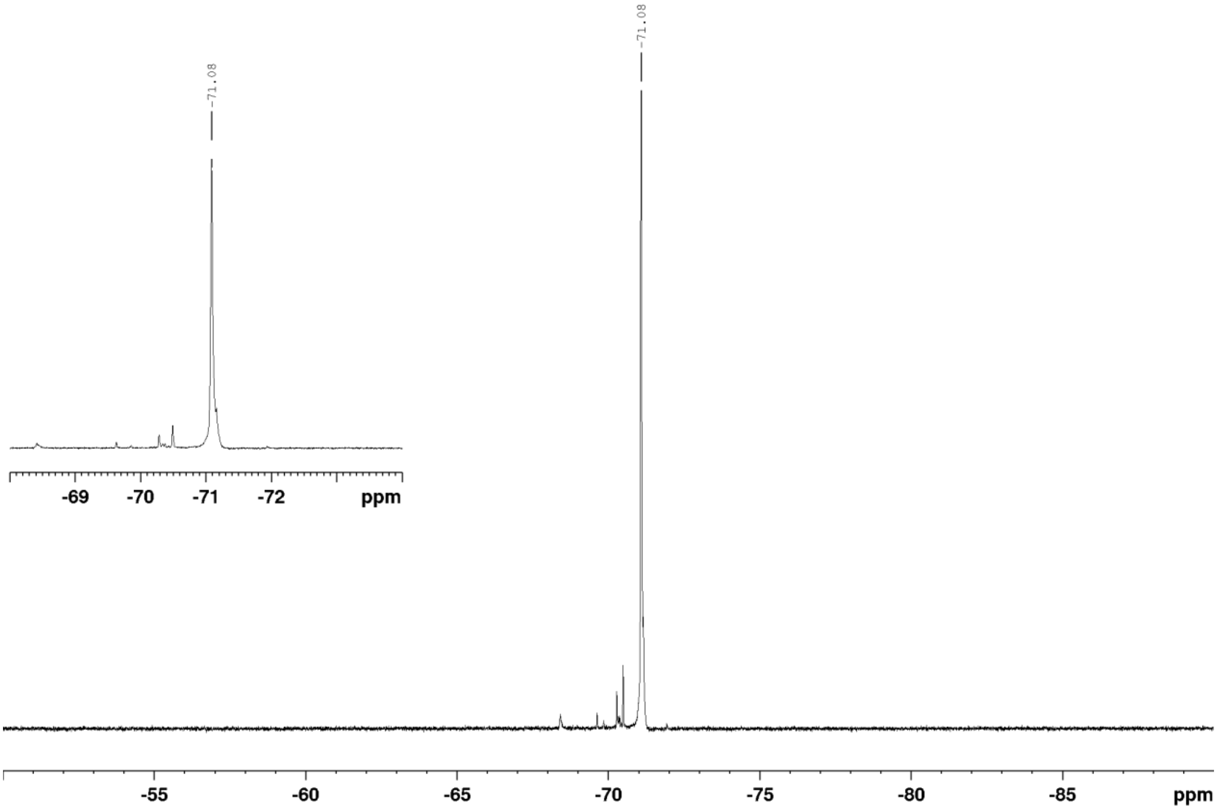
-71.08
-69 -70 -71 -72 ppm
-71.08
-55 -60 -65 -70 -75 -80 -85 ppm

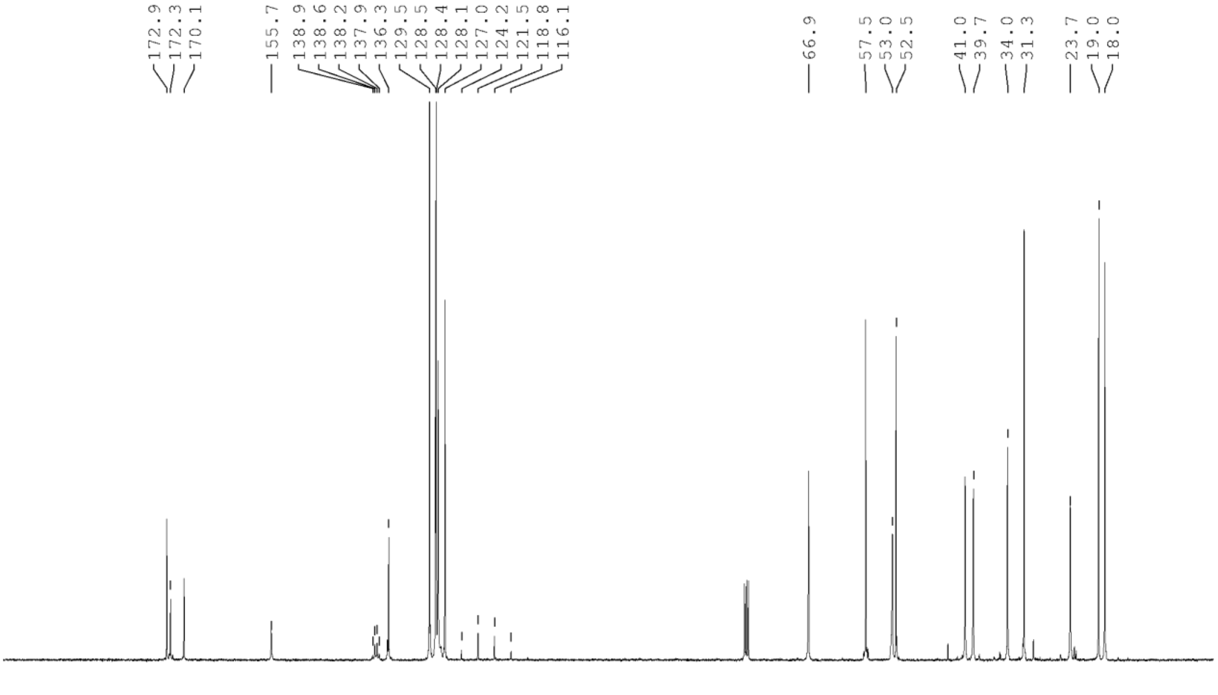
172.9 172.3 170.1 155.7 138.9 138.6 138.2 137.9 136.3 129.5 128.5 128.4 128.1 127.0 124.2 121.5 118.8 116.1 66.9 57.5 53.0 52.5 41.0 39.7 34.0 31.3 23.7 19.0 18.0
190 180 170 160 150 140 130 120 110 100 90 80 70 60 50 40 30 20 ppm

2D ^{1}H-^{1}H COSY experiments of compound 8e:

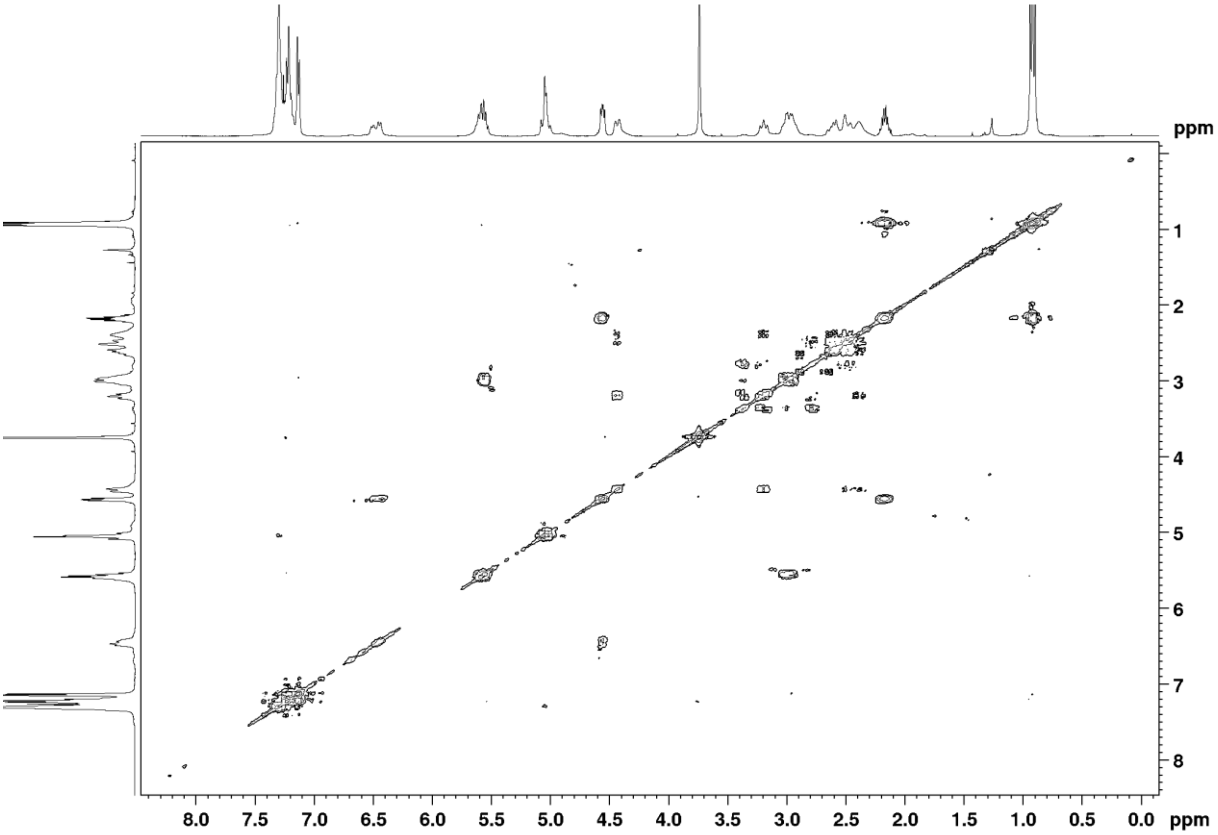

2D ^{1}H-^{1}H NOESY experiments of compound 8e

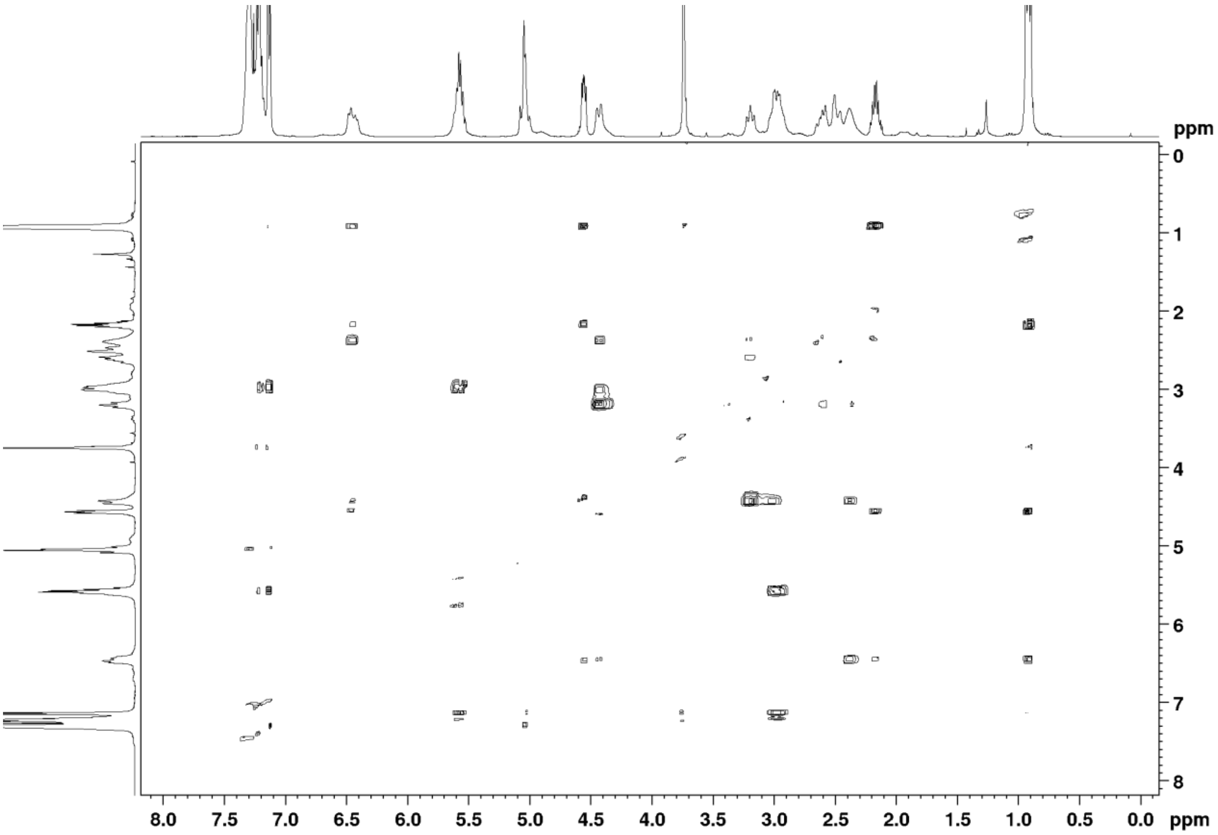

2D ^{19}F-^{1}H NOESY experiments of compound 8e

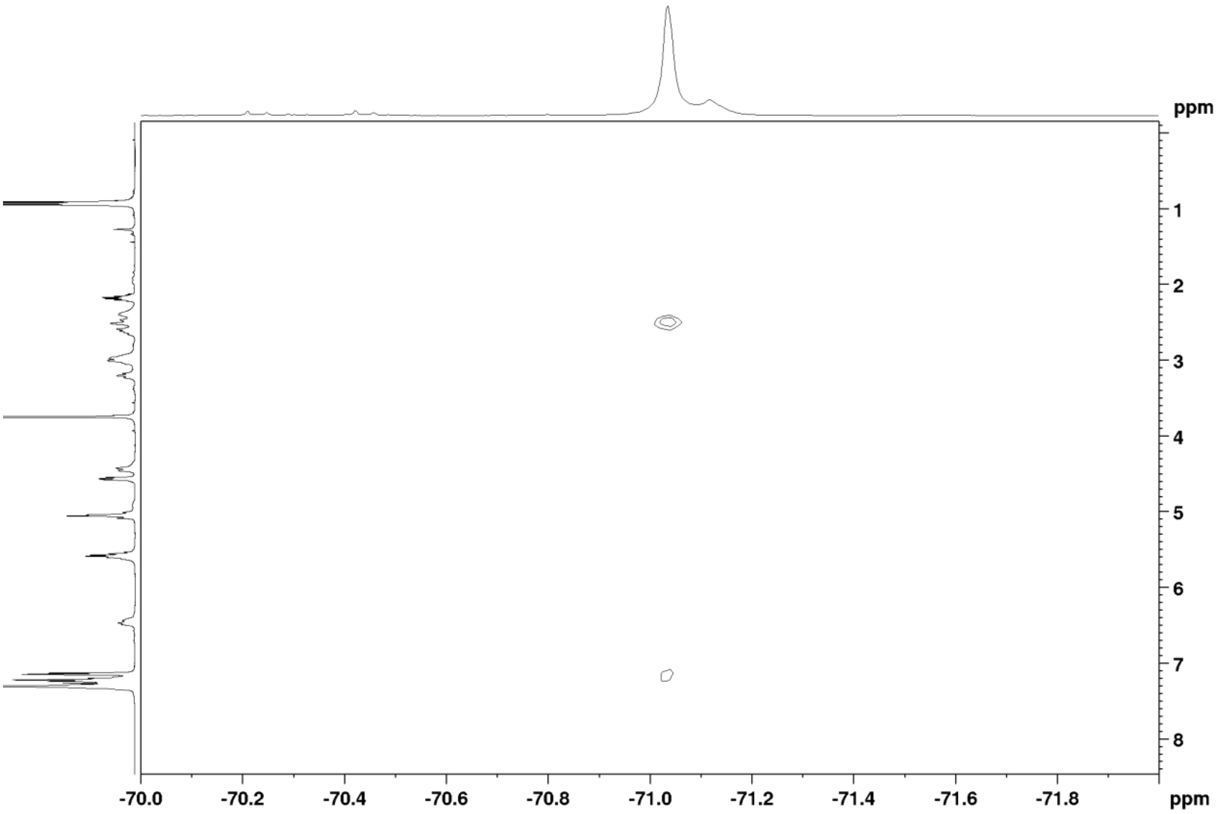

Methyl ((R)-2-(((benzyloxy)carbonyl)-L-phenylalanyl)-6-(trifluoromethyl)-2,3,4,5-tetrahydropyridazine-4-carbonyl)-L-valinate (8e')

The product **8e'** is obtained following the general procedure **E** as a white foam (115 mg, 61%).

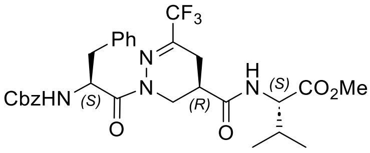

Chemical Formula: $C_{29}H_{33}F_3N_4O_6$
Exact Mass: 590,2352

^{1}H NMR (300 MHz, $CDCl_3$): δ= 7.27-7.06 (m,10H, Haro), 6.71 (d, $^{2}J_{H,H}$ = 8.7 Hz, 1H, NHVal), 5.54 (d, $^{2}J_{H,H}$ = 8.5 Hz, 1H, NHCbz), 5.41 (td, $^{3}J_{H,H}$ = 8.6 Hz, $^{3}J_{H,H}$ = 5.0 Hz , 1H, CHαPhe), 4.96 (d, $^{2}J_{H,H}$ = 11.6 H, 1H, CH_2Cbz), 4.92 (d, $^{2}J_{H,H}$ = 11.9 H, 1H, CH_2Cbz), 4.51 (dd, $^{3}J_{H,H}$ = 8.8 Hz, $^{3}J_{H,H}$ = 5.0 Hz, 1H, CHαVal), 4.38 (d, $^{2}J_{H,H}$ = 13.2 Hz, 1H, CH_2N), 3.67 (s, 3H, OCH_3), 3.20 (m, 1H, CH_2N), 3.03 (dd, $^{2}J_{H,H}$ = 13.3 Hz, $^{3}J_{H,H}$ = 3.8 Hz, 1H, CH_2Ph), 2.73 (dd, $^{2}J_{H,H}$ = 13.3 Hz, $^{3}J_{H,H}$ = 8.5 Hz, 1H, CH_2Ph), 2.66-2.29 (m, 3H, CH and CH_2), 2.08 (st, $^{3}J_{H,H}$ = 6.5 Hz, 1H, CHiPr), 0.85 (d, $^{3}J_{H,H}$ = 6.5 Hz, 3H, CH_3iPr), 0.81 (d, $^{3}J_{H,H}$ = 6.5 Hz, 3H, CH_3iPr).

^{19}F NMR (188 MHz, $CDCl_3$): δ = - 71.2 (s, 3F).

^{13}C NMR (75 MHz, $CDCl_3$): δ = 173.2 (CON), 172.5 (CONH), 170.1 (COCbz), 155.8 (CO), 138.9 (q, $^{2}J_{C,F}$ = 36 Hz, C=N), 136.3, 136.1 (Caro), 129.4, 129.3, 128.5, 128.0, 127.8, 127.0 (CHaro), 120.1 (q, $^{1}J_{C,F}$ = 274 Hz, CF_3), 66.8 (CH_2Cbz), 57.3 (CHαVal), 53.23 (CHαPhe), 52.4 (OCH_3), 41.4 (CH_2N), 38.5 (CH_2Ph), 33.8 (CH), 31.3 (CHiPr), 22.9 (CH_2), 19.0 (CH_3iPr), 17.8 (CH_3iPr).

HRMS (ESI+TOF) m/z $C_{29}H_{33}F_3N_4O_6$ $[M+H]^+$ calc.591.2425, found 591.2429.

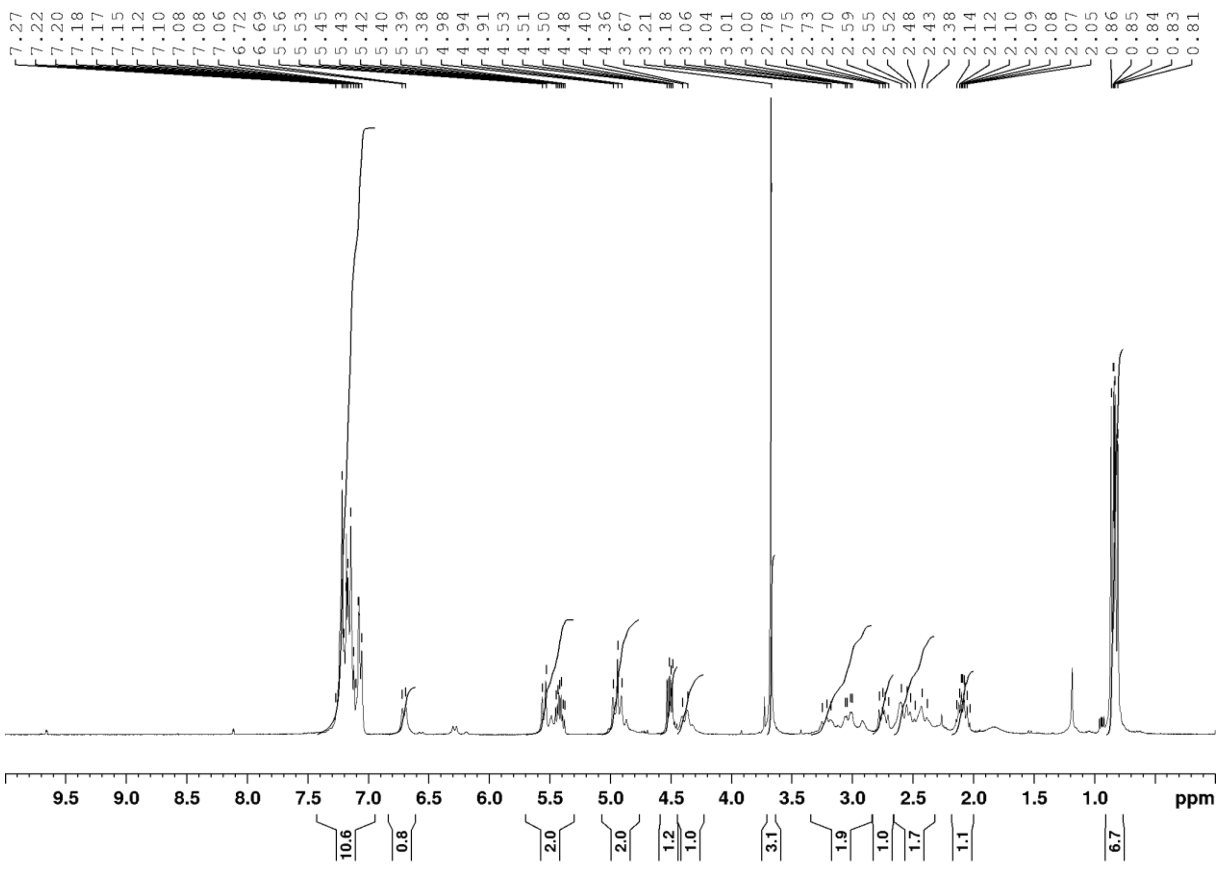

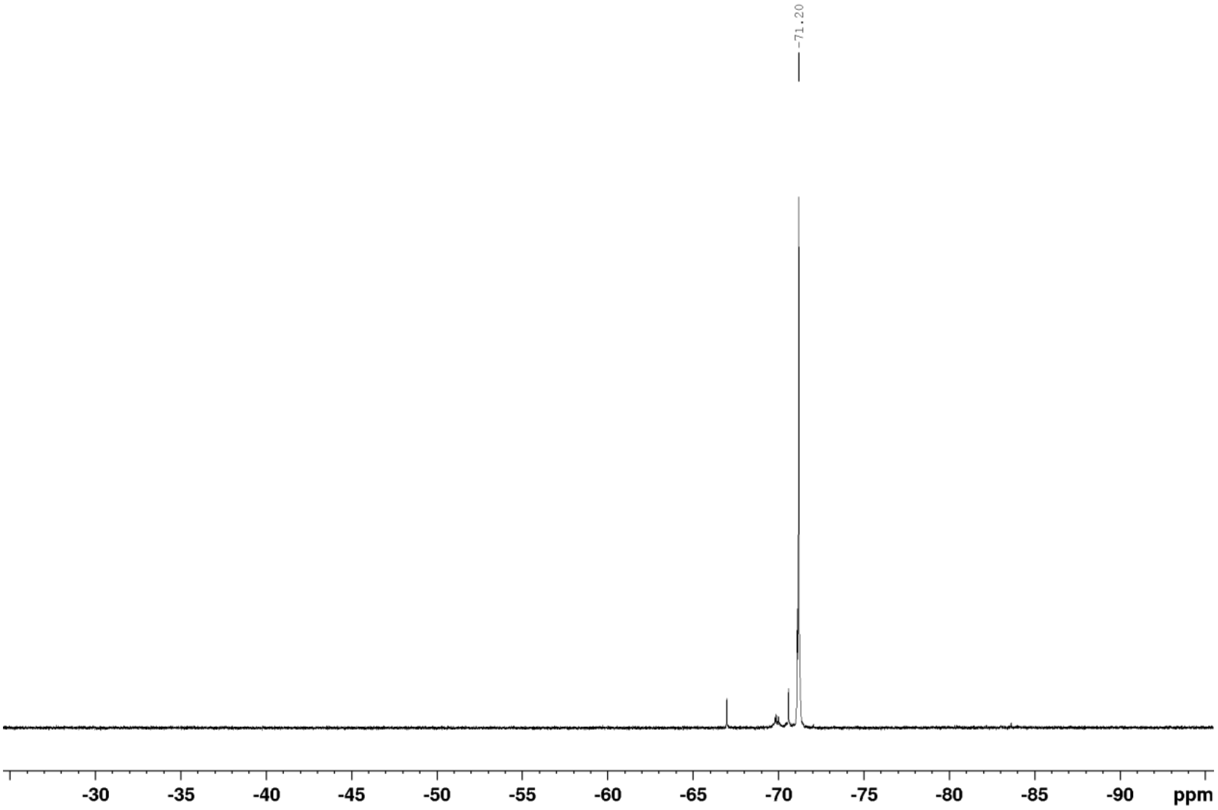
-71.20
-30 -35 -40 -45 -50 -55 -60 -65 -70 -75 -80 -85 -90 ppm

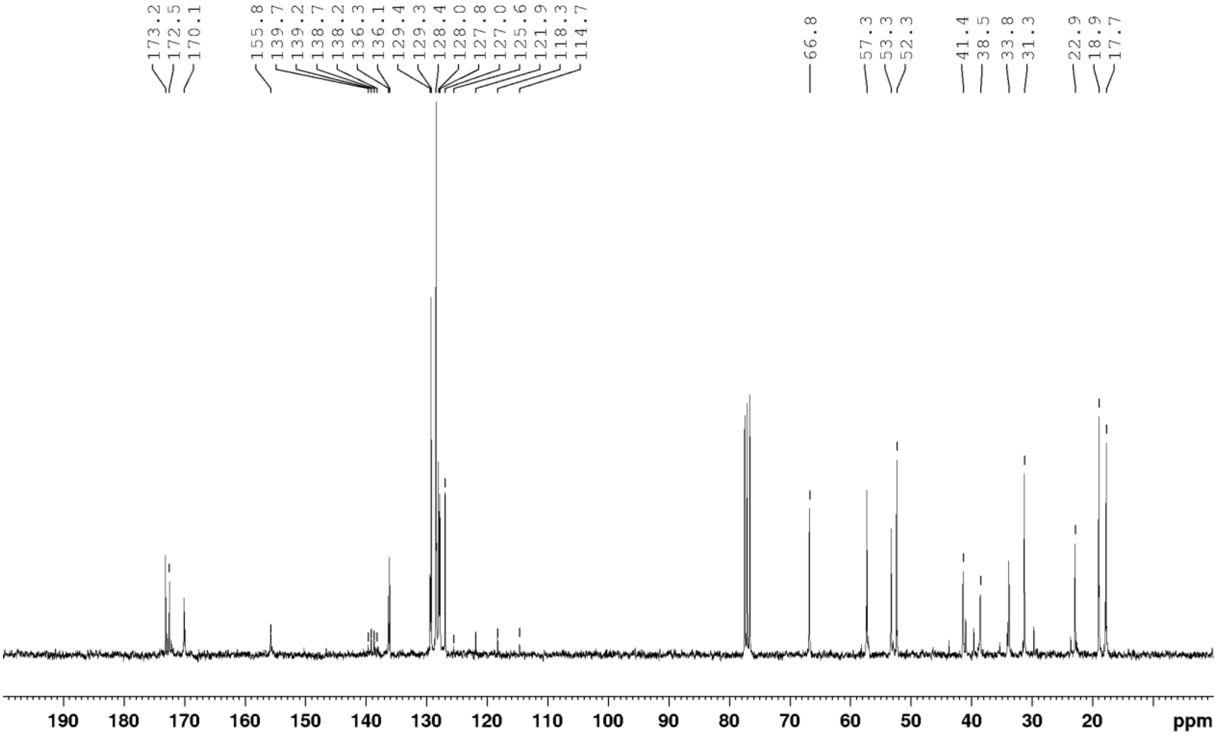
173.2
172.5
170.1
155.8
139.7
139.2
138.7
138.2
136.3
136.1
129.4
129.3
128.4
128.0
127.8
127.0
125.6
121.9
118.3
114.7
66.8
57.3
53.3
52.3
41.4
38.5
33.8
31.3
22.9
18.9
17.7
190 180 170 160 150 140 130 120 110 100 90 80 70 60 50 40 30 20 ppm

2D ^1H-^1H COSY experiments of 8e'

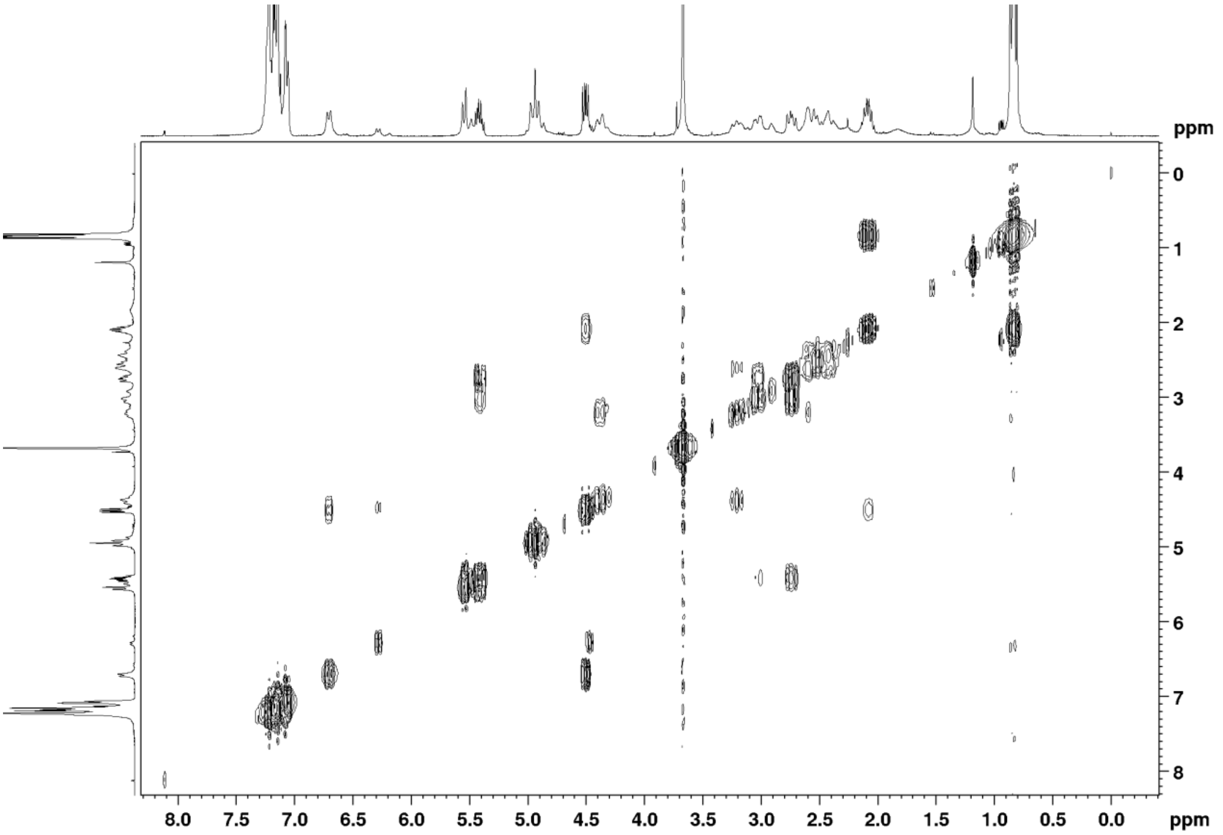

2D ^1H-^1H NOESY experiments of 8e'

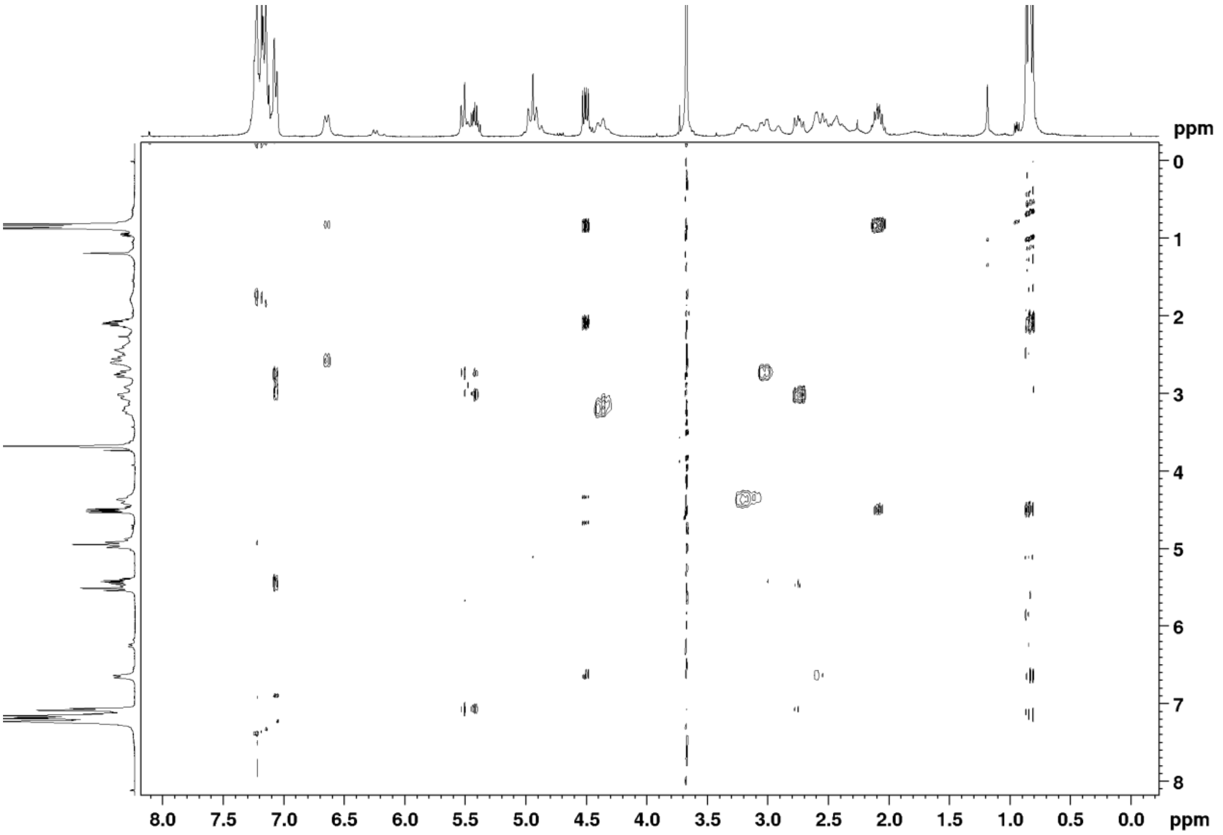

Methyl ((S)-2-(((benzyloxy)carbonyl)-L-phenylalanyl)-6-(difluoromethyl)-2,3,4,5-tetrahydropyridazine-4-carbonyl)-L-valinate (8f)

The product **8f** is obtained following the general procedure **E** as a white foam (69 mg, 52%).

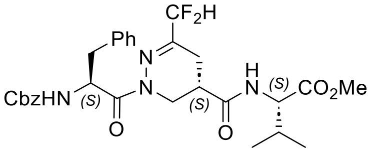

Chemical Formula: $C_{29}H_{34}F_2N_4O_6$
Exact Mass: 572,2446

^{1}H NMR (300 MHz, $CDCl_3$): δ= 7.23-7.02 (m,10H, Haro), 6.33 (d, $^3J_{H,H}$ = 8.4 Hz, 1H, NHVal), 5.87 (t, $^2J_{H,F}$ = 54.8 Hz, 1H, CF_2H), 5.59 (d, $^3J_{H,H}$ = 9.9 Hz, 1H, NHCbz), 5.49 (q, $^3J_{H,H}$ = 6.9 Hz, 1H, CHαPhe), 4.99 (s, 2H, CH_2Cbz), 4.48 (dd, $^3J_{H,H}$ = 4.9 Hz, $^3J_{H,H}$ = 8.6 Hz, 1H, CHαVal), 4.33 (dd, $^2J_{H,H}$ = 13.2 Hz, $^3J_{H,H}$ = 2.8 Hz, 1H, CH_2N), 3.66 (s, 3H, OCH_3), 3.10 (t, $^2J_{H,H}$ = 11.7 H, 1H, CH_2N), 3.02 - 2.80 (m, 2H, CH_2Ph), 2.42 (brs, 2H, CH_2), 2.26 (m, 1H, CH), 2.10 (st, $^3J_{H,H}$ = 6.5 Hz, 1H, CHiPr), 0.85 (d, $^3J_{H,H}$ = 6.5 Hz, 3H, CH_3ipr), 0.83 (d, $^3J_{H,H}$ = 6.5 Hz, 3H, CH_3iPr).

^{19}F NMR (188 MHz, $CDCl_3$): δ = -117.4 (dd, $^2J_{H,F}$ = 55.1 Hz, $^2J_{F,F}$ = 319.7 Hz, 1F), -120.9 (dd, $^2J_{H,F}$ = 54.9 Hz, $^2J_{F,F}$ = 319.8 Hz, 1F).

^{13}C NMR (75 MHz, $CDCl_3$): δ = 171.5 (CON), 171.3 (COCbz), 169.4 (CONH), 154.6 (CO), 143.2 (q, $^2J_{C,F}$ = 30.7 Hz, C=N), 135.3 (Caro), 128.4, 127.5, 127.3, 127.1, 127.0, 126.0 (CHaro), 112.7 (q, $^1J_{C,F}$ = 238 Hz, CF_2H), 65.8 (CH_2Cbz), 56.3 (CHαVal), 51.4 (CHαPhe), 51.3 (OCH_3), 40.1 (CH_2N), 38.9 (CH_2Ph), 33.1(CH), 30.2 (CHiPr), 21.1 (CH_2), 17.9 (CH_3iPr), 16.9 (CH_3iPr).

HRMS (ESI+TOF) m/z $C_{29}H_{33}F_3N_4O_6$ $[M+Na]^+$ calc. 595.2338, found 595.2350.

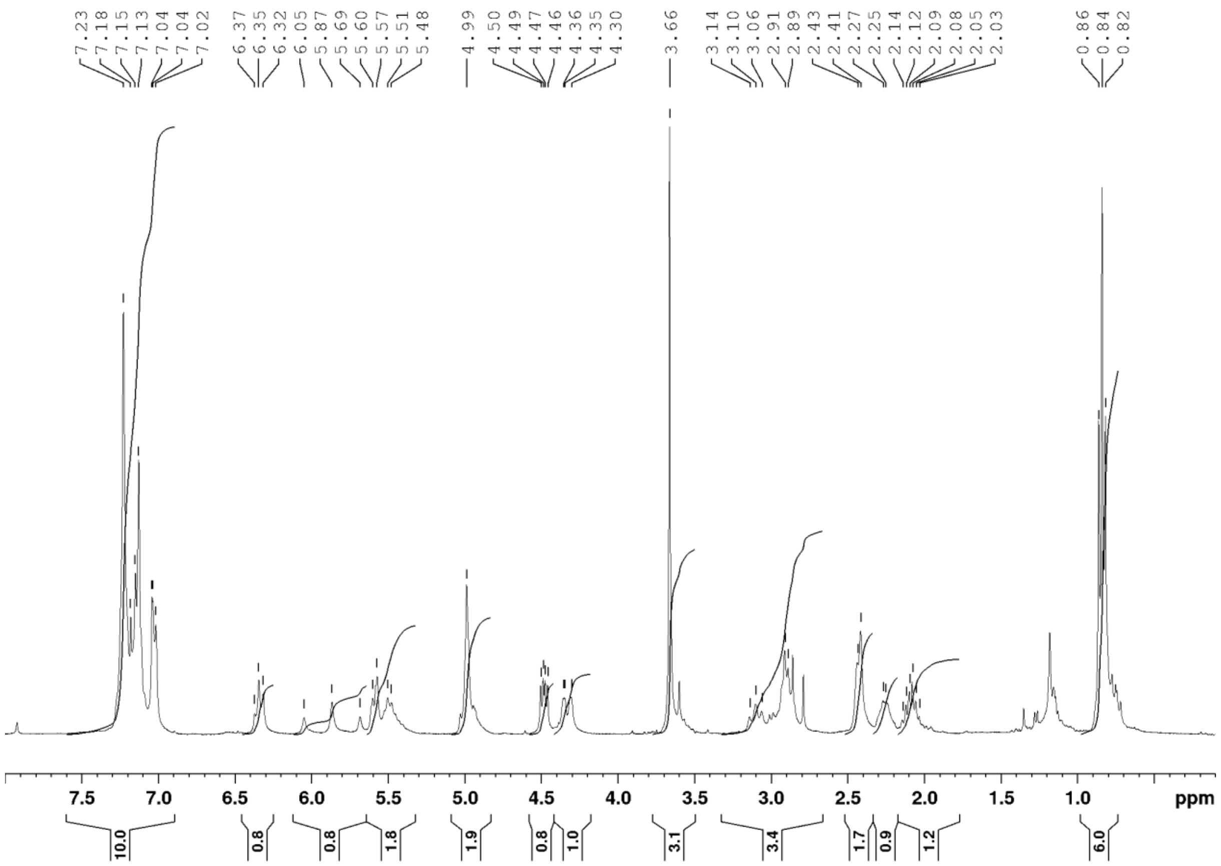

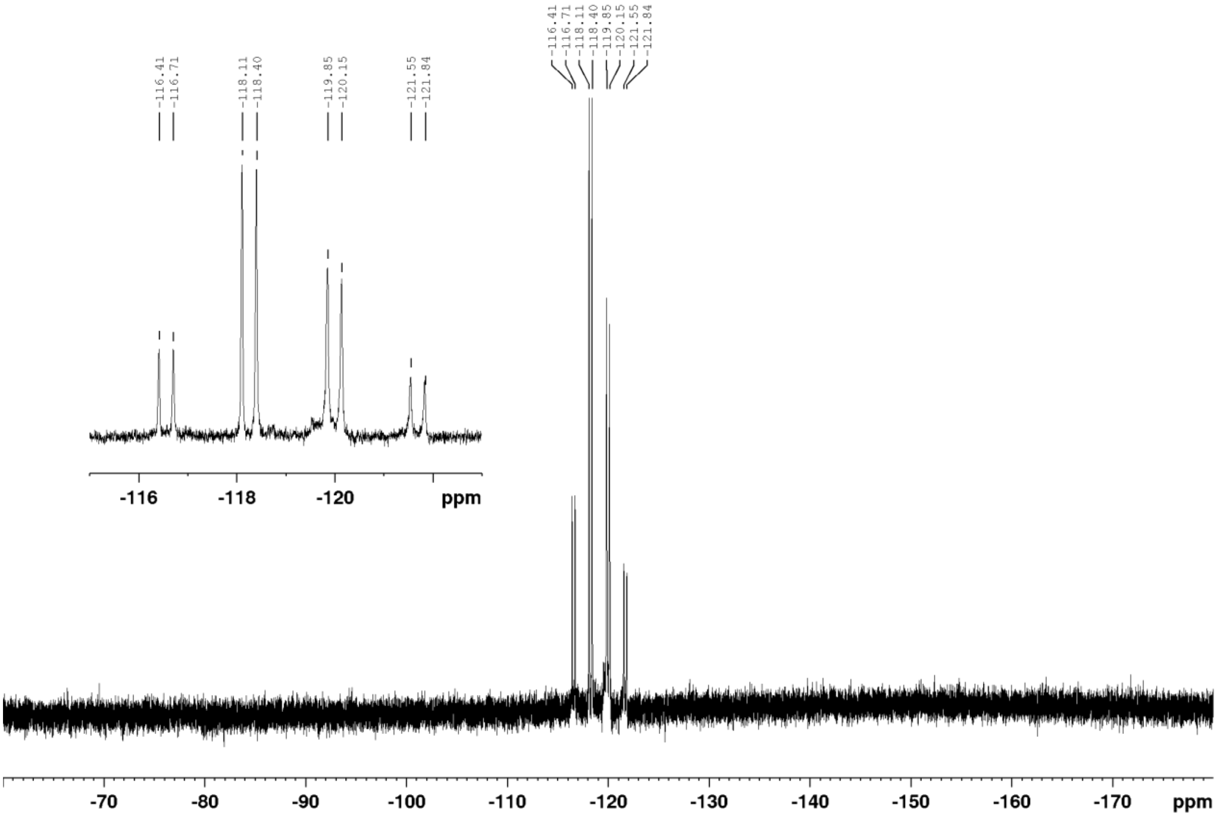

-116.41
-116.71
-118.11
-118.40
-119.85
-120.15
-121.55
-121.84
-116
-118
-120
ppm
-70
-80
-90
-100
-110
-120
-130
-140
-150
-160
-170
ppm

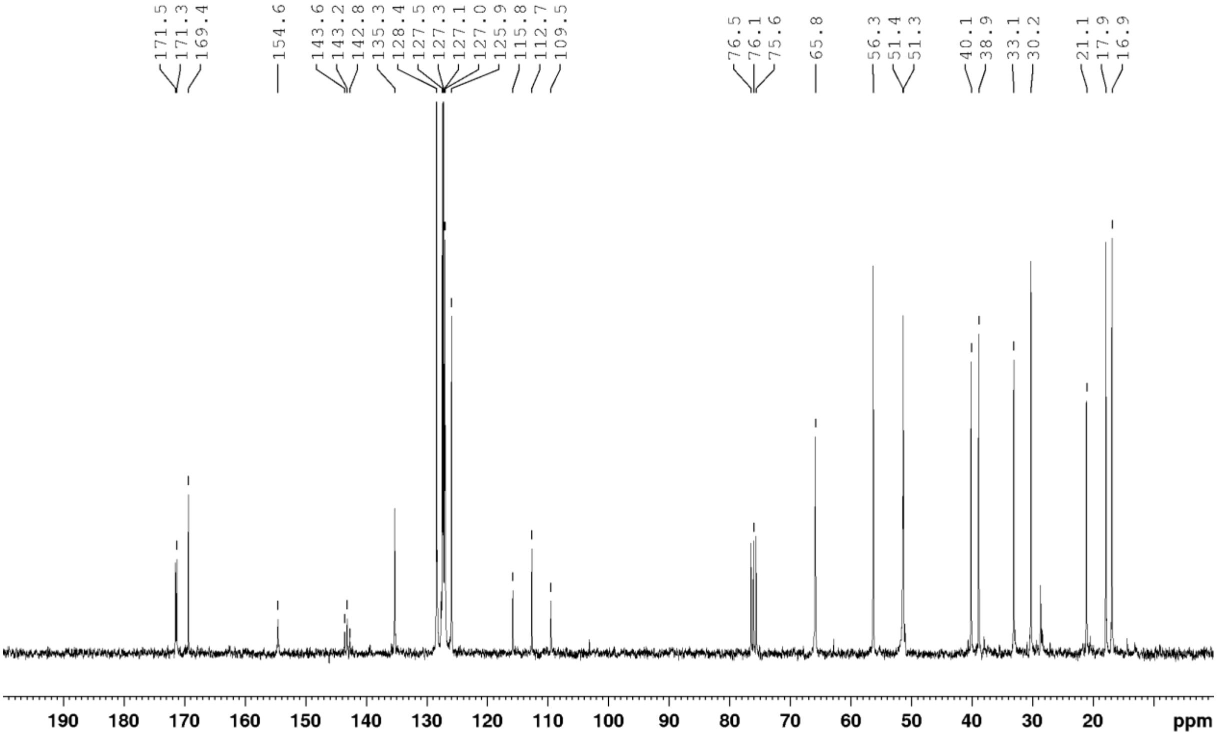

171.5
171.3
169.4
154.6
143.6
143.2
142.8
135.3
128.4
127.5
127.3
127.1
127.0
125.9
115.8
112.7
109.5
76.5
76.1
75.6
65.8
56.3
51.4
51.3
40.1
38.9
33.1
30.2
21.1
17.9
16.9
190
180
170
160
150
140
130
120
110
100
90
80
70
60
50
40
30
20
ppm

2D ^{1}H-^{1}H COSY Experiments of compound 8f:

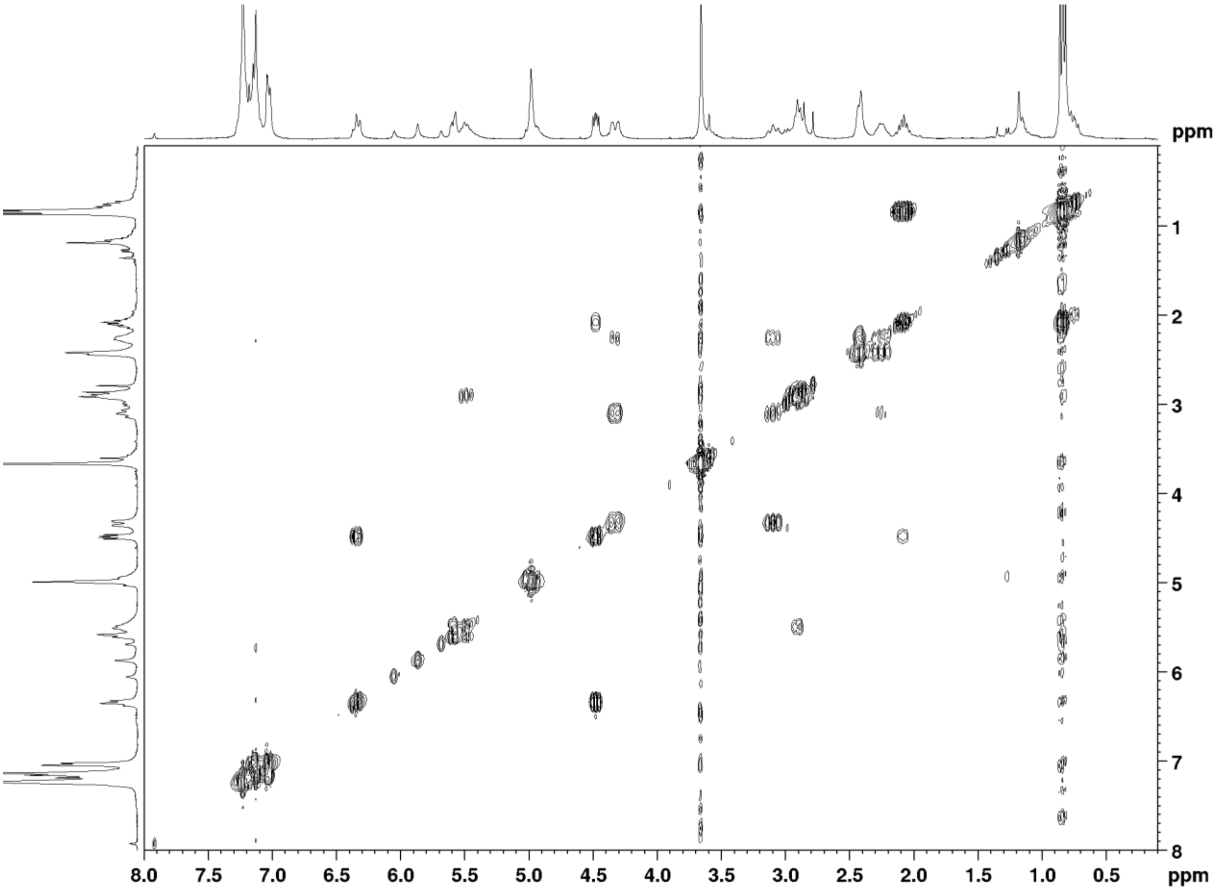

2D ^{1}H-^{1}H NOESY experiments of compound 8f

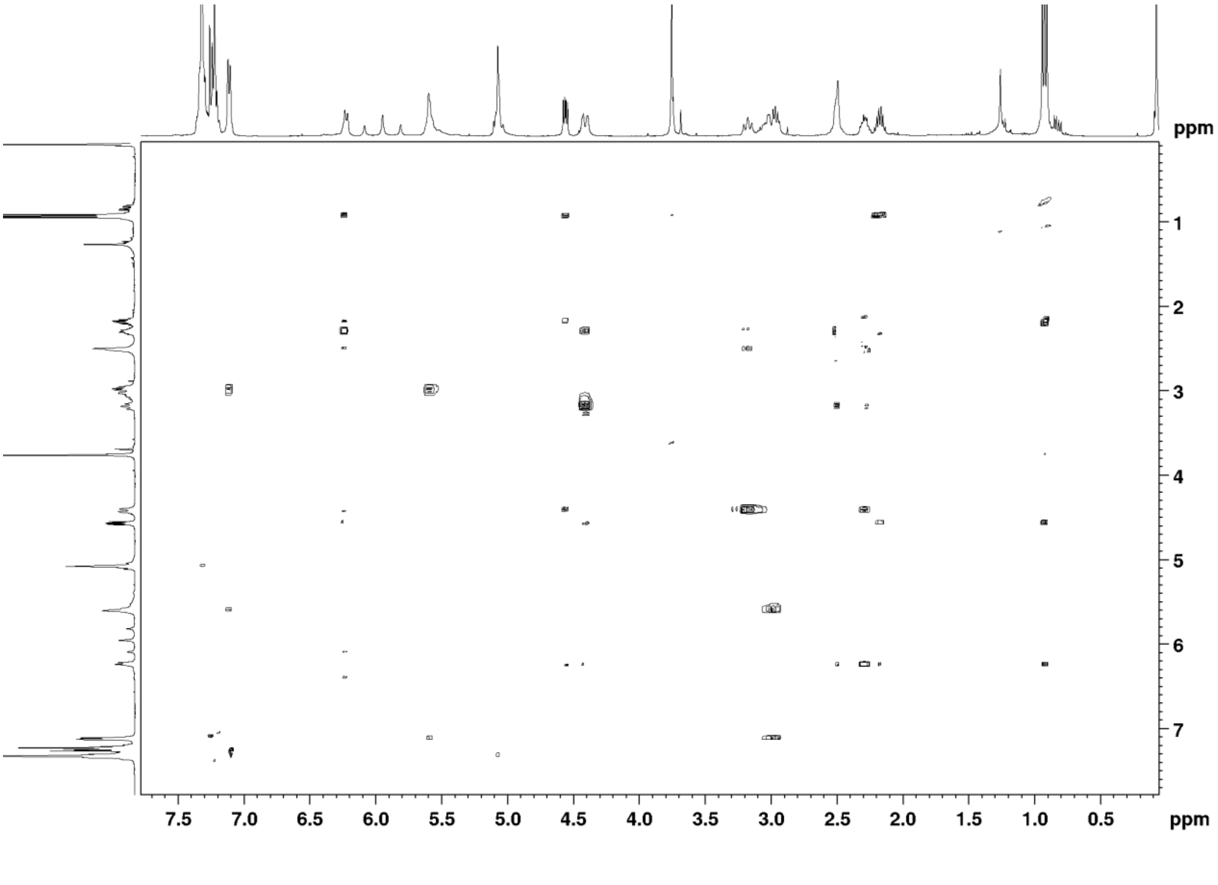

2D ^{19}F-^{1}H NOESY experiments of compound 8f

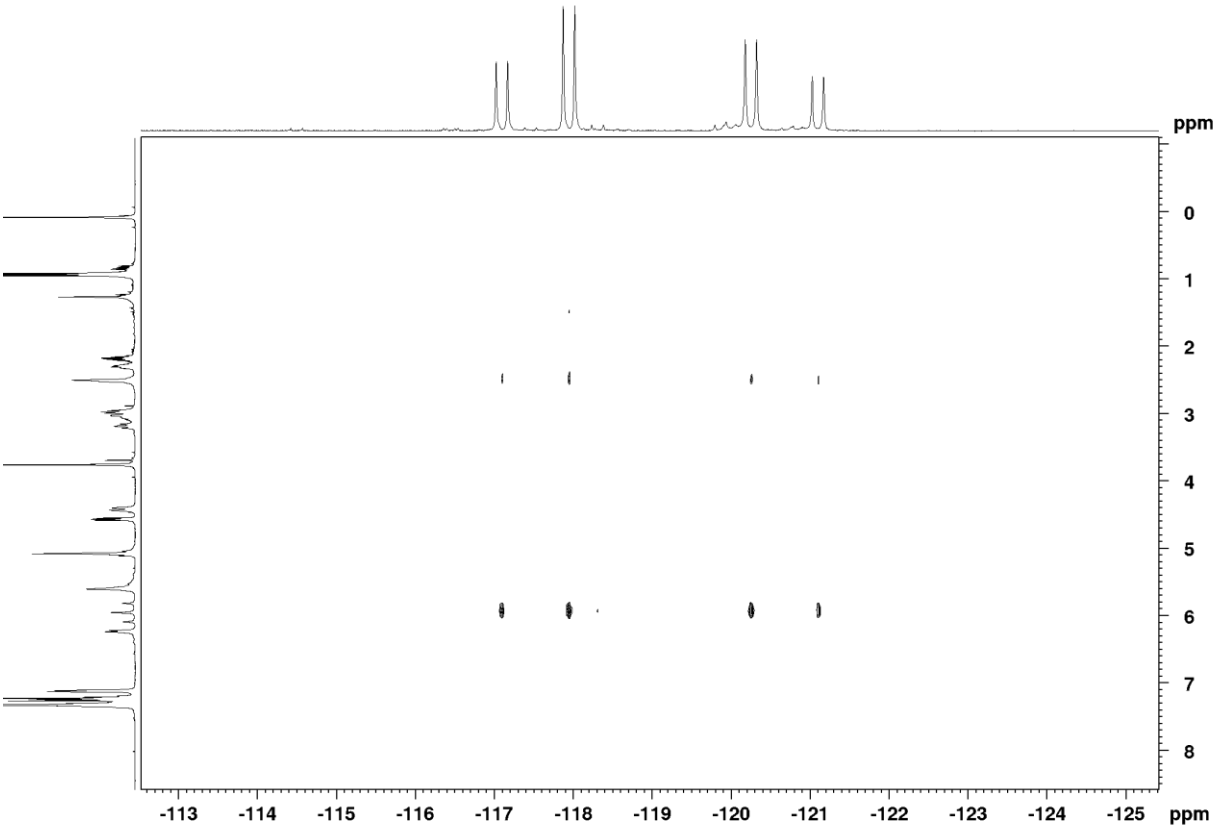

Methyl ((R)-2-(((benzyloxy)carbonyl)-L-phenylalanyl)-6-(difluoromethyl)-2,3,4,5-tetrahydropyridazine-4-carbonyl)-L-valinate (8f')

The product **8f'** is obtained following the general procedure **E** as a white foam (53 mg, 44%).

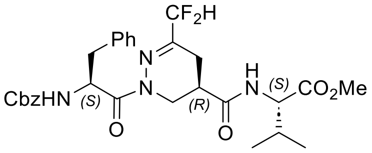

Chemical Formula: $C_{29}H_{34}F_2N_4O_6$
Exact Mass: 572,2446

^{1}H NMR (300 MHz, $CDCl_3$): δ= 7.24-7.01 (m,10H, Haro), 6.43 (d, $^3J_{H,H}$ = 8.6 Hz, 1H, NHVal), 5.87 (t, $^2J_{H,F}$ = 54.8 Hz, 1H, CF_2H), 5.50 (t, $^3J_{H,H}$ = 7.8 Hz, 1H, NHPhe), 5.44 (d, $^3J_{H,H}$ = 6.8 Hz, 1H, CHαPhe), 4.99 (d, $^2J_{H,H}$ = 12.2 Hz, 1H, CH_2Cbz), 4.93 (d, $^2J_{H,H}$ = 12.3 Hz, 1H, CH_2Cbz), 4.51 (dd, $^3J_{H,H}$ = 4.96 Hz, $^3J_{H,H}$ = 8.8 Hz, 1H, CHαVal), 4.36 (d, $^2J_{H,H}$ = 13.2 Hz, 1H, CH_2N), 3.67 (s, 3H, OCH_3), 3.18 (t, $^2J_{H,H}$ = 11.2 H, 1H, CH_2N), 2.97 (dd, $^2J_{H,H}$ = 13.5 Hz, $^3J_{H,H}$ = 5.6 Hz, 1H, CH_2Ph), 2.83 (dd, $^2J_{H,H}$ = 13.4 Hz, $^3J_{H,H}$ = 6.7 Hz, 1H, CH_2Ph), 2.53 (m, 1H, CH), 2.45 (d, $^3J_{H,H}$ = 6.0 Hz, 2H, CH_2), 2.06 (dst, $^3J_{H,H}$ = 8.2 Hz, $^3J_{H,H}$ = 6.8 Hz, 1H, CHiPr), 0.86 (d, $^3J_{H,H}$ = 6.8 Hz, 3H, CH_3iPr), 0.82 (d, $^3J_{H,H}$ = 6.9 Hz, 3H, CH_3iPr).

^{19}F NMR (188 MHz, $CDCl_3$): δ = -117.7 (dd, $^2J_{H,F}$ = 56.4 Hz, $^2J_{F,F}$ = 318.9 Hz, 1F), -120.6 (dd, $^2J_{H,F}$ = 54.6 Hz, $^2J_{F,F}$ = 318.6 Hz, 1F).

^{13}C NMR (75 MHz, $CDCl_3$): δ = 172.7 (CON), 172.4 (COCbz), 170.4 (CONH), 155.7 (CO), 144.5 (q, $^2J_{C,F}$ = 30.8 Hz, C=N), 136.9, 136.15 (Caro), 129.4, 128.5, 128.4, 128.1, 127.9, 127.0 (CHaro), 113.6 (q, $^1J_{C,F}$ = 238.2 Hz, CF_2H), 66.8 (CH_2Cbz), 57.2 (ChαVal), 52.6 (CHαPhe), 52.3 (OCH_3), 41.6 (CH_2N), 39.0 (CH_2Ph), 34.0 (CHiPr), 31.3 (CH), 21.4 (CH_2), 18.9 (CH_3iPr), 17.8 (CH_3iPr).

HRMS (ESI+TOF) m/z C29H33F3N4O6Na $[M+Na]^+$ calc. 595.2338, found 595.2350.

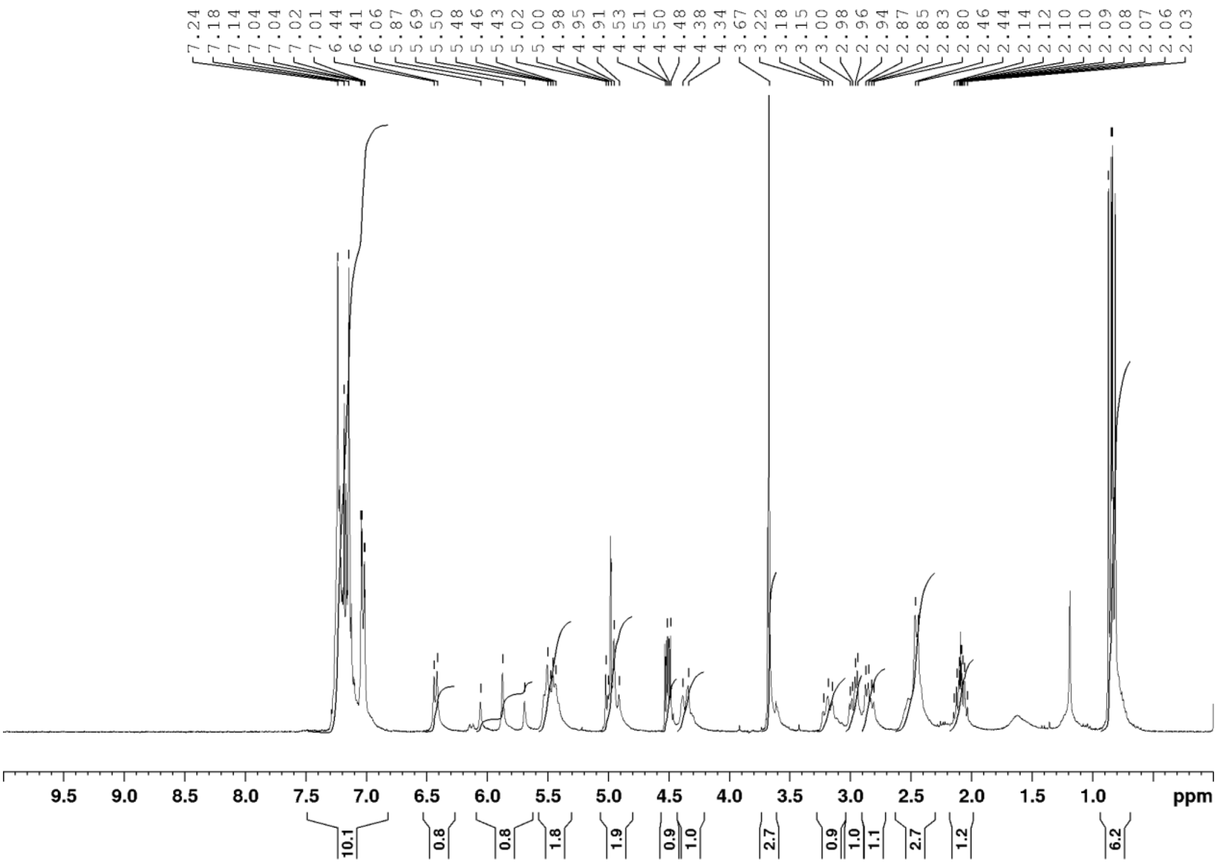

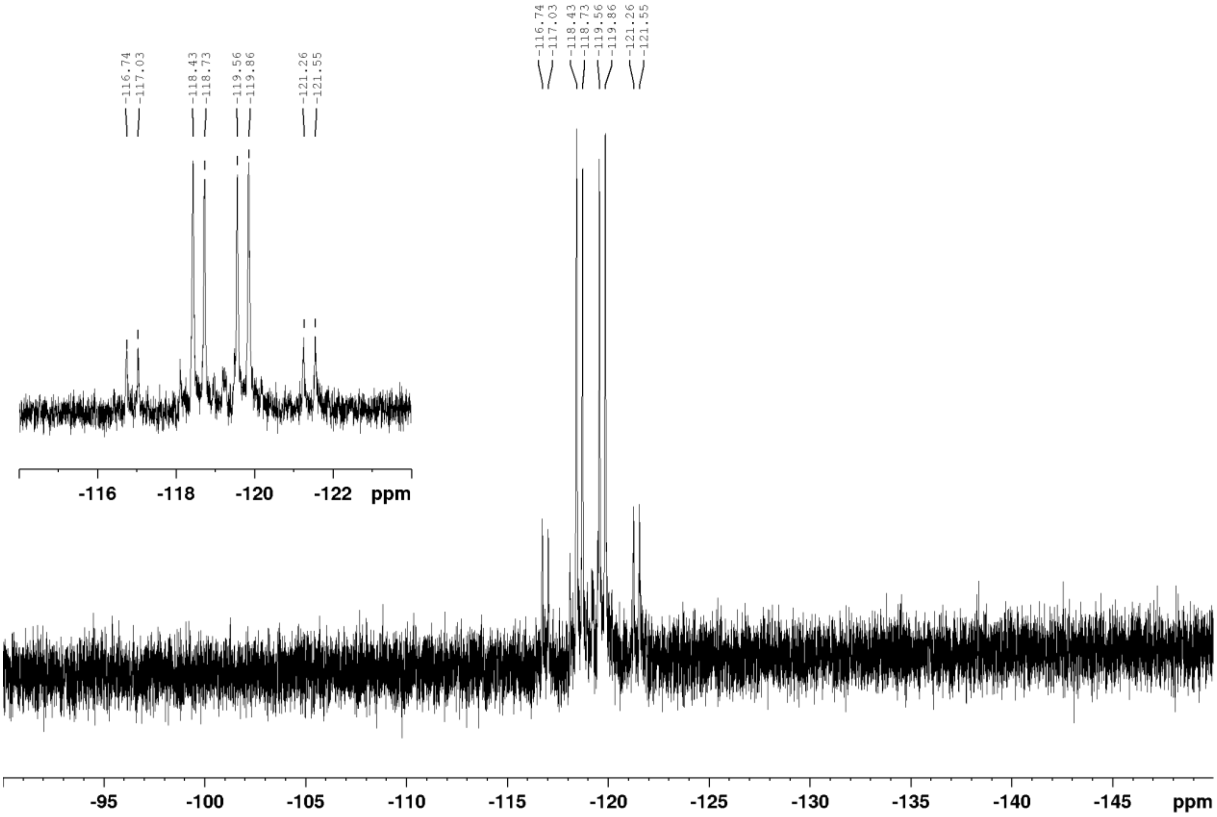

-116.74
-117.03
-118.43
-118.73
-119.56
-119.86
-121.26
-121.55
-116
-118
-120
-122
ppm
-95
-100
-105
-110
-115
-120
-125
-130
-135
-140
-145
ppm

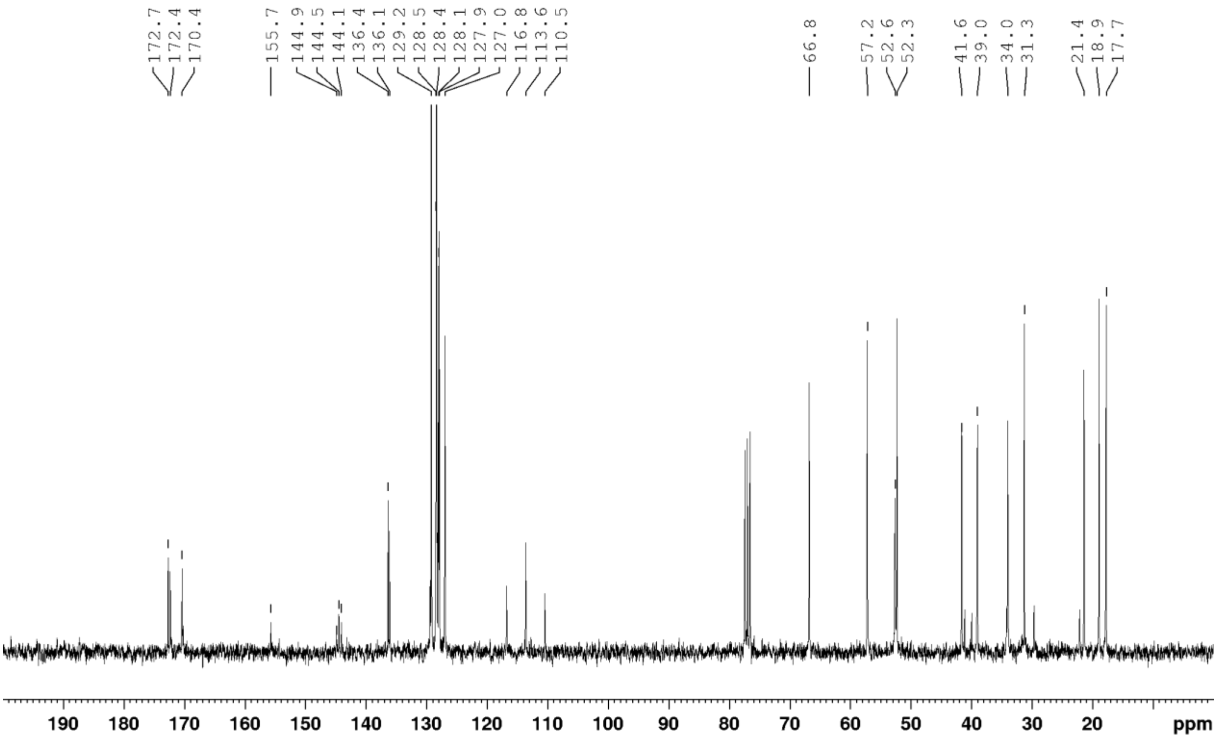

172.7
172.4
170.4
155.7
144.9
144.5
144.1
136.4
136.1
129.2
128.5
128.4
128.1
127.9
127.0
116.8
113.6
110.5
66.8
57.2
52.6
52.3
41.6
39.0
34.0
31.3
21.4
18.9
17.7
190
180
170
160
150
140
130
120
110
100
90
80
70
60
50
40
30
20
ppm

2D ^{1}H-^{1}H COSY experiments of compound 8f'

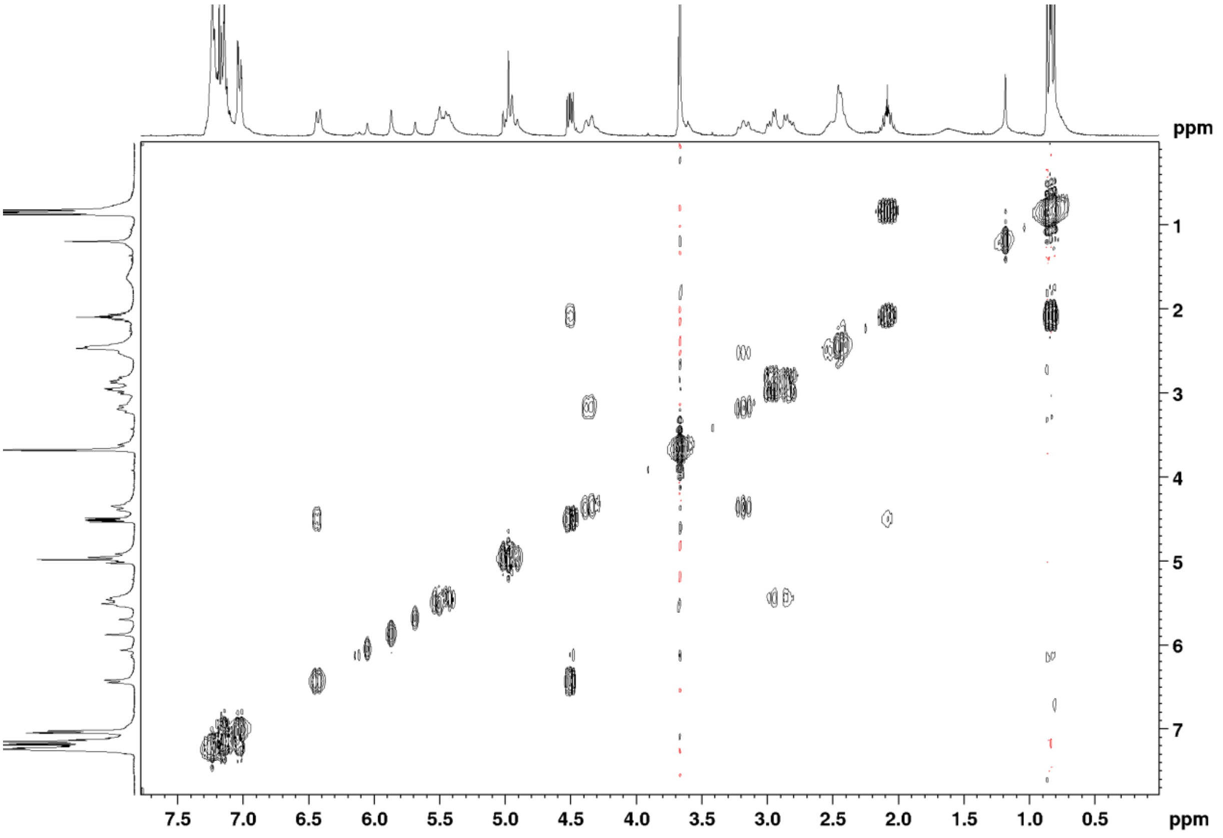

2D ^{1}H-^{1}H NOESY experiments of compound 8f'

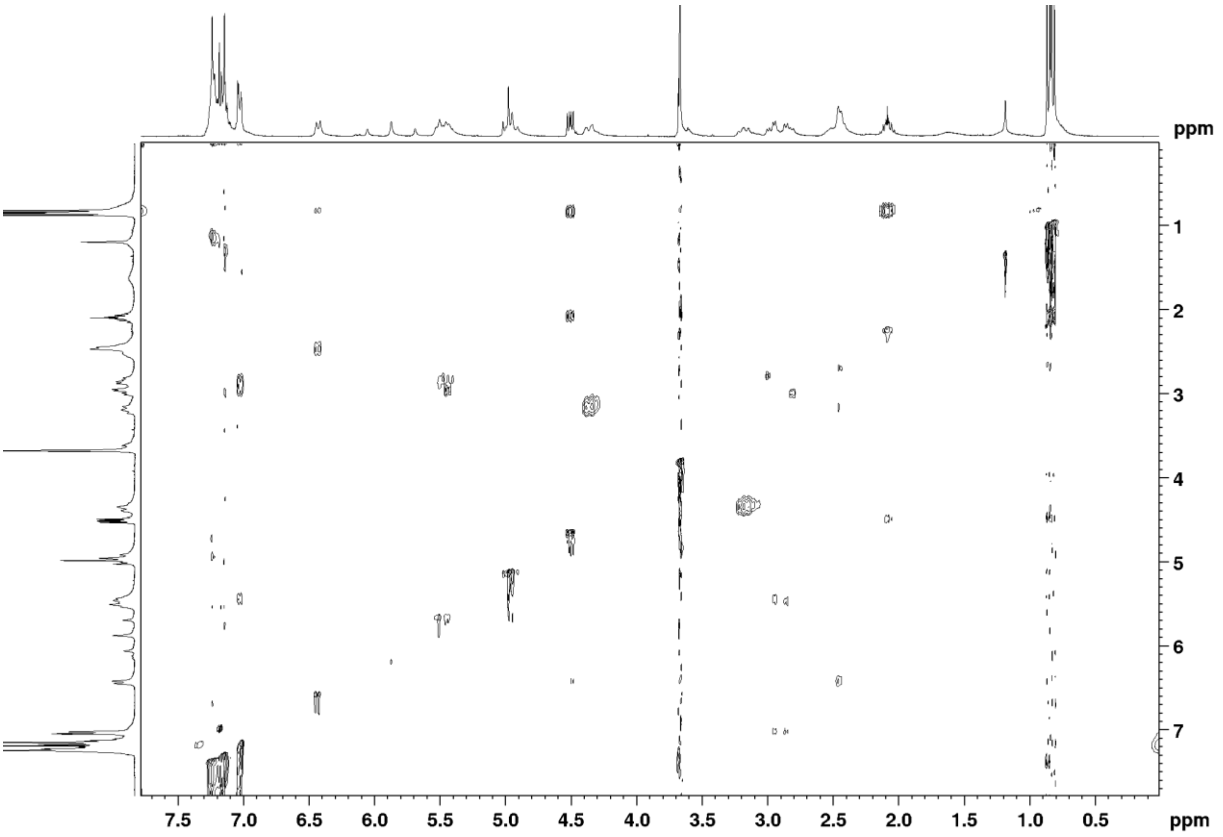

f) X-ray analysis of 8f:

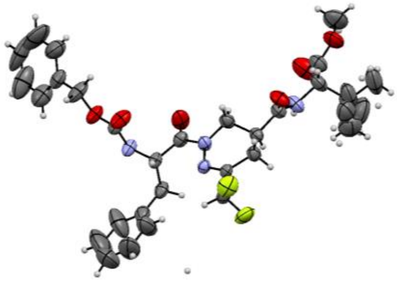

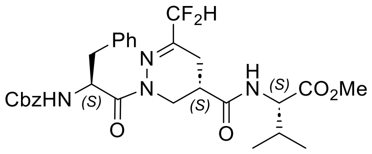

Chemical Formula: $C_{29}H_{34}F_2N_4O_6$
Exact Mass: 572,2446

Bond precision: C-C = 0.0035 A Wavelength=0.71073

Cell:	a=15.8351(12)	b=5.0455(4)	c=17.8221(13)
	alpha=90	beta=94.756(7)	gamma=90
Temperature:	173 K		

	Calculated	Reported
Volume	1419.01(19)	1419.01(19)
Space group	P 21	P 21
Hall group	P 2yb	P 2yb
Moiety formula	C29 H34 F2 N4 O6	C29 H34 F2 N4 O6
Sum formula	C29 H34 F2 N4 O6	C29 H34 F2 N4 O6
Mr	572.60	572.60
Dx,g cm-3	1.340	1.340
Z	2	2
Mu (mm-1)	0.103	0.103
F000	604.0	604.0
F000'	604.33	
h,k,lmax	21,6,24	21,6,24
Nref	7645[4238]	6500
Tmin,Tmax	0.996,0.998	0.756,1.000
Tmin'	0.977	

Correction method= # Reported T Limits: Tmin=0.756 Tmax=1.000
AbsCorr = GAUSSIAN

Data completeness= 1.53/0.85 Theta(max)= 29.183

R(reflections)= 0.0435(5145) wR2(reflections)= 0.1079(6500)

S = 1.069 Npar= 541

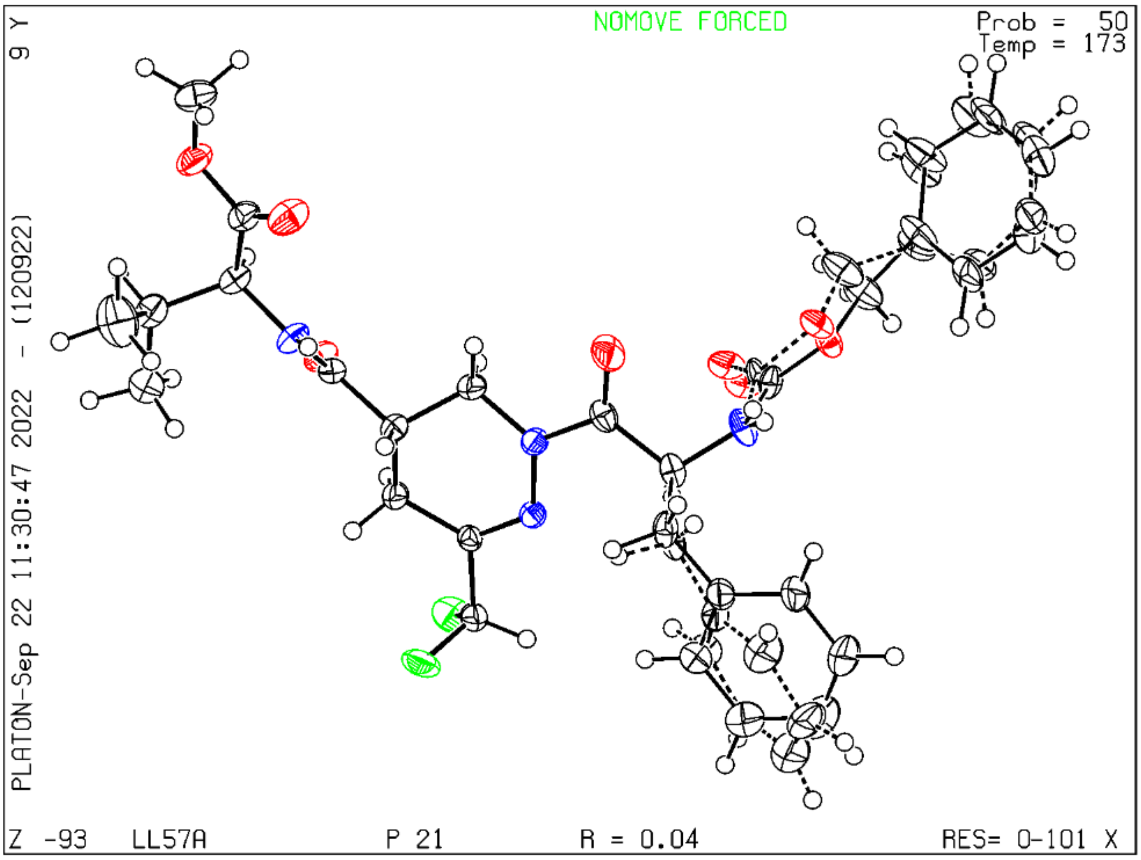

g) ^{1}H NMR spectra comparison of 7f,f' and 7e,e' for configuration assignment.

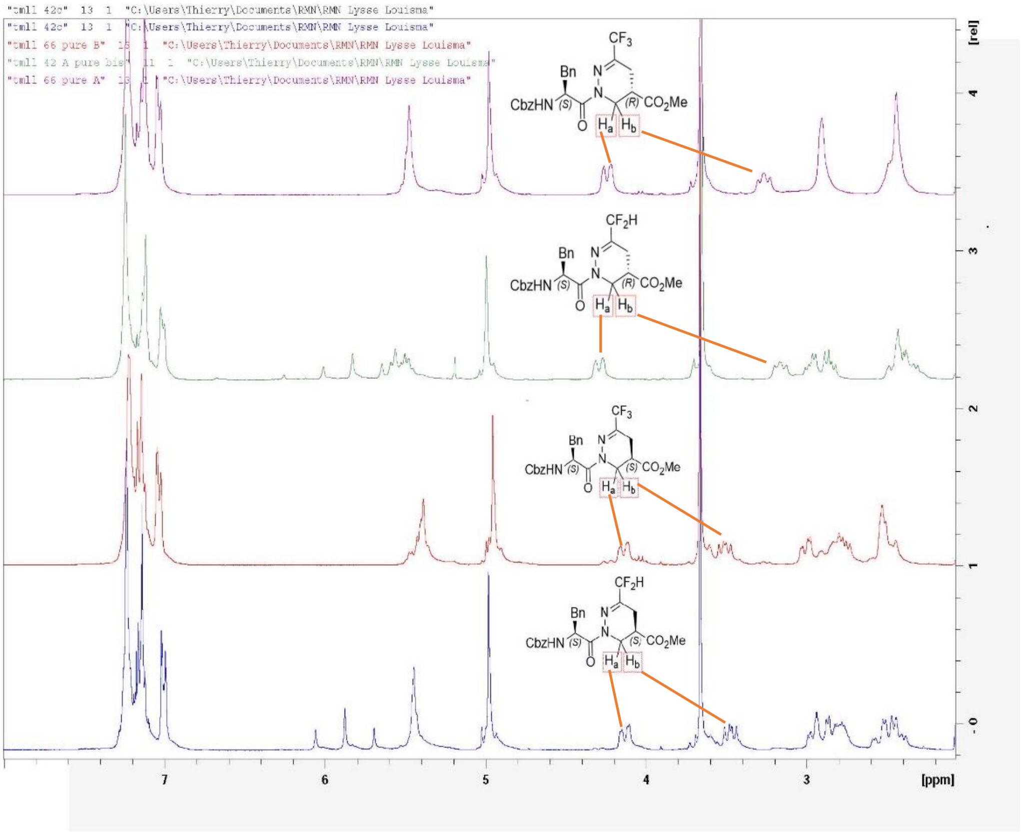

Supplement: File 1 — Experimental procedures, product characterization, X-ray analysis and copies of NMR spectra. [file Beilstein_J_Org_Chem-20-3174-s001.pdf]
